# Structure–Activity Relationship Studies of Indolglyoxyl-Polyamine Conjugates as Antimicrobials and Antibiotic Potentiators

**DOI:** 10.3390/ph16060823

**Published:** 2023-05-31

**Authors:** Melissa M. Cadelis, Tim Liu, Kenneth Sue, Florent Rouvier, Marie-Lise Bourguet-Kondracki, Jean Michel Brunel, Brent R. Copp

**Affiliations:** 1 School of Chemical Sciences, The University of Auckland, Private Bag 92019, Auckland 1142, New Zealand; 2 School of Medical Sciences, The University of Auckland, Private Bag 92019, Auckland 1142, New Zealand; 3 UMR MD1 “Membranes et Cibles Thérapeutiques”, U1261 INSERM, Faculté de Pharmacie, Aix-Marseille Université, 27 bd Jean Moulin, 13385 Marseille, France; 4 Laboratoire Molécules de Communication et Adaptation des Micro-organismes, UMR 7245 CNRS, Muséum National d’Histoire Naturelle, 57 Rue Cuvier (C.P. 54), 75005 Paris, France

**Keywords:** indole, potentiator, antimicrobial, polyamine, antibiotics, antifungal agents, structure–activity relationships

## Abstract

Antibiotic resistance is a growing global health threat, requiring urgent attention. One approach to overcome antibiotic resistance is to discover and develop new antibiotic enhancers, molecules that work with legacy antibiotics to enhance their efficacy against resistant bacteria. Our previous screening of a library of purified marine natural products and their synthetic analogues led to the discovery of an indolglyoxyl-spermine derivative that exhibited intrinsic antimicrobial properties and was also able to potentiate the action of doxycycline towards the difficult to treat, Gram-negative bacterium *Pseudomonas aeruginosa*. A set of analogues have now been prepared, exploring the influence of indole substitution at the 5- and 7- positions and length of the polyamine chain on biological activity. While limiting cytotoxicity and/or hemolytic activities were observed for many analogues, two 7-methyl substituted analogues (**23b** and **23c**) were found to exhibit strong activity towards Gram-positive bacteria with no detectable cytotoxicity or hemolytic properties. Different molecular attributes were required for antibiotic enhancing properties, with one example identified, a 5-methoxy-substitiuted analogue (**19a**), as being a non-toxic, non-hemolytic enhancer of the action of two tetracycline antibiotics, doxycycline and minocycline, towards *P. aeruginosa*. These results provide further stimulation for the search for novel antimicrobials and antibiotic enhancers amongst marine natural products and related synthetic analogues.

## 1. Introduction

The global increase in microbial antibiotic resistance is a growing health threat, requiring urgent attention. With only limited numbers of new antibiotics being approved for clinical use [1,2,3] the search is on for novel strategies that can prove effective against drug-resistant pathogens. One option for treatment is to restore the antibiotic action of legacy antibiotics, requiring the discovery of antibiotic adjuvants or enhancers [4,5,6,7,8]. Marine natural products represent an excellent reservoir of small drug-like molecules from which to discover both new classes of antimicrobial agents [9,10,11] as well as antibiotic enhancers [8,12,13,14].

**Figure 1:**
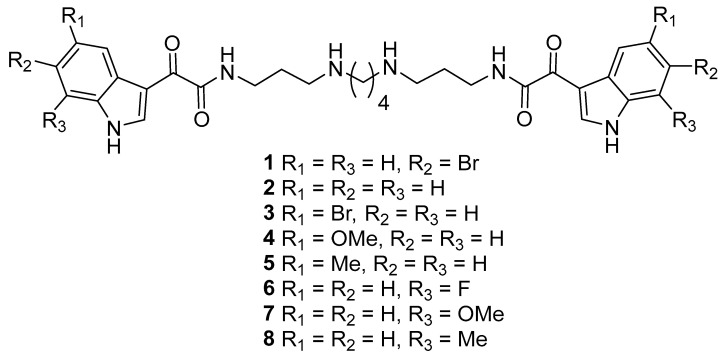
Structures of indolglyoxyl spermine derivatives.

Our screening of a library of marine natural product-related α,ω-disubstituted spermine analogues for antimicrobial and antibiotic enhancing properties identified the 6-bromoindolglyoxyl derivative **1** (Figure 1) as a moderately active antimicrobial towards the Gram-positive bacteria *Staphylococcus aureus* ATCC 25923 (MIC 6.25 µM) and the fungus *Cryptococcus neoformans* (MIC 1.1 µM). In addition, the combination of **1** with doxycycline exhibited a strong antibiotic enhancement effect towards the Gram-negative bacterium *Pseudomonas aeruginosa* [15]. Interest in these activities was somewhat tempered by the observation of associated cytotoxicity (human embryonic kidney cell line HEK293, IC_50_ 5.1 µM; rat skeletal myoblast cell line L6, IC_50_ 7.7 µM), prompting the search for less toxic analogues. Subsequent studies identified the requirement of substitution on the indole ring for activity, with **2** being inactive as an antimicrobial or antibiotic enhancer, and that some examples of 5- and 7- substituted analogues (**3**–**8**), notably including halogen, methoxy or methyl functionality, exhibited more modest antimicrobial activities (Table 1), were moderate to excellent antibiotic enhancers (Table 2) and were generally less cytotoxic and non-hemolytic (Table 3) [16].

**Figure 2:**
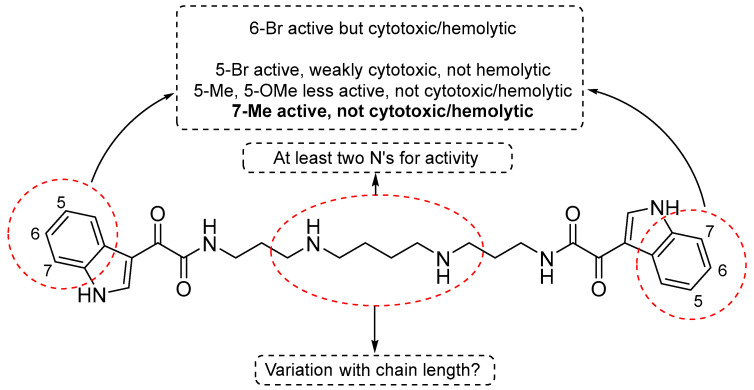
Antibiotic enhancement structure–activity relationship for indolglyoxyl spermine derivatives.

Taken together, these studies enabled the identification of the structural requirements for antibiotic enhancement properties amongst a limited set of indolglyoxyl-spermine conjugates, summarized in Figure 2.

A component of the structure–activity relationship yet to be addressed in this compound series is the effect, if any, of variation in the polyamine (PA) chain length on intrinsic antimicrobial, antibiotic enhancement and cytotoxicity/hemolysis biological activities. Previous studies investigating disubstituted polyamine-bearing arylacyl [17] head groups identified that changes in the chain length of the core polyamine fragment can lead to wide variation in antimicrobial and/or antibiotic enhancing properties. Herein we report details on the synthesis of a new set of indolglyoxyl-polyamine conjugates that vary in substitution at the 5- and 7- positions on the indole ring and that vary in polyamine chain length, and the abilities of these analogues to exhibit intrinsic antimicrobial properties and to potentiate the activity of doxycycline towards the Gram-negative bacteria *Pseudomonas aeruginosa*.

## 2. Results and Discussion

### 2.1. Chemistry

**Figure 3:**
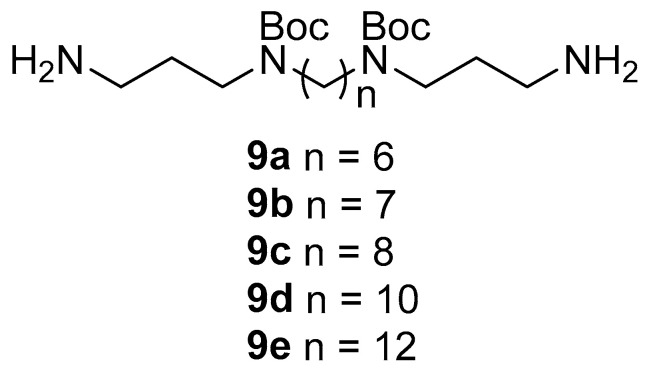
Polyamine scaffolds **9a**–**e**.

The Boc-protected polyamine scaffolds used in this study were the five examples **9a**–**e** covering core chain lengths of 6, 7, 8, 10 and 12 methylenes (Figure 3). The preparation of **9a**–**e** has been previously described [18,19,20,21].

**Figure 4:**
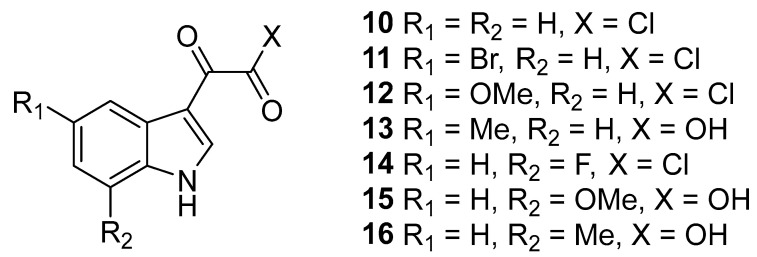
Indole-3-glyoxyl head groups **10**–**16**.

The indole-3-glyoxyl head groups used in the current study (**10**–**16**) (Figure 4) were the same set previously explored in analogues **2**–**8** [16]. Syntheses of **10**–**15**, as either the glyoxylic acid or glyoxylchloride, have been previously reported [22,23,24,25].

**Scheme 1:**
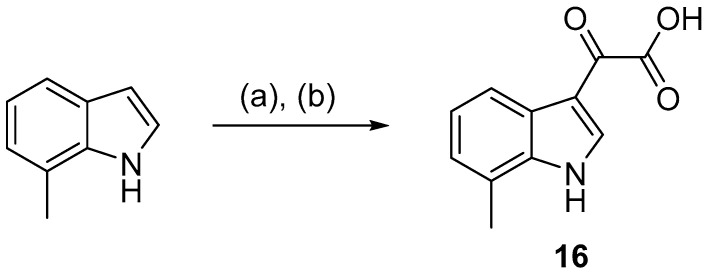
Synthesis of 2-(7-methyl-1*H*-indol-3-yl)-2-oxoacetic acid (**16**). Reagents and conditions: (**a**) oxalyl chloride, Et_2_O, 0 °C, 1.5 h; and (**b**) saturated aq. NaHCO_3_, reflux, 2 h (95% over two steps).

In the case of the 7-methyl analogue **16,** it was prepared using the two-step protocol shown in Scheme 1. Reaction of 7-methyl-1*H*-indole with excess oxalyl chloride afforded the oxalylchloride intermediate which was not isolated but hydrolyzed by heating with saturated aq. NaHCO_3_ solution to afford 2-(7-methyl-1*H*-indol-3-yl)-2-oxoacetic acid (**16**) (Figure S1) in 95% yield over two steps.

The reaction of indole-3-glyoxyl chlorides **10**–**12** and **14** directly with Boc-protected polyamines **9a**–**e**, or glyoxylic acids **13**, **15** and **16** with **9a**–**e** utilizing the coupling reagent PyBOP (benzotriazol-1-yloxytripyrrolidinophosphonium hexafluorophosphate) afforded a set of intermediate products that were then deprotected with 2,2,2-trifluoroacetic acid (TFA) to afford the desired compounds as their di-TFA salts (Scheme 2, Figures S2–S30).

### 2.2. Biological Evaluation

The antimicrobial activity of the series was evaluated against a range of bacterial strains (*S. aureus*, MRSA, *P. aeruginosa* and *Escherichia coli*) and the fungus *Candida albicans* (Table 1). Cytotoxicity towards HEK293 (human kidney epithelial cell line, IC_50_) and hemolytic activity against human red blood cells (HC_10_) were also determined.

**Table 1:** Antimicrobial (MIC), cytotoxicity (IC_50_) and hemolytic (HC_10_) activities (µM) of analogues **2**–**8**, **17**–**23**.

Compound	*S. a* ^a^	*MRSA* ^b^	*P. a* ^c^	*E. c* ^d^	*C. a* ^e^	*Cyto.* ^f^	*Hem.* ^g^
**2**	>100 ^h^	41.4	>100 ^h^	>100 ^h^	>41.4 ^h^	>41 ^h^	>41
**3**	3.125 ^h^	4.32	50 ^h^	25 ^h^	>34.4 ^h^	14 ^h^	>34
**4**	100 ^h^	38.4	>200 ^h^	>200 ^h^	>38.4 ^h^	>38 ^h^	>38
**5**	25 ^h^	20.0	100 ^h^	200 ^h^	>40.0 ^h^	>40 ^h^	>40
**6**	25 ^h^	19.8	200 ^h^	200 ^h^	>39.6 ^h^	19 ^h^	>40
**7**	15 ^h^	38.4	>200 ^h^	>200 ^h^	38.4 ^h^	27 ^h^	>38
**8**	25 ^h^	20.0	>200 ^h^	>200 ^h^	>40.0 ^h^	>40 ^h^	>40
**17a**	12.5	>40	>200	50	>40	n.d ^i^	n.d
**17b**	25	39	>200	25	>39	31	>39
**17c**	25	39	100	12.5	>39	10	>39
**17d**	14.6	18.7	117	117	37	4.9	>37
**17e**	25	9.04	>200	>200	36	5.6	>36
**18a**	1.56	4.17	50	6.25	16.7	8.9	>33
**18b**	6.4	4.1	51	51	16	13	>33
**18c**	25	8.1	>200	>200	32	18	>32
**18d**	6.16	7.88	200	98	32	16	>32
**18e**	5.99	3.84	96	24	31	8.6	20
**19a**	29	>37	>200	>200	>37	>37	>37
**19b**	57	37	>200	>200	37	>37	>37
**19c**	>100	>36	>100	>100	36	18	26
**19d**	12.5	17.4	>200	50	35	6.8	>35
**19e**	12.5	34	>200	100	34	8.6	>34
**20a**	12.5	≤0.30	200	50	>39	>39	n.d.
**20b**	6.25	≤0.30	200	50	>38	n.d.	>38
**20c**	3.125	4.7	800	12.5	>37	>37	n.d.
**20d**	3.125	≤0.28	800	12.5	9.0	>36	n.d.
**20e**	3.125	n.d.	800	12.5	2.2	n.d.	2.0
**21a**	7.5	19.1	240	240	>38	12	>38 ^e^
**21b**	7.3	18.8	>200	>200	38	11	>38
**21c**	29	18.5	>200	>200	>37	9.2	>37
**21d**	3.125	4.48	>200	6.25	36	4.7	>36
**21e**	27.2	4.34	>220	>220	35	6.4	>35
**22a**	50	>37	800	400	>37	>37	>37
**22b**	25	>37	800	200	>37	8.9	>37
**22c**	25	4.5	800	200	>36	n.d.	>36
**22d**	25	≤0.27	800	50	17	>35	8.4
**22e**	3.125	≤0.26	800	12.5	≤0.26	n.d.	0.93
**23a**	30.2	≤0.30	>240	60	>39	>39	>39
**23b**	14.8	≤0.30	>240	120	>38	>38	>38
**23c**	7.3	≤0.29	>230	29	>37	>37	>37
**23d**	7.1	≤0.28	>230	14.1	18	>36	n.d.
**23e**	6.85	≤0.27	>220	13.7	≤0.27	>35	8.4

^a^ *S. aureus* ATCC 25923 with streptomycin (MIC 21.5 µM) and chloramphenicol (MIC 1.5–3 µM) used as positive controls and values presented as the mean (n = 3); ^b^ MRSA ATCC 43300 with vancomycin (MIC 0.7 μM) used as a positive control and values presented as the mean (n = 2); ^c^ *P. aeruginosa* ATCC 27853 with streptomycin (MIC 21.5 µM) and colistin (MIC 1 µM) used as positive controls and values presented as the mean (n = 3); ^d^ *E. coli* ATCC 25922 with streptomycin (MIC 21.5 µM) and colistin (MIC 2 µM) used as positive controls and values presented as the mean (n = 3); ^e^ *C. albicans* ATCC 90028 with fluconazole (MIC 0.4 μM) as a positive control and values presented as the mean (n = 2); ^f^ Concentration of compound at 50% cytotoxicity on HEK293 (human embryonic kidney cells) with tamoxifen as the positive control (IC_50_ 24 µM) and values presented as the mean (n = 2); ^g^ Concentration of compound at 10% hemolytic activity on human red blood cells with melittin as the positive control (HC_10_ 0.95 μM) and values presented as the mean (n = 2); ^h^ Data taken from Cadelis et al. [16]; ^i^ n.d., not determined.

In general, the compound set exhibited more pronounced activity towards the Gram-positive bacteria *S. aureus* ATCC 25923 and MRSA, with only poor or no activity towards Gram-negative bacteria *P. aeruginosa* and *E. coli* and the fungus *C. albicans*. Amongst the more active examples identified were the 5-bromo substituted analogues **18a**–**e** with *S. aureus* and MRSA MIC 1.6–7.8 µM, 5-methyl analogues **20a**–**d** (MIC ≤ 0.28–6.25 µM), 7-methoxy analogue **22e** (MIC ≤ 0.26–3.125 µM), and 7-methyl substituted variants **23c**–**e** (MIC ≤ 0.27–7.3 µM). In many cases, those analogues that exhibited good levels of antimicrobial activity also unfortunately demonstrated cytotoxicity and/or hemolytic activity. There were some examples identified, however, that were devoid of these detrimental properties including the 7-methyl substituted analogues **23b** (MIC MRSA ≤ 0.30 µM, cytotoxicity IC_50_ > 38 µM, hemolysis HC_10_ > 38 µM) and **23c** (MIC MRSA ≤ 0.29 µM, cytotoxicity IC_50_ > 37 µM, hemolysis HC_10_ > 37 µM). Overall, the discovery of Gram-positive antibacterial activity for **23b** and **23c** with low to no cytotoxicity and hemolytic activity suggests a narrow structure–activity requirement of 7-methyl substitution and polyamine mid-chain length of 7 (PA3-7-3) or 8 (PA3-8-3) carbons for optimal activity.

The set of compounds were next evaluated for the ability to potentiate the activity of the antibiotic doxycycline towards the Gram-negative bacteria *P. aeruginosa* ATCC 27853 (Table 2). In these assays, doxycycline is present at a concentration of 2 µg/mL (4.5 µM), well below the observed MIC of 20 µg/mL (50 µM) towards this drug-resistant human pathogen.

**Table 2:** Doxycycline potentiation activity (MIC, µM) of analogues **2**–**8**, **17**–**23**.

Compound	Conc (µM) for Potentiation ^a^	Compound	Conc (µM) for Potentiation ^a^
**2**	>50 ^b^	**20a**	12.5
**3**	3.125 ^b^	**20b**	12.5
**4**	12.5 ^b^	**20c**	400
**5**	6.25 ^b^	**20d**	400
**6**	3.125 ^b^	**20e**	400
**7**	3.75 ^b^	**21a**	3.7
**8**	6.25 ^b^	**21b**	7.3
**17a**	12.5	**21c**	58
**17b**	100	**21d**	100
**17c**	50	**21e**	>200
**17d**	58	**22a**	100
**17e**	6.25	**22b**	100
**18a**	6.5	**22c**	200
**18b**	12.9	**22d**	400
**18c**	>200	**22e**	400
**18d**	25	**23a**	60
**18e**	24	**23b**	240
**19a**	7.3	**23c**	230
**19b**	114	**23d**	230
**19c**	25	**23e**	220
**19d**	>200		
**19e**	>200		

^a^ Concentration (µM) required to restore doxycycline activity at 2 µg/mL (4.5 µM) against *P. aeruginosa* ATCC 27853; ^b^ Data taken from Cadelis et al. [16].

Strong antibiotic enhancing activities were observed for the PA3-6-3 analogues **18a** (5-bromo, MIC 6.5 µM), **19a** (5-methoxy, MIC 7.3 µM) and **21a** (7-fluoro, MIC 3.7 µM), the 7-fluoro substituted PA3-7-3 analogue **21b** (MIC 7.3 µM), and the unsubstituted indolglyoxyl-PA3-12-3 analogue **17e** (MIC 6.25 µM).

A closer investigation of the ability of the 5-methoxy-indolglyoxyl-PA3-6-3 analogue **19a** to enhance the action of other antibiotics towards *P. aeruginosa* identified it to be capable of reactivating another tetracycline antibiotic minocycline (MIC 14.5 µM), was only a weak activator of chloramphenicol (MIC 58 µM) and could not restore the activity of erythromycin or nalidixic acid (Table 3).

**Table 3:** Antibiotic potentiating activity of **19a**.

Antibiotic	Concentration (µM) for Potentiation against *P. aeruginosa* ^a^
No antibiotic	>200
Minocycline	14.5
Erythromycin	>200
Chloramphenicol	58
Nalidixic acid	>200

All values presented as the mean (n = 3). ^a^ Concentration (µM) of compound **19a** required to restore antibiotic activity at 2 µg/mL concentration of antibiotic. *P. aeruginosa* ATCC 27853 against minocycline (MIC 70 µM), erythromycin (MIC >200 µM), chloramphenicol (MIC >200 µM) and nalidixic acid (MIC >200 µM).

The spectrum of antibiotic potentiating activity of the 7-fluoro analogue **21a** was also investigated, evaluating its ability to enhance other antibiotics against other Gram-negative bacteria (Table 4). The polyamine-conjugate was able to restore the action of doxycycline against *E. coli* (MIC 1.56 µM) and to a lesser degree against *Acinetobacter baumannii* (MIC 12.5 µM). Of the other combinations examined, **21a** was also found to weakly enhance the action of chloramphenicol and nalidixic acid towards *P. aeruginosa*. We have observed similar levels of drug-organism antibiotic enhancement for other examples of indolglyoxyl-polyamines [16].

## 3. Materials and Methods

### 3.1. Chemistry: General Remarks

Infrared spectra were recorded on a Perkin-Elmer spectrometer 100 Fourier-transform infrared spectrometer (Perkin-Elmer, MA, USA) equipped with a universal ATR accessory. Mass spectra were acquired on a Bruker micrOTOF Q II spectrometer. ^1^H and ^13^C NMR spectra were recorded at 298 K on a Bruker (Karlsruhe, Germany) AVANCE 400 spectrometer using standard pulse sequences. Proto-deutero solvent signals were used as internal references (DMSO-*d*_6_: δ_H_ 2.50, δ_C_ 39.52). For ^1^H NMR, the data are quoted as position (δ), relative integral, multiplicity (s = singlet, d = doublet, t = triplet, dt = doublet of triplet, tt = triplet of triplet, m = multiplet), coupling constant (*J*, Hz), and assignment to the atom. The ^13^C NMR data are quoted as position (δ), and assignment to the atom. Flash column chromatography was carried out using Davisil silica gel (40–60 μm) or Merck LiChroprep RP-8 (40–63 µm) (Merck Millipore, Darmstadt, Germany). Thin-layer chromatography was conducted on Merck DC Kieselgel 60 RP-18 F254S plates. All solvents used were of analytical grade or better and/or purified according to standard procedures. Chemical reagents used were purchased from standard chemical suppliers and used as purchased. Protected polyamines di-*tert*-butyl hexane-1,6-diylbis((3-aminopropyl)carbamate) (**9a**), di-*tert*-butyl heptane-1,7-diylbis((3-aminopropyl)carbamate) (**9b**), di-*tert*-butyl octane-1,8-diylbis((3-aminopropyl)carbamate) (**9c**), di-*tert*-butyl decane-1,10-diylbis((3-aminopropyl)carbamate) (**9d**), and di-*tert*-butyl dodecane-1,12-diylbis((3-aminopropyl)carbamate) (**9e**) [18,19,20,21], 2-(1*H*-indol-3-yl)-2-oxoacetyl chloride (**10**) [22], 2-(5-bromo-1*H*-indol-3-yl)-2-oxoacetyl chloride (**11**) [16], 2-(5-methoxy-1*H*-indol-3-yl)-2-oxoacetyl chloride (**12**) [23], 2-(5-methyl-1*H*-indol-3-yl)-2-oxoacetic acid (**13**) [24], 2-(7-fluoro-1*H*-indol-3-yl)-2-oxoacetyl chloride (**14**) [5], 2-(7-methoxy-1*H*-indol-3-yl)-2-oxoacetic acid (**15**) [25], and polyamine conjugates (**17c**/**17e**/**19c**/**19e**/**22c**/**22e**) [25] were synthesized using procedures from the literature.

#### 3.1.1. General Procedure A—Coupling of 3-Indolglyoxylyl Chlorides with Boc-Protected Polyamine

To a solution of 3-indolglyoxylyl chloride (2 equiv.) in DMF (1 mL) was added DIPEA (6 equiv.) and Boc-protected polyamine **9a**–**e** (1 equiv.) in DMF (1 mL). The reaction mixture was stirred for 48 h before solvent removal under reduced pressure. The crude product was purified using silica gel flash column chromatography (3% MeOH:CH_2_Cl_2_).

#### 3.1.2. General Procedure B—Boc Deprotection

A solution of *tert*-butyl-carbamate derivative in CH_2_Cl_2_ (2 mL) and TFA (0.2 mL) was stirred at room temperature under N_2_ for 2 h followed by solvent removal under reduced pressure. The crude product was purified using C_8_ reversed-phase flash column chromatography (0%–50% MeOH/H_2_O (+0.05% TFA)) to afford the product as a di-TFA salt.

#### 3.1.3. General procedure C—Coupling of Indole-Oxoacetic Acids with Boc-Protected Polyamine

To a solution of indole-oxoacetic acid (2 equiv.) and PyBOP (2 equiv.) in DMF (1 mL) was added DIPEA (3.5 equiv.) and Boc-protected polyamine **9a**–**e** (1 equiv.) in DMF (1 mL). The reaction mixture was stirred for 24 h under N_2_ at room temperature before the solvent was removed under reduced pressure. The crude product was purified using silica gel flash column chromatography (1–4% MeOH:CH_2_Cl_2_).

### 3.2. Synthesis of Compounds

#### 3.2.1. 2-(7-Methyl-1*H*-indol-3-yl)-2-oxoacetic Acid (**16**)

Oxalyl chloride (0.69 mL, 8.0 mmol) was added dropwise at 0 °C to 7-methyl-1*H*-indole (0.35 g, 2.7 mmol) in anhydrous diethyl ether (10 mL) and the solution stirred for 1.5 h. Saturated aq. NaHCO_3_ (10 mL) was then added, and the solution heated at reflux for 2 h. After cooling to room temperature, 10% aq. HCl was added to adjust the pH to 1. The resulting yellow precipitate was filtered, washed with cold diethyl ether (10 mL) and dried under vacuum, affording 2-(7-methyl-1*H*-indol-3-yl)-2-oxoacetic acid (**16**) as a yellow solid (0.52 g, 95%). The product was used in the next step without further purification. R*_f_* (MeOH:10% HCl, 3:1) 0.57; m.p. 206 °C (decomp); IR ν_max_ (ATR) 3320, 2944, 2832, 1625, 1448, 1112, 1028 cm^−1^; ^1^H NMR, (DMSO-*d_6_*, 400 MHz) δ 12.37 (1H, br s, NH-1), 8.37 (1H, d, *J* = 3.4 Hz, H-2), 8.01 (1H, d, *J* = 7.9 Hz, H-4), 7.16 (1H, t, *J* = 7.6 Hz, H-5), 7.08 (1H, d, *J* = 7.3 Hz, H-6), 2.51 (3H, s, Me), OH not observed; ^13^C NMR (DMSO-*d_6_*, 100 MHz) δ 180.9 (C-8), 165.3 (C-9), 137.5 (C-2), 136.1 (C-7a), 125.4 (C-3a), 124.3 (C-6), 122.9 (C-5), 122.1 (C-7), 118.7 (C-4), 112.7 (C-3), 16.7 (Me); (−)-HRESIMS [M-H]^–^ *m/z* 202.0514 (calcd for C_11_H_8_NO_3_, 202.0510).

#### 3.2.2. *N*^1^,*N*^6^-Bis(3-(2-(1*H*-indol-3-yl)-2-oxoacetamido)propyl)hexane-1,6-diaminium 2,2,2-trifluoroacetate (**17a**)

Using general procedure A, 2-(1*H*-indol-3-yl)-2-indoloxoacetyl chloride (**10**) (0.048 g, 0.24 mmol) was reacted with di-*tert*-butyl hexane-1,6-diylbis((3-aminopropyl)carbamate) (**9a**) (0.050 g, 0.12 mmol) and DIPEA (0.13 mL, 0.74 mmol) to afford di-*tert*-butyl hexane-1,6-diylbis((3-(2-(1*H*-indol-3-yl)-2-oxoacetamido)propyl)carbamate) as a yellow gum (0.045 g, 48%). Using general procedure B, a sub-sample of this product (0.040 g, 0.05 mmol) was reacted with TFA (0.2 mL) in CH_2_Cl_2_ (2 mL) to afford the di-TFA salt **17a** as a pale-yellow oil (0.018 g, 45%). R*_f_* (MeOH/10% HCl, 7:3) 0.63; IR (ATR) ν_max_ 3389, 2949, 2838, 1713, 1663, 1342, 1333, 1204, 1031 cm^−1^; ^1^H NMR (DMSO-*d*_6_, 400 MHz) δ 12.26 (2H, br s, NH-1), 8.89 (2H, t, *J* = 6.0 Hz, NH-10), 8.76 (2H, d, *J* = 3.3 Hz, H-2), 8.32 (4H, br s, NH_2_-14), 8.24–8.22 (2H, m, H-4), 7.55–7.53 (2H, m, H-7), 7.28–7.25 (4H, m, H-5, H-6), 3.35–3.26 (4H, obscured, H_2_-11), 2.96–2.86 (8H, m, H_2_-13, H_2_-15), 1.88–1.81 (4H, m, H_2_-12), 1.58–1.53 (4H, m, H_2_-16), 1.33–1.30 (4H, m, H_2_-17); ^13^C NMR (DMSO-*d*_6_, 100 MHz) δ 181.7 (C-8), 163.8 (C-9), 138.5 (C-2), 136.3 (C-7a), 126.2 (C-3a), 123.6 (C-6), 123.7 (C-5), 121.2 (C-4), 112.6 (C-7), 112.1 (C-3), 46.7 (C-15), 44.8 (C-13), 35.8 (C-11), 25.7, 25.5, 25.4 (C-12, C-16, C-17); (+)-HRESIMS [M+H]^+^ *m/z* 573.3190 (calcd for C_32_H_41_N_6_O_4_, 573.3184).

#### 3.2.3. *N*^1^,*N*^7^-Bis(3-(2-(1*H*-indol-3-yl)-2-oxoacetamido)propyl)heptane-1,7-diaminium 2,2,2-trifluoroacetate (**17b**)

Using general procedure A, 2-(1*H*-indol-3-yl)-2-oxoacetyl chloride (**10**) (0.072 g, 0.35 mmol) was reacted with di-*tert*-butyl heptane-1,7-diylbis((3-aminopropyl)carbamate) (**9b**) (0.078 g, 0.17 mmol) and DIPEA (0.18 mL, 1.03 mmol) to afford di-*tert*-butyl heptane-1,7-diylbis((3-(2-(1*H*-indol-3-yl)-2-oxoacetamido)propyl)carbamate) as a yellow oil (0.046 g, 33%). Using general procedure B, a sub-sample of this product (0.010 g, 0.013 mmol) was reacted with TFA (0.2 mL) in CH_2_Cl_2_ (2 mL) to afford the di-TFA salt **17b** as an orange oil (0.011 g, 100%). R*_f_* (MeOH/10% HCl, 7:3) 0.64; IR (ATR) ν_max_ 3269, 2865, 1677, 1627, 1495, 1438 cm^−1^; ^1^H NMR (DMSO-*d*_6_, 400 MHz) δ 12.36 (2H, s, NH-1), 8.87 (2H, t, *J* = 6.1 Hz, NH-10), 8.76 (2H, s, H-2), 8.65 (4H, br s, NH_2_-14), 8.24–8.22 (2H, m, H-4), 7.57–7.53 (2H, m, H-7), 7.29–7.23 (2H, m, H-5, H-6), 3.31 (4H, m, H_2_-11), 2.92–2.88 (8H, m, H_2_-13, H_2_-15), 1.88–1.85 (4H, m, H_2_-12), 1.57 (4H, br s, H_2_-16), 1.28–1.23 (6H, m, H_2_-17, H_2_-18); ^13^C NMR (DMSO-*d*_6_, 100 MHz) δ 181.8 (C-8), 163.8 (C-9), 138.5 (C-2), 136.3 (C-7a), 126.2 (C-3a), 123.5 (C-6), 122.6 (C-5), 121.3 (C-4), 112.6 (C-7), 112.1 (C-3), 46.7 (C-15), 44.7 (C-13), 35.8 (C-11), 28.0 (C-18), 25.8, 25.7, 25.4 (C-12, C-16, C-17); (+)-HRESIMS [M+H]^+^ *m/z* 587.3344 (calcd for C_33_H_43_N_6_O_4,_ 587.3340).

#### 3.2.4. *N*^1^,*N*^10^-Bis(3-(2-(1*H*-indol-3-yl)-2-oxoacetamido)propyl)decane-1,10-diaminium 2,2,2-trifluoroacetate (**17d**)

Using general procedure A, 2-(1*H*-indol-3-yl)-2-oxoacetyl chloride (**10**) (0.073 g, 0.36 mmol) was reacted with di-*tert*-butyl decane-1,10-diylbis((3-aminopropyl)carbamate) (**9d**) (0.084 g, 0.17 mmol) and DIPEA (0.19 mL, 1.1 mmol) to afford di-*tert*-butyl decane-1,10-diylbis((3-(2-(1*H*-indol-3-yl)-2-oxoacetamido)propyl)carbamate) as a dark yellow oil (0.056 g, 40%). Using general procedure B, a sub-sample of this product (0.016 g, 0.019 mmol) was reacted with TFA (0.2 mL) in CH_2_Cl_2_ (2 mL) to afford the di-TFA salt **17d** as a yellow oil (0.011 g, 66%). R*_f_* (MeOH/10% HCl, 7:3) 0.60; IR (ATR) ν_max_ 3410, 2844, 2677, 1630, 1494, 1441 cm^−1^; ^1^H NMR (DMSO-*d*_6_, 400 MHz) δ 12.29 (2H, s, NH-1), 8.89 (2H, t, *J* = 6.0 Hz, NH-10), 8.76 (2H, d, *J* = 2.6 Hz, H-2), 8.41 (4H, br s, NH_2_-14), 8.24–8.22 (2H, m, H-4), 7.55–7.53 (2H, m, H-7), 7.29–7.25 (4H, m, H-5, H-6), 3.30–3.27 (4H, obscured, H_2_-11), 2.98–2.84 (8H, m, H_2_-13, H_2_-15), 1.88–1.81 (4H, m, H_2_-12), 1.57–1.53 (4H, m, H_2_-16), 1.24 (12H, br s, H_2_-17, H_2_-18, H_2_-19); ^13^C NMR (DMSO-*d*_6_, 100 MHz) δ 181.7 (C-8), 163.7 (C-9), 138.5 (C-2), 136.2 (C-7a), 126.2 (C-3a), 123.5 (C-6), 122.6 (C-5), 121.2 (C-4), 112.6 (C-7), 112.1 (C-3), 46.8 (C-15), 44.7 (C-13), 35.8 (C-11), 28.7, 28.5 (C-18, C-19), 25.9, 25.7, 25.5 (C-12, C-16, C-17); (+)-HRESIMS [M+H]^+^ *m/z* 629.3804 (calcd for C_36_H_49_N_6_O_4_, 629.3810).

#### 3.2.5. *N*^1^,*N*^6^-Bis(3-(2-(5-bromo-1*H*-indol-3-yl)-2-oxoacetamido)propyl)hexane-1,6-diaminium 2,2,2-trifluoroacetate (**18a**)

Using general procedure A, 2-(5-bromo-1*H*-indol-3-yl)-2-indoloxoacetyl chloride (**11**) (0.067 g, 0.24 mmol) was reacted with di-*tert*-butyl hexane-1,6-diylbis((3-aminopropyl)carbamate) (**9a**) (0.050 g, 0.12 mmol) and DIPEA (0.13 mL, 0.74 mmol) to afford di-*tert*-butyl hexane-1,6-diylbis((3-(2-(5-bromo-1*H*-indol-3-yl)-2-oxoacetamido)propyl)carbamate) as a pale yellow gum (0.030 g, 27%). Using general procedure B, this product (0.030 g, 0.03 mmol) was reacted with TFA (0.2 mL) in CH_2_Cl_2_ (2 mL) to afford the di-TFA salt **18a** as a pale-yellow oil (0.006 g, 19%). R*_f_* (MeOH/10% HCl, 7:3) 0.38; IR (ATR) ν_max_ 3434, 1672, 1627, 1433, 1293, 1202, 1137, 1028, 721 cm^−1^; ^1^H NMR (DMSO-*d*_6_, 400 MHz) δ 12.48 (2H, br s, NH-1), 8.91 (2H, t, *J* = 6.0 Hz, NH-10), 8.80 (2H, s, H-2), 8.45 (4H, br s, NH_2_-14), 8.35 (2H, d, *J* = 2.0 Hz, H-4), 7.53 (2H, d, *J* = 8.5 Hz, H-7), 7.42 (2H, dd, *J* = 8.5, 2.0 Hz, H-6), 3.34–3.29 (4H, obscured, H_2_-11), 2.97–2.86 (8H, m, H_2_-13, H_2_-15), 1.85 (4H, tt, *J* = 8.5, 8.5 Hz, H_2_-12), 1.56 (4H, br s, H_2_-16), 1.31 (4H, br s, H_2_-17); ^13^C NMR (DMSO-*d*_6_, 100 MHz) δ 181.7 (C-8), 163.3 (C-9), 139.5 (C-2), 135.1 (C-7a), 128.0 (C-3a), 126.1 (C-6), 123.3 (C-4), 115.4 (C-5), 114.7 (C-7), 111.6 (C-3), 46.7 (C-15), 44.7 (C-13), 35.8 (C-11), 25.7, 25.5, 25.4 (C-12, C-16, C-17); (+)-HRESIMS [M+Na]^+^ *m/z* 751.1230 (calcd for C_32_H_38_^79^Br_2_N_6_NaO_4_, 751.1213), 753.1199 (calcd for C_32_H_38_^79^Br^81^BrN_6_NaO_4_, 753.1196), 755.1197 (calcd for C_32_H_38_^81^Br_2_N_6_NaO_4_, 755.1183).

#### 3.2.6. *N*^1^,*N*^7^-Bis(3-(2-(5-bromo-1*H*-indol-3-yl)-2-oxoacetamido)propyl)heptane-1,7-diaminium 2,2,2-trifluoroacetate (**18b**)

Using general procedure A, 2-(5-bromo-1*H*-indol-3-yl)-2-oxoacetyl chloride (**11**) (0.089 g, 0.31 mmol) was reacted with di-*tert*-butyl heptane-1,7-diylbis((3-aminopropyl)carbamate) (**9b**) (0.069 g, 0.15 mmol) and DIPEA (0.16 mL, 0.92 mmol) to afford di-*tert*-butyl heptane-1,7-diylbis((3-(2-(5-bromo-1*H*-indol-3-yl)-2-oxoacetamido)propyl)carbamate) as an orange oil (0.027 g, 17%). Using general procedure B, a sub-sample of this product (0.010 g, 0.010 mmol) was reacted with TFA (0.2 mL) in CH_2_Cl_2_ (2 mL) to afford the di-TFA salt **18b** as a yellow oil (0.009 g, 80%). R*_f_* (MeOH/10% HCl, 7:3) 0.35; IR (ATR) ν_max_ 3422, 2955, 2839, 1680, 1434 cm^−1^; ^1^H NMR (DMSO-*d*_6_, 400 MHz) δ 12.48 (2H, d, *J* = 2.8 Hz, NH-1), 8.91 (2H, t, *J* = 6.0 Hz, NH-10), 8.80 (2H, d, *J* = 2.8, H-2), 8.44 (4H, br s, NH_2_-14), 8.35 (2H, d, *J* = 2.0 Hz, H-4), 7.53 (2H, d, *J* = 8.8 Hz, H-7), 7.42 (2H, dd, *J* = 8.8, 2.0 Hz, H-6), 3.30 (4H, dt, *J* = 6.4, 6.4 Hz, H_2_-11), 2.93–2.89 (8H, m, H_2_-13, H_2_-15), 1.88–1.81 (4H, m, H_2_-12), 1.56 (4H, br s, H_2_-16), 1.29–1.23 (6H, m, H_2_-17, H_2_-18); ^13^C NMR (DMSO-*d*_6_, 100 MHz) δ 181.7 (C-8), 163.3 (C-9), 139.5 (C-2), 135.1 (C-7a), 128.0 (C-3a), 126.1 (C-6), 123.3 (C-4), 115.4 (C-5), 114.7 (C-7), 111.6 (C-3), 46.7 (C-15), 44.7 (C-13), 35.8 (C-11), 28.0 (C-18), 25.8, 25.7, 25.4 (C-12, C-16, C-17); (+)-HRESIMS [M+H]^+^ *m/z* 743.1570 (calcd for C_33_H_41_^79^Br_2_N_6_O_4_, 743.1551), 745.1555 (calcd for C_33_H_41_^79^Br^81^BrN_6_O_4_, 745.1533), 747.1544 (calcd for C_33_H_41_^81^Br_2_N_6_O_4_, 747.1521).

#### 3.2.7. *N*^1^,*N*^8^-Bis(3-(2-(5-bromo-1*H*-indol-3-yl)-2-oxoacetamido)propyl)octane-1,8-diaminium 2,2,2-trifluoroacetate (**18c**)

Using general procedure A, 2-(5-bromo-1*H*-indol-3-yl)-2-oxoacetyl chloride (**11**) (0.072 g, 0.25 mmol) was reacted with di-*tert*-butyl octane-1,8-diylbis((3-aminopropyl)carbamate) (**9c**) (0.055 g, 0.12 mmol) and DIPEA (0.12 mL, 0.66 mmol) to afford di-*tert*-butyl octane-1,8-diylbis((3-(2-(5-bromo-1*H*-indol-3-yl)-2-oxoacetamido)propyl)carbamate) as a brown oil (0.035 g, 30%). Using general procedure B, a sub-sample of this product (0.020 g, 0.021 mmol) was reacted with TFA (0.2 mL) in CH_2_Cl_2_ (2 mL) to afford the di-TFA salt **18c** as a brown oil (0.0048 g, 23%). R*_f_* (MeOH/10% HCl, 7:3) 0.32; IR (ATR) ν_max_ 3056, 2163, 1978, 1711, 1677, 1433, 1360, 1265, 1203, 1141, 1058, 738, 704 cm^−1^; ^1^H NMR (DMSO-*d*_6_, 400 MHz) δ 12.46 (2H, s, NH-1), 8.93 (2H, t, *J* = 6.1 Hz, NH-10), 8.82 (2H, br s, H-2), 8.37 (2H, d, *J* = 2.0 Hz, H-4), 8.36 (4H, br s, NH_2_-14), 7.55 (2H, d, *J* = 8.9 Hz, H-7), 7.44 (2H, dd, *J* = 8.6, 1.8 Hz, H-6), 3.30 (4H, obscured, H_2_-11), 2.94–2.89 (8H, m, H_2_-13, H_2_-15), 1.86–1.83 (4H, m, H_2_-12), 1.55–1.53 (4H, br s, H_2_-16), 1.26–1.23 (8H, m, H_2_-17, H_2_-18); ^13^C NMR (DMSO-*d*_6_, 100 MHz) δ 181.7 (C-8), 163.3 (C-9), 139.5 (C-2), 135.0 (C-7a), 128.0 (C-3a), 126.1 (C-6), 123.3 (C-4), 115.4 (C-5), 114.8 (C-7), 111.6 (C-3), 46.7 (C-15), 44.7 (C-13), 35.8 (C-11), 28.3 (C-18), 25.8, 25.7, 25.5 (C-12, C-16, C-17); (+)-HRESIMS [M+Na]^+^ *m/z* 779.1548 (calcd C_34_H_42_^79^Br_2_N_6_NaO_4_, 779.1526), 781.1513 (calcd C_34_H_42_^79^Br^81^BrN_6_NaO_4_, 781.1509), 783.1497 (calcd C_34_H_42_^81^Br_2_N_6_NaO_4_, 783.1497).

#### 3.2.8. *N*^1^,*N*^10^-Bis(3-(2-(5-bromo-1*H*-indol-3-yl)-2-oxoacetamido)propyl)decane-1,10-diaminium 2,2,2-trifluoroacetate (**18d**)

Using general procedure A, 2-(5-bromo-1*H*-indol-3-yl)-2-oxoacetyl chloride (**11**) (0.083 g, 0.29 mmol) was reacted with di-*tert*-butyl decane-1,10-diylbis((3-aminopropyl)carbamate) (**9d**) (0.068 g, 0.14 mmol) and DIPEA (0.15 mL, 0.86 mmol) to afford di-*tert*-butyl decane-1,10-diylbis((3-(2-(5-bromo-1*H*-indol-3-yl)-2-oxoacetamido)propyl)carbamate) as an orange oil (0.042 g, 30%). Using general procedure B, a sub-sample of this product (0.026 g, 0.026 mmol) was reacted with TFA (0.2 mL) in CH_2_Cl_2_ (2 mL) to afford the di-TFA salt **18d** as a brown oil (0.007 g, 34%). R*_f_* (MeOH/10% HCl, 7:3) 0.34; IR (ATR) ν_max_ 3023, 1676, 1438, 1203, 1030, 721 cm^−1^; ^1^H NMR (DMSO-*d*_6_, 400 MHz) δ 12.46 (2H, d, *J* = 2.1 Hz, NH-1), 8.91 (2H, t, *J* = 5.9 Hz, NH-10), 8.80 (2H, s, H-2), 8.35 (4H, br s, NH_2_-14), 8.35 (2H, d, *J* = 2.0 Hz, H-4), 7.53 (2H, d, *J* = 8.6 Hz, H-7), 7.42 (2H, dd, *J* = 8.5, 2.1 Hz, H-6), 3.30 (4H, dt, *J* = 6.5, 6.5 Hz, H_2_-11), 2.97–2.84 (8H, m, H_2_-13, H_2_-15), 1.84 (4H, tt, *J* = 7.6, 7.6 Hz, H_2_-12), 1.55 (4H, br s, H_2_-16), 1.24 (12H, br s, H_2_-17, H_2_-18, H_2_-19); ^13^C NMR (DMSO-*d*_6_, 100 MHz) δ 181.7 (C-8), 163.3 (C-9), 139.5 (C-2), 135.1 (C-7a), 128.0 (C-3a), 126.1 (C-6), 123.3 (C-4), 115.4 (C-5), 114.7 (C-7), 111.6 (C-3), 46.8 (C-15), 44.7 (C-13), 35.8 (C-11), 28.7, 28.5 (C-18, C-19), 25.9, 25.6, 25.5 (C-12, C-16, C-17); (+)-HRESIMS [M+H]^+^ *m/z* 785.2001 (calcd for C_36_H_47_^79^Br_2_N_6_O_4_, 785.2020), 787.1988 (calcd for C_36_H_47_^79^Br^81^BrN_6_O_4_, 787.2003), 789.1973 (calcd for C_36_H_47_^81^Br_2_N_6_O_4_, 789.1992).

#### 3.2.9. *N*^1^,*N*^12^-Bis(3-(2-(5-bromo-1*H*-indol-3-yl)-2-oxoacetamido)propyl)dodecane-1,12-diaminium 2,2,2-trifluoroacetate (**18e**)

Using general procedure A, 2-(5-bromo-1*H*-indol-3-yl)-2-oxoacetyl chloride (**11**) (0.081 g, 0.28 mmol) was reacted with di-*tert*-butyl dodecane-1,12-diylbis((3-aminopropyl)carbamate) (**9e**) (0.073 g, 0.14 mmol) and DIPEA (0.15 mL, 0.86 mmol) to afford di-*tert*-butyl dodecane-1,12-diylbis((3-(2-(5-bromo-1*H*-indol-3-yl)-2-oxoacetamido)propyl)carbamate) as a dark orange oil (0.047 g, 33%). Using general procedure B, a sub-sample of this product (0.014 g, 0.014 mmol) was reacted with TFA (0.2 mL) in CH_2_Cl_2_ (2 mL) to afford the di-TFA salt **18e** as a yellow oil (0.011 g, 76%). R*_f_* (MeOH/10% HCl, 7:3) 0.35; IR (ATR) ν_max_ 3402, 2981, 2036, 1681, 1654, 1385 cm^−1^; ^1^H NMR (DMSO-*d*_6_, 400 MHz) δ 12.50 (2H, d, *J* = 2.3 Hz, NH-1), 8.91 (2H, t, *J* = 6.0 Hz, NH-10), 8.80 (2H, d, *J* = 2.4, H-2), 8.44 (4H, br s, NH_2_-14), 8.35 (2H, d, *J* = 1.7 Hz, H-4), 7.53 (2H, d, *J* = 8.6 Hz, H-7), 7.41 (2H, dd, *J* = 8.6, 1.8 Hz, H-6), 3.30 (4H, dt, *J* = 6.3, 6.3 Hz, H_2_-11), 2.98–2.84 (8H, m, H_2_-13, H_2_-15), 1.86–1.82 (4H, m, H_2_-12), 1.55 (4H, br s, H_2_-16), 1.27–1.23 (16H, m, H_2_-17, H_2_-18, H_2_-19, H_2_-20); ^13^C NMR (DMSO-*d*_6_, 100 MHz) δ 181.7 (C-8), 163.4 (C-9), 139.5 (C-2), 135.1 (C-7a), 128.0 (C-3a), 126.1 (C-4), 123.3 (C-6), 115.4 (C-5), 114.7 (C-7), 111.6 (C-3), 46.8 (C-15), 44.7 (C-13), 35.8 (C-11), 28.9, 28.8, 28.5 (C-18, C-19, C-20), 25.9, 25.6, 25.5 (C-12, C-16, C-17); (+)-HRESIMS [M+H]^+^ *m/z* 813.2336 (calcd for C_38_H_51_^79^Br_2_N_6_O_4_, 813.2333), 815.2315 (calcd for C_38_H_51_^79^Br^81^BrN_6_O_4_, 815.2317), 817.2308 (calcd for C_38_H_51_^81^Br_2_N_6_O_4_, 817.2306).

#### 3.2.10. *N*^1^,*N*^6^-Bis(3-(2-(5-methoxy-1*H*-indol-3-yl)-2-oxoacetamido)propyl)hexane-1,6-diaminium 2,2,2-trifluoroacetate (**19a**)

Using general procedure A, 2-(5-methoxy-1*H*-indol-3-yl)-2-indoloxoacetyl chloride (**12**) (0.057 g, 0.24 mmol) was reacted with di-*tert*-butyl hexane-1,6-diylbis((3-aminopropyl)carbamate) (**9a**) (0.050 g, 0.12 mmol) and DIPEA (0.13 mL, 0.74 mmol) to afford di-*tert*-butyl hexane-1,6-diylbis((3-(2-(5-methoxy-1*H*-indol-3-yl)-2-oxoacetamido)propyl)carbamate) as an orange oil (0.025 g, 24%). Using general procedure B, a sub-sample of this product (0.018 g, 0.022 mmol) was reacted with TFA (0.2 mL) in CH_2_Cl_2_ (2 mL) to afford the di-TFA salt **19a** as a pale orange oil (0.016 g, 86%). R*_f_* (MeOH/10% HCl, 7:3) 0.74; IR (ATR) ν_max_ 3422, 1691, 1628, 1485, 1274, 1201, 1052, 1026, 1006, 823, 760, 734 cm^−1^; ^1^H NMR (DMSO-*d*_6_, 500 MHz) δ 12.18 (2H, br s, NH-1), 8.86 (2H, t, *J* = 5.8 Hz, NH-10), 8.69 (2H, d, *J* = 3.5 Hz, H-2), 8.44 (4H, br s, NH_2_-14), 7.74 (2H, d, *J* = 2.6 Hz, H-4), 7.44 (2H, d, *J* = 8.8 Hz, H-7), 6.91 (2H, dd, *J* = 8.8, 2.6 Hz, H-6), 3.79 (6H, s, OMe), 3.30 (4H, dt, *J* = 6.8, 6.8 Hz, H_2_-11), 2.97–2.86 (8H, br m, H_2_-13, H_2_-15), 1.84 (4H, br s, H_2_-12), 1.56 (4H, br s, H_2_-16), 1.31 (4H, br s, H_2_-17); ^13^C NMR (DMSO-*d*_6_, 125 MHz) δ 181.5 (C-8), 163.8 (C-9), 156.0 (C-5), 138.4 (C-2), 131.0 (C-7a), 127.2 (C-3a), 113.3 (C-7), 112.8 (C-6), 112.0 (C-3), 103.5 (C-4), 55.3 (OMe), 46.7 (C-15), 44.7 (C-13), 35.8 (C-11), 25.7, 25.5, 25.4 (C-12, C-16, C-17); (+)-HRESIMS [M+H]^+^ *m/z* 633.3396 (calcd for C_34_H_45_N_6_O_6_, 633.3395).

#### 3.2.11. *N*^1^,*N*^7^-Bis(3-(2-(5-methoxy-1*H*-indol-3-yl)-2-oxoacetamido)propyl)heptane-1,7-diaminium 2,2,2-trifluoroacetate (**19b**)

Using general procedure A, 2-(5-methoxy-1*H*-indol-3-yl)-2-oxoacetyl chloride (**12**) (0.079 g, 0.33 mmol) was reacted with di-*tert*-butyl heptane-1,7-diylbis((3-aminopropyl)carbamate) (**9b**) (0.070 g, 0.15 mmol) and DIPEA (0.16 mL, 0.92 mmol) to afford di-*tert*-butyl heptane-1,7-diylbis((3-(2-(5-methoxy-1*H*-indol-3-yl)-2-oxoacetamido)propyl)carbamate) as a yellow oil (0.004 g, 33%). Using general procedure B, a sub-sample of this product (0.017 g, 0.02 mmol) was reacted with TFA (0.2 mL) in CH_2_Cl_2_ (2 mL) to afford the di-TFA salt **19b** as a yellow oil (0.014 g, 80%). R*_f_* (MeOH/10% HCl, 7:3) 0.72; IR (ATR) ν_max_ 3318, 2981, 1678, 1621, 1486, 1438 cm^−1^; ^1^H NMR (DMSO-*d*_6_, 400 MHz) δ 12.22 (2H, d, *J* = 2.2 Hz, NH-1), 8.86 (2H, t, *J* = 6.0 Hz, NH-10), 8.69 (2H, d, *J* = 3.3 Hz, H-2), 8.51 (4H, br s, NH_2_-14), 7.75 (2H, d, *J* = 2.5 Hz, H-4), 7.44 (2H, d, *J* = 8.8 Hz, H-7), 6.90 (2H, dd, *J* = 8.8, 2.5 Hz, H-6), 3.79 (6H, s, OMe), 3.30 (4H, dt, *J* = 6.4, 6.4 Hz, H_2_-11), 2.98–2.89 (8H, m, H_2_-13, H_2_-15), 1.89–1.88 (4H, m, H_2_-12), 1.56 (4H, br s, H_2_-16), 1.29 (6H, br s, H_2_-17, H_2_-18); ^13^C NMR (DMSO-*d*_6_, 100 MHz) δ 181.6 (C-8), 163.9 (C-9), 156.0 (C-5), 138.4 (C-2), 131.0 (C-7a), 127.2 (C-3a), 113.3 (C-7), 112.8 (C-6), 112.0 (C-3), 103.5 (C-4), 55.3 (OMe), 46.7 (C-15), 44.7 (C-13), 35.8 (C-11), 28.0 (C-18), 25.8, 25.7, 25.4 (C-12, C-16, C-17); (+)-HRESIMS [M+H]^+^ *m/z* 647.3554 (calcd for C_35_H_47_N_6_O_6_, 647.3552).

#### 3.2.12. *N*^1^,*N*^10^-Bis(3-(2-(5-methoxy-1*H*-indol-3-yl)-2-oxoacetamido)propyl)decane-1,10-diaminium 2,2,2-trifluoroacetate (**19d**)

Using general procedure A, 2-(5-methoxy-1*H*-indol-3-yl)-2-oxoacetyl chloride (**12**) (0.081 g, 0.34 mmol) was reacted with di-*tert*-butyl decane-1,10-diylbis((3-aminopropyl)carbamate) (**9d**) (0.076 g, 0.16 mmol) and DIPEA (0.17 mL, 0.97 mmol) to afford di-*tert*-butyl decane-1,10-diylbis((3-(2-(5-methoxy-1*H*-indol-3-yl)-2-oxoacetamido)propyl)carbamate) as a yellow oil (0.033 g, 23%). Using general procedure B, a sub-sample of this product (0.016 g, 0.018 mmol) was reacted with TFA (0.2 mL) in CH_2_Cl_2_ (2 mL) to afford the di-TFA salt **19d** as a dark orange oil (0.014 g, 85%). R*_f_* (MeOH/10% HCl, 7:3) 0.76; IR (ATR) ν_max_ 3364, 2946, 2833, 1579, 1419 cm^−1^; ^1^H NMR (DMSO-*d*_6_, 400 MHz) δ 12.24 (2H, s, NH-1), 8.85 (2H, t, *J* = 6.0 Hz, NH-10), 8.68 (2H, d, *J* = 3.0 Hz, H-2), 8.54 (4H, br s, NH_2_-14), 7.75 (2H, d, *J* = 1.9 Hz, H-4), 7.44 (2H, d, *J* = 8.7 Hz, H-7), 6.90 (2H, dd, *J* = 8.8, 1.9 Hz, H-6), 3.79 (6H, s, OMe), 3.29 (4H, dt, *J* = 6.2, 6.2 Hz, H_2_-11), 2.97–2.85 (8H, br m, H_2_-13, H_2_-15), 1.86–1.81 (4H, m, H_2_-12), 1.55 (4H, br m, H_2_-16), 1.24 (12H, br s, H_2_-17, H_2_-18, H_2_-19); ^13^C NMR (DMSO-*d*_6_, 100 MHz) δ 181.6 (C-8), 163.9 (C-9), 156.0 (C-5), 138.4 (C-2), 131.0 (C-7a), 127.2 (C-3a), 113.3 (C-7), 112.8 (C-6), 112.0 (C-3), 103.5 (C-4), 55.3 (OMe), 46.8 (C-15), 44.7 (C-13), 35.8 (C-11), 28.7, 28.5 (C-18, C-19), 25.9, 25.7, 25.5 (C-12, C-16, C-17); (+)-HRESIMS [M+H]^+^ *m/z* 689.4040 (calcd for C_38_H_53_N_6_O_6_, 689.4021).

#### 3.2.13. *N*^1^,*N*^6^-Bis(3-(2-(5-methyl-1*H*-indol-3-yl)-2-oxoacetamido)propyl)hexane-1,6-diaminium 2,2,2-trifluoroacetate (**20a**)

Using general procedure C, 2-(5-methyl -1*H*-indol-3-yl)-2-oxoacetic acid (**13**) (0.070 g, 0.34 mmol) was reacted with di-*tert*-butyl hexane-1,6-diylbis((3-aminopropyl) carbamate) (**9a**) (0.072 g, 0.17 mmol), PyBOP (0.178 g, 0.34 mmol) and DIPEA (0.09 mL, 0.52 mmol). Purification by column chromatography afforded di-*tert*-butyl hexane-1,6-diylbis((3-(2-(5-methyl-1*H*-indol-3-yl)-2-oxoacetamido) propyl) carbamate) as a yellow oil (0.054 g, 39%). Using general procedure B, a sub-sample of this product (0.025 g, 0.031 mmol) was reacted with TFA (0.2 mL) in CH_2_Cl_2_ (2 mL) to afford the di-TFA salt **20a** as a brown gum (0.021 g, 81%). R*_f_* (MeOH/10% HCl, 3:1) 0.59; IR (ATR) ν_max_ 3325, 2945, 1678, 1448, 1113, 1021 cm^−1^; ^1^H NMR, (DMSO-*d*_6_, 400 MHz) δ 12.20 (2H, d, *J* = 2.8 Hz, NH-1), 8.85 (2H, t, *J* = 6.1 Hz, H-10), 8.69 (2H, d, *J* = 3.3 Hz, H-2), 8.49 (4H, br s, H-14), 8.03 (2H, s, H-4), 7.41 (2H, d, *J* = 8.2 Hz, H-7), 7.09 (2H, dd, *J* = 8.4, 1.4 Hz, H-6), 3.30 (4H, dt, *J* = 6.6, 6.6 Hz, H_2_-11), 2.97–2.85 (8H, m, H_2_-13, H_2_-15), 2.42 (6H, s, Me), 1.88–1.81 (4H, m, H_2_-12), 1.56 (4H, br s, H_2_-16), 1.30 (4H, br s, H_2_-17); ^13^C NMR (DMSO-*d*_6_, 100 MHz) δ 181.7 (C-8), 164.0 (C-9), 138.5 (C-2), 134.6 (C-7a), 131.6 (C-5), 126.5 (C-3a), 125.0 (C-6), 121.1 (C-4), 112.3 (C-7), 111.8 (C-3), 46.7 (C-15), 44.8 (C-13), 35.8 (C-11), 25.8, 25.5, 25.4 (C-12, C-16, C-17), 21.4 (Me); (+)-HRESIMS [M+H]^+^ *m/z* 601.3511 (calcd for C_34_H_45_N_6_O_4_, 601.3497).

#### 3.2.14. *N*^1^,*N*^7^-Bis(3-(2-(5-methyl-1*H*-indol-3-yl)-2-oxoacetamido)propyl)heptane-1,7-diaminium 2,2,2-trifluoroacetate (**20b**)

Using general procedure C, 2-(5-methyl-1*H*-indol-3-yl)-2-oxoacetic acid (**13**) (0.080 g, 0.39 mmol) was reacted with di-*tert*-butyl heptane-1,7-diylbis((3-aminopropyl) carbamate) (**9b**) (0.085 g, 0.19 mmol), PyBOP (0.204 g, 0.39 mmol) and DIPEA (0.1 mL, 0.57 mmol). Purification by column chromatography afforded di-*tert*-butyl heptane-1,7-diylbis((3-(2-(5-methyl-1*H*-indol-3-yl)-2-oxoacetamido) propyl) carbamate) as a yellow oil (0.079 g, 51%). Using general procedure B, a sub-sample of this product (0.045 g, 0.055 mmol) was reacted with TFA (0.2 mL) in CH_2_Cl_2_ (2 mL) to afford the di-TFA salt **20b** as a brown gum (0.024 g, 52%). R*_f_* (MeOH/10% HCl, 3:1) 0.53; IR (ATR) ν_max_ 3306, 2943, 1653, 1448, 1118, 1022, 739 cm^−1^; ^1^H NMR (DMSO-*d*_6_, 400 MHz) δ 12.24 (2H, d, *J* = 2.7 Hz, NH-1), 8.85 (2H, t, *J* = 6.2 Hz, NH-10), 8.70 (2H, d, *J* = 3.5 Hz, H-2), 8.55 (4H, br s, NH-14), 8.04 (2H, br s, H-4), 7.42 (2H, d, *J* = 8.1 Hz, H-7), 7.09 (2H, dd, *J* = 8.43 and 1.4, H-6), 3.29 (4H, dt, *J* = 6.5, 6.5 Hz, H_2_-11), 2.97–2.85 (8H, m, H_2_-13, H_2_-15), 2.42 (6H, s, Me), 1.89–1.82 (4H, m, H_2_-12), 1.60–1.52 (4H, br m, H_2_-16), 1.28 (6H, br s, H_2_-17, H_2_-18); ^13^C NMR (DMSO-*d*_6_, 100 MHz) δ 181.7 (C-8), 163.9 (C-9), 138.5 (C-2), 134.6 (C-7a), 131.6 (C-5), 126.5 (C-3a), 124.9 (C-6), 121.1 (C-4), 112.3 (C-7), 111.8 (C-3), 46.7 (C-15), 44.7 (C-13), 35.8 (C-11), 28.0 (C-18), 25.8, 25.7, 25.4, (C-12, C-16, C-17), 21.4 (Me); (+)-HRESIMS [M+H]^+^ *m/z* 615.3664 (calcd for C_35_H_47_N_6_O_4_, 615.3653).

#### 3.2.15. *N*^1^,*N*^8^-Bis(3-(2-(5-methyl-1*H*-indol-3-yl)-2-oxoacetamido)propyl)octane-1,8-diaminium 2,2,2-trifluoroacetate (**20c**)

Using general procedure C, 2-(5-methyl -1*H*-indol-3-yl)-2-oxoacetic acid (**13**) (0.080 g, 0.39 mmol) was reacted with di-*tert*-butyl octane-1,8-diylbis((3-aminopropyl)carbamate) (**9c**) (0.088 g, 0.19 mmol), PyBOP (0.204 g, 0.39 mmol) and DIPEA (0.1 mL, 0.57 mmol). Purification by column chromatography afforded di-*tert*-butyl octane-1,8-diylbis((3-(2-(5-methyl-1*H*-indol-3-yl)-2-oxoacetamido)propyl)carbamate) as a yellow oil (0.054 g, 34%). Using general procedure B, a sub-sample of this product (0.030 g, 0.036 mmol) was reacted with TFA (0.2 mL) in CH_2_Cl_2_ (2 mL) to afford the di-TFA salt **20c** as a brown gum (0.030 g, 97%). R*_f_* (MeOH/10% HCl, 3:1) 0.50; IR (ATR) ν_max_ 3307, 2943, 1676, 1448, 1116, 1022, 713 cm^−1^; ^1^H NMR, (DMSO-*d*_6_, 400 MHz) δ 12.25 (2H, d, *J* = 3.0 Hz, NH-1), 8.85 (2H, t, *J* = 6.1 Hz, NH-10), 8.70 (2H, d, *J* = 3.2 Hz, H-2), 8.55 (4H, br s, NH-14), 8.04 (2H, br s, H-4), 7.42 (2H, d, *J* = 8.1 Hz, H-7), 7.09 (2H, dd, *J* = 8.3, 1.5 Hz, H-6), 3.29 (4H, dt, *J* = 6.5, 6.5 Hz, H_2_-11), 2.94–2.85 (8H, br s, H_2_-13, H_2_-15), 2.42 (6H, s, Me), 1.89–1.82 (4H, br s, H_2_-12), 1.56 (4H, br s, H_2_-16), 1.26 (6H, br s, H_2_-17, H_2_-18); ^13^C NMR (DMSO-*d*_6_, 100 MHz) δ 181.7 (C-8), 163.9 (C-9), 138.4 (C-2), 134.6 (C-7a), 131.5 (C-5), 126.5 (C-3a), 124.9 (C-6), 121.1 (C-4), 112.3 (C-7), 111.8 (C-3), 46.8 (C-15), 44.7 (C-13), 35.8 (C-11), 28.3 (C-18), 25.8, 25.7, 25.5 (C-12, C-16, C-17), 21.4 (Me); (+)-HRESIMS [M+H]^+^ *m/z* 629.3818 (calcd for C_36_H_49_N_6_O_4_, 629.3810).

#### 3.2.16. *N*^1^,*N*^10^-Bis(3-(2-(5-methyl-1*H*-indol-3-yl)-2-oxoacetamido)propyl)decane-1,10-diaminium 2,2,2-trifluoroacetate (**20d**)

Using general procedure C, 2-(5-methyl -1*H*-indol-3-yl)-2-oxoacetic acid (**13**) (0.070 g, 0.34 mmol) was reacted with di-*tert*-butyl decane-1,10-diylbis((3-aminopropyl) carbamate) (**9d**) (0.081 g, 0.17 mmol), PyBOP (0.179 g, 0.34 mmol) and DIPEA (0.09 mL, 0.50 mmol). Purification by column chromatography afforded di-*tert*-butyl decane-1,10-diylbis((3-(2-(5-methyl-1*H*-indol-3-yl)-2-oxoacetamido)propyl)carbamate) as a yellow oil (0.087 g, 60%). Using general procedure B, a sub-sample of this product (0.043 g, 0.050 mmol) was reacted with TFA (0.2 mL) in CH_2_Cl_2_ (2 mL) to afford the di-TFA salt **20d** as a white gum (0.044 g, 99%). R*_f_* (MeOH/10% HCl, 3:1) 0.44; IR (ATR) ν_max_ 3307, 2944, 1678, 1449, 1115, 1021 cm^−1^; ^1^H NMR, (DMSO-*d*_6_, 400 MHz) δ 12.24 (2H, d, *J* = 3.0 Hz, NH-1), 8.85 (2H, t, *J* = 6.1 Hz, NH-10), 8.70 (2H, d, *J* = 3.4 Hz, H-2), 8.52 (4H, br s, NH-14), 8.04 (2H, br s, H-4), 7.42 (2H, d, *J* = 8.2 Hz, H-7), 7.09 (2H, dd, *J* = 8.3, 1.5 Hz, H-6), 3.30 (4H, dt, *J* = 6.5, 6.5 Hz, H_2_-11), 2.97–2.85 (8H, br m, H_2_-13, H_2_-15), 2.42 (6H, s, Me), 1.89–1.82 (4H, m, H_2_-12), 1.57–1.52 (4H, m, H_2_-16), 1.24 (12H, br s, H_2_-17, H_2_-18, H_2_-19); ^13^C NMR (DMSO-*d*_6_, 100 MHz) δ 181.7 (C-8), 163.9 (C-9), 138.5 (C-2), 134.6 (C-7a), 131.5 (C-5), 126.6 (C-3a), 124.9 (C-6), 121.1 (C-4), 112.3 (C-7), 111.8 (C-3), 46.8 (C-15), 44.7 (C-13), 35.8 (C-11), 28.7, 28.5 (C-18, C-19), 25.9, 25.7, 25.5 (C-12, C-16, C-17), 21.4 (Me), (+)-HRESIMS [M+H]^+^ *m/z* 657.4125 (calcd for C_38_H_53_N_6_O_4_, 657.4123).

#### 3.2.17. *N*^1^,*N*^12^-Bis(3-(2-(5-methyl-1*H*-indol-3-yl)-2-oxoacetamido)propyl)dodecane-1,12-diaminium 2,2,2-trifluoroacetate (**20e**)

Using general procedure C, 2-(5-methyl-1*H*-indol-3-yl)-2-oxoacetic acid (**13**) (0.070 g, 0.34 mmol) was reacted with di-*tert*-butyl dodecane-1,12-diylbis((3-aminopropyl)carbamate) (**9e**) (0.086 g, 0.17 mmol), PyBOP (0.179 g, 0.34 mmol) and DIPEA (0.09 mL, 0.50 mmol). Purification by column chromatography afforded di-*tert*-butyl dodecane-1,12-diylbis((3-(2-(5-methyl-1*H*-indol-3-yl)-2-oxoacetamido)propyl)carbamate) as a yellow oil (0.092 g, 62%). Using general procedure B, a sub-sample of this product (0.045 g, 0.051 mmol) was reacted with TFA (0.2 mL) in CH_2_Cl_2_ (2 mL) to afford the di-TFA salt **20e** as a white gum (0.039 g, 84%). R*_f_* (MeOH/10% HCl, 3:1) 0.41; IR (ATR) ν_max_ 3307, 2944, 1678, 1452, 1113, 740 cm^−1^; ^1^H NMR (DMSO-*d*_6_, 400 MHz) δ 12.24 (2H, d, *J* = 2.9 Hz, NH-1), 8.85 (2H, t, *J* = 6.3 Hz, NH-10), 8.70 (2H, d, *J* = 3.3 Hz, H-2), 8.51 (4H, br s, NH-14), 8.04 (2H, br s, H-4), 7.42 (2H, d, *J* = 8.3 Hz, H-7), 7.09 (2H, dd, *J* = 8.4, 1.5 Hz, H-6), 3.29 (4H, dt, *J* = 6.4, 6.4 Hz, H_2_-11), 2.97–2.85 (8H, br m, H_2_-13, H_2_-15), 2.42 (6H, s, Me), 1.89–1.82 (4H, m, H_2_-12), 1.57–1.52 (4H, m, H_2_-16), 1.31–1.22 (16H, br m, H_2_-17, H_2_-18, H_2_-19, H_2_-20); ^13^C NMR (DMSO-*d*_6_, 100 MHz) δ 181.7 (C-8), 163.9 (C-9), 138.5 (C-2), 134.6 (C-7a), 131.5 (C-5), 126.6 (C-3a), 124.9 (C-6), 121.1 (C-4), 112.3 (C-7), 111.8 (C-3), 46.8 (C-15), 44.7 (C-13), 35.8 (C-11), 29.0, 28.9, 28.6 (C-18, C-19, C-20), 25.9, 25.7, 25.5 (C-12, C-16, C-17), 21.4 (Me); (+)-HRESIMS [M+H]^+^ *m/z* 685.4453 (calcd for C_40_H_57_N_6_O_4_, 685.4436).

#### 3.2.18. *N*^1^,*N*^6^-Bis(3-(2-(7-fluoro-1*H*-indol-3-yl)-2-oxoacetamido)propyl)hexane-1,6-diaminium 2,2,2-trifluoroacetate (**21a**)

Using general procedure A, 2-(7-fluoro-1*H*-indol-3-yl)-2-indoloxoacetyl chloride (**14**) (0.053 g, 0.24 mmol) was reacted with di-*tert*-butyl hexane-1,6-diylbis((3-aminopropyl)carbamate) (**9a**) (0.050 g, 0.12 mmol) and DIPEA (0.13 mL, 0.74 mmol) to afford di-*tert*-butyl hexane-1,6-diylbis((3-(2-(7-fluoro-1*H*-indol-3-yl)-2-oxoacetamido)propyl)carbamate) as a yellow gum (0.016 g, 16%). Using general procedure B, this product (0.016 g, 0.02 mmol) was reacted with TFA (0.2 mL) in CH_2_Cl_2_ (2 mL) to afford the di-TFA salt **21a** as a pale yellow oil (0.006 g, 35%). R*_f_* (MeOH/10% HCl, 7:3) 0.76; IR (ATR) ν_max_ 3434, 1672, 1627, 1433, 1293, 1202, 1137, 1028, 721 cm^−1^; ^1^H NMR (DMSO-*d*_6_, 500 MHz) δ 12.88 (2H, br s, NH-1), 8.95 (2H, t, *J* = 5.6 Hz, NH-10), 8.77 (2H, s, H-2), 8.42 (4H, br s, NH_2_-14), 8.04 (2H, d, *J* = 8.0 Hz, H-4), 7.25 (2H, ddd, *J* = 8.0, 8.0, 5.0 Hz, H-5), 7.14 (2H, dd, *J* = 11.2, 8.0 Hz, H-6), 3.38–3.29 (4H, m, H_2_-11), 2.97–2.91 (8H, m, H_2_-13, H_2_-15), 1.85 (4H, tt, *J* = 7.3, 7.3 Hz, H_2_-12), 1.55 (4H, br s, H_2_-16), 1.31 (4H, br s, H_2_-17); ^13^C NMR (DMSO-*d*_6_, 125 MHz) δ 181.9 (C-8), 163.4 (C-9), 149.2 (d, ^1^*J*_CF_ = 245.4 Hz, C-7), 138.8 (C-2), 129.8 (d, ^3^*J*_CF_ = 4.5 Hz, C-3a), 124.0 (d, ^2^*J*_CF_ = 13.4 Hz, C-7a), 123.4 (d, ^3^*J*_CF_ = 5.9 Hz, C-5), 117.4 (d, ^4^*J*_CF_ = 2.7 Hz, C-4), 112.8 (C-3), 108.7 (d, ^2^*J*_CF_ = 15.9 Hz, C-6), 46.7 (C-15), 44.7 (C-13), 35.9 (C-11), 25.7, 25.5, 25.4 (C-12, C-16, C-17); (+)-HRESIMS [M+H]^+^ *m/z* 609.2987 (calcd for C_32_H_39_F_2_N_6_O_4_, 609.2995).

#### 3.2.19. *N*^1^,*N*^7^-Bis(3-(2-(7-fluoro-1*H*-indol-3-yl)-2-oxoacetamido)propyl)heptane-1,7-diaminium 2,2,2-trifluoroacetate (**21b**)

Using general procedure A, 2-(7-fluoro-1*H*-indol-3-yl)-2-oxoacetyl chloride (**14**) (0.080 g, 0.36 mmol) was reacted with di-*tert*-butyl heptane-1,7-diylbis((3-aminopropyl)carbamate) (**9b**) (0.079 g, 0.18 mmol) and DIPEA (0.19 mL, 1.1 mmol) to afford di-*tert*-butyl heptane-1,7-diylbis((3-(2-(7-fluoro-1*H*-indol-3-yl)-2-oxoacetamido)propyl)carbamate) as a dark yellow oil (0.058 g, 39%). Using general procedure B, a sub-sample of this product (0.013 g, 0.016 mmol) was reacted with TFA (0.2 mL) in CH_2_Cl_2_ (2 mL) to afford the di-TFA salt **21b** as an orange oil (0.013 g, 96%). R*_f_* (MeOH/10% HCl, 7:3) 0.75; IR (ATR) ν_max_ 3401, 2930, 1675, 1635, 1458 cm^−1^; ^1^H NMR (DMSO-*d*_6_, 400 MHz) δ 12.91 (2H, br d, *J* = 2.1 Hz, NH-1), 8.94 (2H, t, J = 6.1 Hz, NH-10), 8.77 (2H, d, J = 3.0 Hz, H-2), 8.51 (4H, br s, NH_2_-14), 8.04 (2H, d, *J* = 7.9 Hz, H-4), 7.25 (2H, ddd, *J* = 8.1, 8.1, 5.0 Hz, H-5), 7.31 (2H, dd, *J* = 11.3, 7.3 Hz, H-6), 3.30 (4H, dt, *J* = 6.5, 6.5 Hz, H_2_-11), 2.98–2.86 (8H, br m, H_2_-13, H_2_-15), 1.89–1.82 (4H, br m, H_2_-12), 1.56 (4H, br s, H_2_-16), 1.31–1.26 (6H, m, H_2_-17, H_2_-18); ^13^C NMR (DMSO-*d*_6_, 100 MHz) δ 181.9 (C-8), 163.4 (C-9), 149.2 (d, ^1^*J*_CF_ = 245 Hz, C-7), 138.8 (C-2), 129.9 (d, ^3^*J*_CF_ = 4.4 Hz, C-3a), 123.4 (d, ^3^*J*_CF_ = 6.0 Hz, C-5), 124.0 (d, ^2^*J*_CF_ = 13.2 Hz, C-7a), 117.4 (br s, C-4), 112.8 (C-3), 108.6 (d, ^2^*J*_CF_ = 15.5 Hz, C-6), 46.7 (C-15), 44.7 (C-13), 35.9 (C-11), 28.0 (C-18), 25.8, 25.7, 25.4 (C-12, C-16, C-17); (+)-HRESIMS [M+H]^+^ *m/z* 623.3161 (calcd for C_33_H_41_F_2_N_6_O_4_, 623.3152).

#### 3.2.20. *N*^1^,*N*^8^-Bis(3-(2-(7-fluoro-1*H*-indol-3-yl)-2-oxoacetamido)propyl)octane-1,8-diaminium 2,2,2-trifluoroacetate (**21c**)

Using general procedure A, 2-(7-fluoro-1*H*-indol-3-yl)-2-oxoacetyl chloride (**14**) (0.063 g, 0.28 mmol) was reacted with di-*tert*-butyl octane-1,8-diylbis((3-aminopropyl)carbamate) (**9c**) (0.056 g, 0.12 mmol) and DIPEA (0.13 mL, 0.75 mmol) to afford di-*tert*-butyl octane-1,8-diylbis((3-(2-(7-fluoro-1*H*-indol-3-yl)-2-oxoacetamido)propyl)carbamate) as a brown oil (0.064 g, 56%). Using general procedure B, a sub-sample of this product (0.034 g, 0.041 mmol) was reacted with TFA (0.2 mL) in CH_2_Cl_2_ (2 mL) to afford the di-TFA salt **21c** as a brown oil (0.030 g, 85%). R*_f_* (MeOH/10% HCl, 7:3) 0.70; IR (ATR) ν_max_ 3430, 1689, 1656, 1050, 1023, 1002, 930, 823, 760 cm^−1^; ^1^H NMR (DMSO-*d*_6_, 400 MHz) δ 12.89 (2H, d, *J* = 2.8 Hz, NH-1), 8.94 (2H, t, *J* = 6.1 Hz, NH-10), 8.77 (2H, d, *J* = 3.2 Hz, H-2), 8.46 (4H, br s, NH_2_-14), 8.04 (2H, d, *J* = 7.8 Hz, H-4), 7.25 (2H, ddd, *J* = 8.0, 8.0, 4.7 Hz, H-5), 7.14 (2H, ddd, *J* = 11.0, 7.9, 1.0 Hz, H-6), 3.30 (4H, dt, *J* = 6.4, 6.4 Hz, H_2_-11), 2.98–2.84 (4H, br m, H_2_-13, H_2_-15), 1.85 (4H, tt, *J* = 6.5, 6.5 Hz, H_2_-12), 1.55 (4H, tt, *J* = 6.8, 6.8 Hz, H_2_-16), 1.26 (8H, br s, H_2_-17, H_2_-18); ^13^C NMR (DMSO-*d*_6_, 100 MHz) δ 181.9 (C-8), 163.4 (C-9), 149.2 (^1^*J*_CF_ = 245 Hz, C-7), 138.8 (C-2), 129.8 (d, ^3^*J*_CF_ = 4.6 Hz, C-3a), 124.0 (d, ^2^*J*_CF_ = 13.3 Hz, C-7a), 123.4 (d, ^3^*J*_CF_ = 5.3 Hz, C-5), 117.4 (d, ^3^*J*_CF_ = 3.5 Hz, C-4), 112.8 (C-3), 108.7 (d, ^2^*J*_CF_ = 16.0 Hz, C-6), 46.7 (C-15), 44.7 (C-13), 35.8 (C-11), 28.3 (C-18), 25.8, 25.6, 25.5 (C-12, C-16, C-17); (+)-HRESIMS [M+H]^+^ *m/z* 637.3313 (calcd C_34_H_43_F_2_N_6_O_4_, 637.3308).

#### 3.2.21. *N*^1^,*N*^10^-Bis(3-(2-(7-fluoro-1*H*-indol-3-yl)-2-oxoacetamido)propyl)decane-1,10-diaminium 2,2,2-trifluoroacetate (**21d**)

Using general procedure A, 2-(7-fluoro-1*H*-indol-3-yl)-2-oxoacetyl chloride (**14**) (0.080 g, 0.35 mmol) was reacted with di-*tert*-butyl decane-1,10-diylbis((3-aminopropyl)carbamate) (**9d**) (0.084 g, 0.17 mmol) and DIPEA (0.19 mL, 1.1 mmol) to afford di-*tert*-butyl decane-1,10-diylbis((3-(2-(7-fluoro-1*H*-indol-3-yl)-2-oxoacetamido)propyl)carbamate) as a dark yellow oil (0.072 g, 49%). Using general procedure B, a sub-sample of this product (0.023 g, 0.027 mmol) was reacted with TFA (0.2 mL) in CH_2_Cl_2_ (2 mL) to afford the di-TFA salt **21d** as an orange oil (0.018 g, 76%). R*_f_* (MeOH/10% HCl, 7:3) 0.73; IR (ATR) ν_max_ 3405, 2944, 2857, 1674, 1632, 1505, 1439 cm^−1^; ^1^H NMR (DMSO-*d*_6_, 400 MHz) δ 12.92 (2H, s, NH-1), 8.93 (2H, t, *J* = 6.1 Hz, NH-10), 8.78 (2H, d, *J* = 3.4 Hz, H-2), 8.49 (4H, br s, NH_2_-14), 8.04 (2H, d, *J* = 7.9 Hz, H-4), 7.24 (2H, dd, *J* = 8.0, 8.0, 5.0 Hz, H-5), 7.13 (2H, dd, *J* = 11.2, 8.0 Hz, H-6), 3.30 (4H, dt, *J* = 6.5, 6.5 Hz, H_2_-11), 2.98–2.84 (8H, m, H_2_-13, H_2_-15), 1.89–1.82 (4H, m, H_2_-12), 1.57–1.52 (4H, br m, H_2_-16), 1.23 (12H, br s, H_2_-17, H_2_-18, H_2_-19); ^13^C NMR (DMSO-*d*_6_, 100 MHz) δ 181.9 (C-8), 163.4 (C-9), 149.3 (d, ^1^*J*_CF_ = 245 Hz, C-7), 138.8 (C-2), 129.8 (d, ^3^*J*_CF_ = 4.5 Hz, C-3a), 124.1 (d, ^2^*J*_CF_ = 13.2 Hz, C-7a), 123.4 (d, ^3^*J*_CF_ = 5.9 Hz, C-5), 117.4 (d, ^3^*J*_CF_ = 3.1 Hz, C-4), 112.8 (C-3), 108.7 (d, ^2^*J*_CF_ = 15.8 Hz, C-6), 46.8 (C-15), 44.7 (C-13), 35.9 (C-11), 28.7, 28.5 (C-18, C-19), 25.9, 25.6, 25.5 (C-12, C-16, C-17); (+)-HRESIMS [M+H]^+^ *m/z* 665.3639 (calcd for C_36_H_47_F_2_N_6_O_4_, 665.3621).

#### 3.2.22. *N*^1^,*N*^12^-Bis(3-(2-(7-fluoro-1*H*-indol-3-yl)-2-oxoacetamido)propyl)dodecane-1,12-diaminium 2,2,2-trifluoroacetate (**21e**)

Using general procedure A, 2-(7-fluoro-1*H*-indol-3-yl)-2-oxoacetyl chloride (**14**) (0.076 g, 0.034 mmol) was reacted with di-*tert*-butyl dodecane-1,12-diylbis((3-aminopropyl)carbamate) (**9e**) (0.087 g, 0.17 mmol) and DIPEA (0.18 mL, 1.0 mmol) to afford di-*tert*-butyl dodecane-1,12-diylbis((3-(2-(7-fluoro-1*H*-indol-3-yl)-2-oxoacetamido)propyl)carbamate) as a dark orange oil (0.047 g, 31%). Using general procedure B, a sub-sample of this product (0.045 g, 0.050 mmol) was reacted with TFA (0.2 mL) in CH_2_Cl_2_ (2 mL) to afford the di-TFA salt **21e** as an orange oil (0.026 g, 56%). R*_f_* (MeOH/10% HCl, 7:3) 0.70; IR (ATR) ν_max_ 3342, 2929, 1676, 1632, 1506, 1459 cm^−1^; ^1^H NMR (DMSO-*d*_6_, 400 MHz) δ 12.94 (2H, d, *J* = 2.7 Hz, NH-1), 8.93 (2H, t, *J* = 6.9 Hz, NH-10), 8.78 (2H, d, *J* = 3.4 Hz, H-2), 8.57 (4H, br s, NH_2_-14), 8.04 (2H, d, *J* = 8.5 Hz, H-4), 7.24 (2H, ddd, *J* = 7.8, 7.8, 5.0 Hz, H-5), 7.13 (2H, dd, *J* = 11.2, 8.0 Hz, H-6), 3.30 (4H, dt, *J* = 6.4, 6.4 Hz, H_2_-11), 2.94–2.84 (8H, m, H_2_-13, H_2_-15), 1.90–1.83 (4H, m, H_2_-12), 1.56 (4H, br s, H_2_-16), 1.27–1.22 (16H, m, H_2_-17, H_2_-18, H_2_-19, H_2_-20); ^13^C NMR (DMSO-*d*_6_, 100 MHz) δ 181.9 (C-8), 163.4 (C-9), 149.1 (d, ^1^*J*_CF_ = 241 Hz, C-7), 138.9 (C-2), 129.9 (d, ^3^*J*_CF_ = 4.2 Hz, C-3a), 124.1 (d, ^2^*J*_CF_ = 14.0 Hz, C-7a), 123.4 (d, ^3^*J*_CF_ = 6.1 Hz, C-5), 117.4 (d, ^3^*J*_CF_ = 3.1 Hz, C-4), 112.8 (C-3), 108.6 (d, ^2^*J*_CF_ = 16.1 Hz, C-6), 46.8 (C-15), 44.7 (C-13), 35.9 (C-11), 28.9, 28.8, 28.5 (C-18, C-19, C-20), 25.9, 25.6, 25.5 (C-12, C-16, C-17); (+)-HRESIMS [M+Na]^+^ *m/z* 715.3747 (calcd for C_38_H_50_F_2_N_6_NaO_4_, 715.3754).

#### 3.2.23. *N*^1^,*N*^6^-Bis(3-(2-(7-methoxy-1*H*-indol-3-yl)-2-oxoacetamido)propyl)hexane-1,6-diaminium 2,2,2-trifluoroacetate (**22a**)

Following general procedure C, 2-(7-methoxy-1*H*-indol-3-yl)-2-oxoacetic acid (**15**) (0.050 g, 0.23 mmol) was reacted with di-*tert*-butyl hexane-1,6-diylbis((3-aminopropyl) carbamate) (**9a**) (0.047 g, 0.11 mmol), PyBOP (0.119 g, 0.23 mmol) and DIPEA (0.06 mL, 0.34 mmol). Purification by column chromatography afforded di-*tert*-butyl hexane-1,6-diylbis((3-(2-(7-methoxy-1*H*-indol-3-yl)-2-oxoacetamido) propyl) carbamate) as a yellow oil (0.020 g, 22%). Using general procedure B, a sub-sample of this product (0.016 g, 0.019 mmol) was reacted with TFA (0.2 mL) in CH_2_Cl_2_ (2 mL) to afford the di-TFA salt **22a** as a black gum (0.013 g, 79%). R*_f_* (MeOH/10% HCl, 3:1) 0.68; IR (ATR) ν_max_ 3317, 2944, 1622, 1449, 1115, 1022, 721 cm^−1^; ^1^H NMR, (DMSO-*d*_6_, 400 MHz) δ 12.45 (2H, d, *J* = 2.0 Hz, NH-1), 8.89 (2H, t, *J* = 5.5 Hz, NH-10), 8.62 (2H, d, *J* = 3.5 Hz, H-2), 8.38 (4H, br s, NH-14), 7.80 (2H, d, *J* = 7.9 Hz, H-4), 7.19 (2H, t, *J* = 7.6 Hz, H-5), 6.86 (2H, d, *J* = 8.2 Hz, H-6), 3.95 (6H, s, OMe), 3.29 (4H, dt, *J* = 6.5, 6.5 Hz, H_2_-11), 2.92–2.88 (8H, m, H_2_-13, H_2_-15), 1.87–1.82 (4H, m, H_2_-12), 1.55 (4H, br s, H_2_-16), 1.31 (4H, br s, H_2_-17); ^13^C NMR (DMSO-*d*_6_, 100 MHz) δ 181.7 (C-8), 163.7 (C-9), 146.4 (C-7), 137.4 (C-2), 127.8 (C-3a), 126.1 (C-7a), 123.5 (C-5), 113.7 (C-4), 112.6 (C-3), 104.4 (C-6), 55.4 (OMe), 46.6 (C-15), 44.7 (C-13), 35.8 (C-11), 25.7, 25.5, 25.4 (C-12, C-16, C-17); (+)-HRESIMS *m/z* [M+H]^+^ 633.3408 (calcd for C_34_H_45_N_6_O_6_, 633.3395).

#### 3.2.24. *N*^1^,*N*^7^-Bis(3-(2-(7-methoxy-1*H*-indol-3-yl)-2-oxoacetamido)propyl)heptane-1,7-diaminium 2,2,2-trifluoroacetate (**22b**)

Using general procedure C, 2-(7-methoxy-1*H*-indol-3-yl)-2-oxoacetic acid (**15**) (0.086 g, 0.39 mmol) was reacted with di-*tert*-butyl heptane-1,7-diylbis((3-aminopropyl) carbamate) (**9b**) (0.088 g, 0.19 mmol), PyBOP (0.213 g, 0.41 mmol) and DIPEA (0.09 mL, 0.52 mmol). Purification by column chromatography afforded di-*tert*-butyl heptane-1,7-diylbis((3-(2-(7-methoxy-1*H*-indol-3-yl)-2-oxoacetamido)propyl)carbamate) as a yellow oil (0.066 g, 41%). Using general procedure B, a sub-sample of this product (0.030 g, 0.035 mmol) was reacted with TFA (0.2 mL) in CH_2_Cl_2_ (2 mL) to afford the di-TFA salt **22b** as a black gum (0.028 g, 90%). R*_f_* (MeOH/10% HCl, 3:1) 0.58; IR (ATR) ν_max_ 3317, 2943, 1675, 1432, 1132, 1022, 721 cm^−1^; ^1^H NMR, (DMSO-*d*_6_, 400 MHz) δ 12.45 (2H, d, *J* = 3.4 Hz, NH-1), 8.89 (2H, t, *J* = 6.2 Hz, NH-10), 8.62 (2H, d, *J* = 3.3 Hz, H-2), 8.35 (4H, br s, NH-14), 7.80 (2H, d, *J* = 7.7 Hz, H-4), 7.19 (2H, t, *J* = 7.9 Hz, H-5), 6.86 (2H, d, *J* = 7.9 Hz, H-6), 3.95 (6H, s, H_3_-19), 3.29 (4H, dt, *J* = 6.5, 6.5 Hz, H_2_-11), 2.96–2.84 (8H, br m, H_2_-13, H_2_-15), 1.87–1.82 (4H, m, H_2_-12), 1.55 (4H, br s, H_2_-16), 1.28 (6H, br s, H_2_-17, H_2_-18); ^13^C NMR (DMSO-*d*_6_, 100 MHz) δ 181.8 (C-8), 163.7 (C-9), 146.4 (C-7), 137.4 (C-2), 127.8 (C-3a), 126.1 (C-7a), 123.6 (C-5), 113.7 (C-4), 112.6 (C-3), 104.4 (C-6), 55.4 (C-19), 46.7 (C-15), 44.7 (C-13), 35.8 (C-11), 28.0 (C-18), 25.8, 25.7, 25.4 (C-12, C-16, C-17); (+)-HRESIMS [M+H]^+^ *m/z* 647.3550 (calcd for C_35_H_47_N_6_O_6_, 647.3552).

#### 3.2.25. *N*^1^,*N*^10^-Bis(3-(2-(7-methoxy-1*H*-indol-3-yl)-2-oxoacetamido)propyl)decane-1,10-diaminium 2,2,2-trifluoroacetate (**22d**)

Using general procedure C, 2-(7-methoxy-1*H*-indol-3-yl)-2-oxoacetic acid (**15**) (0.070 g, 0.32 mmol) was reacted with di-*tert*-butyl decane-1,10-diylbis((3-aminopropyl) carbamate) (**9d**) (0.078 g, 0.16 mmol), PyBOP (0.170 g, 0.32 mmol) and DIPEA (0.08 mL, 0.47 mmol). Purification by column chromatography afforded di-*tert*-butyl decane-1,10-diylbis((3-(2-(7-methoxy-1*H*-indol-3-yl)-2-oxoacetamido)propyl)carbamate) as a yellow oil (0.091 g, 65%). Using general procedure B, a sub-sample of this product (0.022 g, 0.025 mmol) was reacted with TFA (0.2 mL) in CH_2_Cl_2_ (2 mL) to afford the di-TFA salt **22d** as a black gum (0.022 g, 98%). R*_f_* (MeOH/10% HCl, 3:1) 0.47; IR (ATR) ν_max_ 3308, 2944, 1679, 1449, 1114, 1021 cm^−1^; ^1^H NMR, (DMSO-*d*_6_, 400 MHz) δ 12.45 (2H, d, *J* = 3.2 Hz, NH-1), 8.89 (2H, t, *J* = 6.1 Hz, NH-10), 8.62 (2H, d, *J* = 3.4 Hz, H-2), 8.39 (4H, br s, NH-14), 7.80 (2H, d, *J* = 7.7 Hz, H-4), 7.19 (2H, t, *J* = 7.9 Hz, H-5), 6.86 (2H, d, *J* = 7.7 Hz, H-6), 3.95 (6H, s, OMe), 3.29 (4H, dt, *J* = 6.7, 6.7 Hz, H_2_-11), 2.96–2.85 (8H, m, H_2_-13, H_2_-15), 1.87–1.80 (4H, m, H_2_-12), 1.56–1.53 (4H, m, H_2_-16), 1.24 (12H, br s, H_2_-17, H_2_-18, H_2_-19); ^13^C NMR (DMSO-*d*_6_, 100 MHz) δ 181.7 (C-8), 163.7 (C-9), 146.4 (C-7), 137.4 (C-2), 127.8 (C-3a), 126.1 (C-7a), 123.6 (C-5), 113.8 (C-4), 112.7 (C-3), 104.4 (C-6), 55.4 (OMe), 46.8 (C-15), 44.7 (C-13), 35.8 (C-11), 28.7, 28.5 (C-18, C-19), 25.9, 25.7, 25.5 (C-12, C-16, C-17); (+)-HRESIMS [M+H]^+^ *m/z* 689.4010 (calcd for C_38_H_53_N_6_O_6_, 689.4021).

#### 3.2.26. *N*^1^,*N*^6^-Bis(3-(2-(7-methyl-1*H*-indol-3-yl)-2-oxoacetamido)propyl)hexane-1,6-diaminium 2,2,2-trifluoroacetate (**23a**)

Using general procedure C, 2-(7-methyl-1*H*-indol-3-yl)-2-oxoacetic acid (**16**) (0.080 g, 0.39 mmol) was reacted with di-*tert*-butyl hexane-1,6-diylbis((3-aminopropyl) carbamate) (**9a**) (0.083 g, 0.19 mmol), PyBOP (0.204 g, 0.39 mmol) and DIPEA (0.1 mL, 0.57 mmol). Purification by column chromatography afforded di-*tert*-butyl hexane-1,6-diylbis((3-(2-(7-methyl-1*H*-indol-3-yl)-2-oxoacetamido) propyl) carbamate) as a yellow oil (0.019 g, 13%). Using general procedure B, a sub-sample of this product (0.010 g, 0.013 mmol) was reacted with TFA (0.2 mL) in CH_2_Cl_2_ (2 mL) to afford the di-TFA salt **23a** as a brown gum (0.010 g, 97%). R*_f_* (MeOH/10% HCl, 3:1) 0.34; IR (ATR) ν_max_ 3316, 2944, 1668, 1449, 1115, 1022, 721 cm^−1^; ^1^H NMR (DMSO-*d*_6_, 400 MHz) δ 12.34 (2H, d, *J* = 2.9 Hz, NH-1), 8.89 (2H, t, *J* = 6.1 Hz, NH-10), 8.73 (2H, d, *J* = 3.6 Hz, H-2), 8.45 (4H, br s, NH-14), 8.06 (2H, d, *J* = 7.8 Hz, H-4), 7.16 (2H, t, *J* = 7.8 Hz, H-5), 7.07 (2H, d, *J* = 7.3 Hz, H-6), 3.30 (4H, dt, *J* = 6.5, 6.5 Hz, H_2_-11), 2.92–2.88 (8H, m, H_2_-13, H_2_-15), 2.51 (6H, s, Me), 1.88–1.80 (4H, m, H_2_-12), 1.56 (4H, m, H_2_-16), 1.31 (4H, br s, H_2_-17); ^13^C NMR (DMSO-*d*_6_, 100 MHz) δ 181.7 (C-8), 163.8 (C-9), 138.0 (C-2), 135.7 (C-7a), 126.0 (C-3a), 124.1 (C-6), 122.8 (C-5), 121.9 (C-7), 118.8 (C-4), 112.5 (C-3), 46.7 (C-15), 44.7 (C-13), 35.8 (C-11), 25.7, 25.5, 25.4 (C-12, C-16, C-17), 16.6 (Me); (+)-HRESIMS [M+H]^+^ *m/z* 601.3486 (calcd for C_34_H_45_N_6_O_4_, 601.3497).

#### 3.2.27. *N*^1^,*N*^7^-Bis(3-(2-(7-methyl-1*H*-indol-3-yl)-2-oxoacetamido)propyl)heptane-1,7-diaminium 2,2,2-trifluoroacetate (**23b**)

Using general procedure C, 2-(7-methyl-1*H*-indol-3-yl)-2-oxoacetic acid (**16**) (0.070 g, 0.34 mmol) was reacted with di-*tert*-butyl heptane-1,7-diylbis((3-aminopropyl) carbamate) (**9b**) (0.075 g, 0.17 mmol), PyBOP (0.178 g, 0.34 mmol) and DIPEA (0.09 mL, 0.52 mmol). Purification by column afforded di-*tert*-butyl heptane-1,7-diylbis((3-(2-(7-methyl-1*H*-indol-3-yl)-2-oxoacetamido) propyl) carbamate) as a yellow oil (0.066 g, 48%). Using general procedure B, a sub-sample of this product (0.030 g, 0.036 mmol) was reacted with TFA (0.2 mL) in CH_2_Cl_2_ (2 mL) to afford the di-TFA salt **23b** as a brown gum (0.022 g, 71%). R*_f_* (MeOH/10% HCl, 3:1) 0.34; IR (ATR) ν_max_ 3326, 2944, 1678, 1449, 1114, 1022 cm^−1^; ^1^H NMR, (DMSO-*d*_6_, 400 MHz) δ 12.35 (2H, d, *J* = 2.8 Hz, NH-1), 8.89 (2H, t, *J* = 6.1 Hz, NH-10), 8.73 (2H, d, *J* = 3.4 Hz, H-2), 8.46 (4H, br s, NH-14), 8.06 (2H, d, *J* = 7.9 Hz, H-4), 7.16 (2H, t, *J* = 7.5 Hz, H-5), 7.07 (2H, d, *J* = 7.1 Hz, H-6), 3.30 (4H, dt, *J* = 6.5, 6.5 Hz, H_2_-11), 2.97–2.85 (8H, br m, H_2_-13, H_2_-15), 2.51 (6H, s, Me), 1.88–1.81 (4H, m, H_2_-12), 1.56 (4H, br s, H_2_-16), 1.28 (6H, br s, H_2_-17, H_2_-18); ^13^C NMR (DMSO-*d*_6_, 100 MHz) δ 181.7 (C-8), 163.8 (C-9), 138.1 (C-2), 135.7 (C-7a), 126.0 (C-3a), 124.1 (C-6), 122.8 (C-5), 121.9 (C-7), 118.8 (C-4), 112.4 (C-3), 46.7 (C-15), 44.7 (C-13), 35.8 (C-11), 28.0 (C-18), 25.8, 25.7, 25.4 (C-12, C-16, C-17), 16.6 (Me); (+)-HRESIMS [M+H]^+^ *m/z* 615.3644 (calcd for C_35_H_47_N_6_O_4_, 615.3653).

#### 3.2.28. *N*^1^,*N*^8^-Bis(3-(2-(7-methyl-1*H*-indol-3-yl)-2-oxoacetamido)propyl)octane-1,8-diaminium 2,2,2-trifluoroacetate (**23c**)

Using general procedure C, 2-(7-methyl-1*H*-indol-3-yl)-2-oxoacetic acid (**16**) (0.070 g, 3.4 mmol) was reacted with di-*tert*-butyl octane-1,8-diylbis((3-aminopropyl)carbamate) (**9c**) (0.077 g, 0.17 mmol), PyBOP (0.178 g, 0.34 mmol) and DIPEA (0.09 mL, 0.52 mmol). Purification by column chromatography afforded di-*tert*-butyl octane-1,8-diylbis((3-(2-(7-methyl-1*H*-indol-3-yl)-2-oxoacetamido)propyl)carbamate) as a yellow oil (0.102 g, 73%). Using general procedure B, a sub-sample of this product (0.080 g, 0.097 mmol) was reacted with TFA (0.2 mL) in CH_2_Cl_2_ (2 mL) to afford the di-TFA salt **23c** as a yellow oil (0.036 g, 44%). R*_f_* (MeOH/10% HCl, 3:1) 0.34; IR (ATR) ν_max_ 3325, 2944, 1678, 1448, 1116, 1021 cm^−1^; ^1^H NMR (DMSO-*d*_6_, 400 MHz) δ 12.35 (2H, d, *J* = 2.5 Hz, NH-1), 8.89 (2H, t, *J* = 6.0 Hz, NH-10), 8.74 (2H, d, *J* = 3.3 Hz, H-2), 8.47 (4H, br s, NH-14), 8.06 (2H, d, *J* = 7.8 Hz, H-4), 7.16 (2H, t, *J* = 7.5 Hz, H-5), 7.07 (2H, d, *J* = 7.1 Hz, H-6), 3.30 (4H, dt, *J* = 6.5, 6.5 Hz, H_2_-11), 2.98–2.85 (8H, br m, H_2_-13, H_2_-15), 2.51 (6H, s, Me), 1.88–1.81 (4H, m, H_2_-12), 1.56 (4H, br s, H_2_-16), 1.26 (8H, br s, H_2_-17, H_2_-18); ^13^C NMR (DMSO-*d*_6_, 100 MHz) δ 181.7 (C-8), 163.8 (C-9), 138.1 (C-2), 135.7 (C-7a), 126.1 (C-3a), 124.1 (C-6), 122.8 (C-5), 121.9 (C-7), 118.8 (C-4), 112.5 (C-3), 46.8 (C-15), 44.7 (C-13), 35.8 (C-11), 28.3 (C-18), 25.8, 25.7, 25.5 (C-12, C-16, C-17), 16.6 (Me); (+)-HRESIMS [M+H]^+^ *m/z* 629.3812 (calcd for C_36_H_49_N_6_O_4_, 629.3810).

#### 3.2.29. *N*^1^,*N*^10^-Bis(3-(2-(7-methyl-1*H*-indol-3-yl)-2-oxoacetamido)propyl)decane-1,10-diaminium 2,2,2-trifluoroacetate (**23d**)

Using general procedure C, 2-(7-methyl-1*H*-indol-3-yl)-2-oxoacetic acid (**16**) (0.070 g, 3.4 mmol) was reacted with di-*tert*-butyl decane-1,10-diylbis((3-aminopropyl) carbamate) (**9d**) (0.081 g, 0.17 mmol), PyBOP (0.179 g, 0.34 mmol) and DIPEA (0.09 mL, 0.52 mmol). Purification by column chromatography afforded di-*tert*-butyl decane-1,10-diylbis((3-(2-(7-methyl-1*H*-indol-3-yl)-2-oxoacetamido)propyl)carbamate) as a white solid (0.080 g, 55%). Using general procedure B, a sub-sample of this product (0.030 g, 0.035 mmol) was reacted with TFA (0.2 mL) in CH_2_Cl_2_ (2 mL) to afford the di-TFA salt **23d** as a brown oil (0.029 g, 94%). R*_f_* (MeOH/10% HCl, 3:1) 0.26; IR (ATR) ν_max_ 3312, 2944, 1678, 1449, 1117, 1021 cm^−1^; ^1^H NMR (DMSO-*d*_6_, 400 MHz) δ 12.35 (2H, d, *J* = 3.0 Hz, NH-1), 8.90 (2H, t, *J* = 6.1 Hz, NH-10), 8.73 (2H, d, *J* = 3.5 Hz, H-2), 8.44 (4H, br s, NH-14), 8.06 (2H, d, *J* = 7.8 Hz, H-4), 7.16 (2H, t, *J* = 7.5 Hz, H-5), 7.07 (2H, d, *J* = 7.2 Hz, H-6), 3.29 (4H, dt, *J* = 6.5, 6.5 Hz, H_2_-11), 2.97–2.85 (8H, m, H_2_-13, H_2_-15), 2.51 (6H, s, Me), 1.87–1.81 (4H, m, H_2_-12), 1.56–1.50 (4H, br m, H_2_-16), 1.24 (12H, br s, H_2_-17, H_2_-18, H_2_-19); ^13^C NMR (DMSO-*d*_6_, 100 MHz) δ 181.7 (C-8), 163.8 (C-9), 138.1 (C-2), 135.7 (C-7a), 126.1 (C-3a), 124.2 (C-6), 122.9 (C-5), 122.0 (C-7), 118.9 (C-4), 112.5 (C-3), 46.8 (C-15), 44.7 (C-13), 35.8 (C-11), 28.8, 28.5 (C-18, C-19), 26.0, 25.7, 25.5 (C-12, C-16, C-17), 16.6 (Me), (+)-HRESIMS [M+H]^+^ *m/z* 657.4130 (calcd for C_38_H_53_N_6_O_4_, 657.4123).

#### 3.2.30. *N*^1^,*N*^12^-Bis(3-(2-(7-methyl-1*H*-indol-3-yl)-2-oxoacetamido)propyl)dodecane-1,12-diaminium 2,2,2-trifluoroacetate (**23e**)

Using general procedure C, 2-(7-methyl-1*H*-indol-3-yl)-2-oxoacetic acid (**16**) (0.070 g, 0.34 mmol) was reacted with di-*tert*-butyl octane-1,8-diylbis((3-aminopropyl)carbamate) (**9e**) (0.086 g, 0.17 mmol), PyBOP (0.179 g, 0.34 mmol) and DIPEA (0.09 mL, 0.52 mmol). Purification by column chromatography afforded di-*tert*-butyl dodecane-1,12-diylbis((3-(2-(7-methyl-1*H*-indol-3-yl)-2-oxoacetamido)propyl)carbamate) as a yellow oil (0.145 g, 97%). Using general procedure B, a sub-sample of this product (0.084 g, 0.095 mmol) was reacted with TFA (0.2 mL) in CH_2_Cl_2_ (2 mL) to afford the di-TFA salt **23e** as a brown oil (0.057 g, 66%). R*_f_* (MeOH/10% HCl, 3:1) 0.23; IR (ATR) ν_max_ 3325, 2944, 1676, 1448, 1114, 1020 cm^−1^; ^1^H NMR (DMSO-*d*_6_, 400 MHz) δ 12.39 (2H, d, *J* = 2.7 Hz, NH-1), 8.90 (2H, t, *J* = 6.0 Hz, NH-10), 8.74 (2H, d, *J* = 3.2 Hz, H-2), 8.51 (4H, br s, NH-14), 8.07 (2H, d, *J* = 7.8 Hz, H-4), 7.16 (2H, t, *J* = 7.4 Hz, H-5), 7.07 (2H, d, *J* = 7.6 Hz, H-6), 3.30 (4H, dt, *J* = 6.5, 6.5 Hz, H_2_-11), 2.98–2.85 (8H, m, H_2_-13, H_2_-15), 2.51 (6H, s, Me), 1.85 (4H, tt, *J* = 6.5, 6.5 Hz, H_2_-12), 1.55 (4H, br m, H_2_-16), 1.22 (16H, br s, H_2_-17, H_2_-18, H_2_-19, H_2_-20); ^13^C NMR (DMSO-*d*_6_, 100 MHz) δ 181.7 (C-8), 163.8 (C-9), 138.1 (C-2), 135.7 (C-7a), 126.1 (C-3a), 124.1 (C-6), 122.8 (C-5), 122.0 (C-7), 118.8 (C-4), 112.5 (C-3), 46.8 (C-15), 44.7 (C-13), 35.8 (C-11), 29.0, 28.9, 28.6 (C-18, C-19, C-20), 25.9, 25.7, 25.5 (C-12, C-16, C-17), 16.6 (Me); (+)-HRESIMS [M+H]^+^ *m/z* 685.4421 (calcd for C_40_H_57_N_6_O_4_, 685.4436).

### 3.3. Antimicrobial Assays

The susceptibility of bacterial strains *S. aureus* (ATCC 25923), *E. coli* (ATCC 25922) and *P. aeruginosa* (ATCC 27853) to antibiotics and compounds was determined according to previously reported protocols [17]. Additional antimicrobial evaluation against MRSA (ATCC 43300) and *C. albicans* (ATCC 90028) was undertaken at the Community for Open Antimicrobial Drug Discovery at The University of Queensland (Australia) according to their standard protocols as reported previously [17,26].

### 3.4. Determination of the MICs of Antibiotics in the Presence of Synergizing Compounds

Antibiotic enhancing activities were determined according to previously reported protocols [14,17].

### 3.5. Cytotoxicity Assays

Cytotoxicity assays were conducted according to previously reported protocols [17,26].

### 3.6. Hemolytic Assay

Hemolysis assays were conducted according to previously reported protocols [17,26].

## 4. Conclusions

Our original screening for antimicrobial and antibiotic enhancing compounds from a library of marine natural products and their synthetic analogues identified a 6-bromoindolglyoxylamido-spermine conjugate as an active lead compound. Due to associated cytotoxicity and hemolytic properties, further efforts to explore the structure–activity relationship have investigated variation of substitution on the indole ring, and changes in the chain length of the polyamine fragment. While many analogues that were active as Gram-positive antibacterials were also associated with variable levels of cytotoxicity and/or hemolytic properties, the current study has identified two 7-methyl substituted analogues (**23b** and **23c**) with excellent anti-MRSA activity that are non-cytotoxic and non-hemolytic. This result defines a very narrow range of structural features required for optimal antibacterial properties. From the same set of analogues, only one example (**19a**), a 5-methoxy-PA3-6-3 conjugate, was non-toxic while also exhibiting strong tetracycline antibiotic enhancing activity towards *P. aeruginosa*. Further studies will be required to refine the mechanism of antibiotic enhancement.

## Figures and Tables

**Scheme 2:**
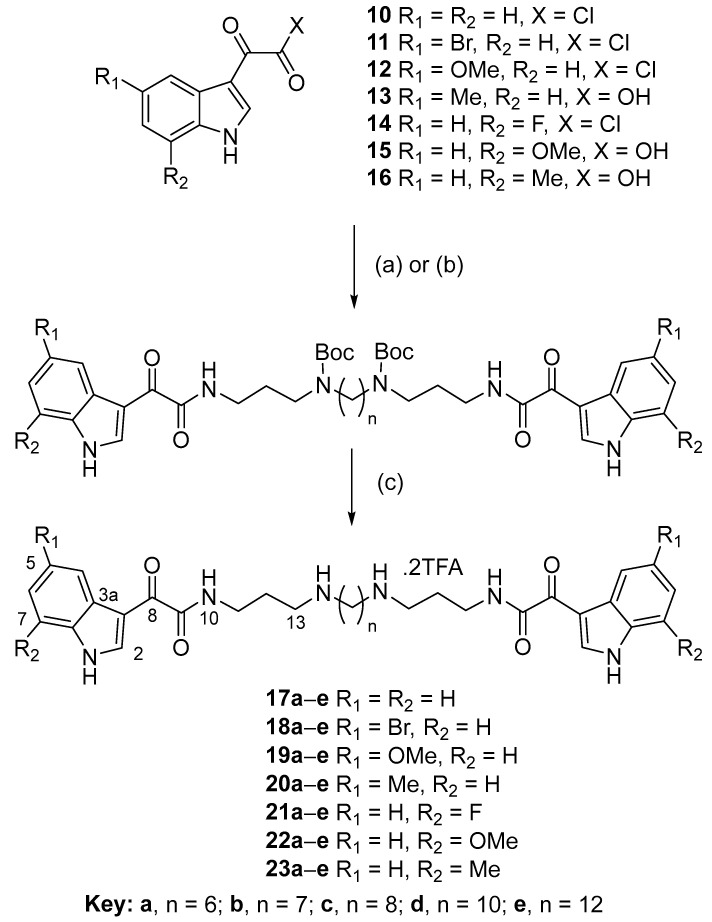
Synthetic route to target indolglyoxylpolyamine conjugates **17**–**23**. *Reagents and conditions*: (**a**) for glyoxylchlorides **10**–**12**, **14**: DMF, DIPEA, polyamine **9a**–**e**, r.t., 48 h (16–56%); (**b**) for glyoxylic acids **13**, **15**, **16**: DMF, PyBOP, DIPEA, polyamine **9a**–**e**, r.t., N_2_, 24 h (13–97%); and (**c**) TFA (0.2 mL) in CH_2_Cl_2_ (2 mL), N_2_, 2 h (19–100%).

**Table 4:** Antibiotic potentiating activity of **21a**.

Antibiotic	Concentration (µM) for Potentiation ^a^			
	*P. aeruginosa* ^b^	*E. coli* ^c^	*K. pneumoniae* ^d^	*A. baumannii* ^e^
No antibiotic	200	>200	>200	100
Doxycycline	3.125	1.56	200	12.5
Erythromycin	100	200	>200	100
Chloramphenicol	25	>200	>200	200
Nalidixic acid	25	200	>200	200

All values presented as the mean (n = 3). ^a^ Concentration (µM) of compound **21a** required to restore antibiotic activity at 2 µg/mL concentration of antibiotic; ^b^ *P. aeruginosa* ATCC 27853 against doxycycline (MIC 50 µM), erythromycin (MIC > 200 µM), chloramphenicol (MIC > 200 µM) and nalidixic acid (MIC > 200 μM); ^c^ *E. coli* ATCC 25922 against doxycycline (MIC 25 μM), erythromycin (MIC > 200 µM), chloramphenicol (MIC > 200 µM) and nalidixic acid (MIC > 200 μM); ^d^ *Klebsiella pneumoniae* ST258 against doxycycline (MIC 25 μM), erythromycin (MIC > 200 µM), chloramphenicol (MIC 50 µM) and nalidixic acid (MIC 100 μM); ^e^ *A. baumannii* AYE against doxycycline (MIC 12.5 μM), erythromycin (MIC 200 µM), chloramphenicol (MIC > 200 µM) and nalidixic acid (MIC > 200 μM).

## Data Availability

Data are contained within the article or Supplementary Material.

## Supplementary Materials

The following supporting information can be downloaded at: https://www.mdpi.com/article/10.3390/ph16060823/s1. Figure S1. ^1^H (DMSO-*d*_6_, 400 MHz) and ^13^C (DMSO-*d*_6_, 100 MHz) NMR spectra for **16**; Figure S2. ^1^H (DMSO-*d*_6_, 400 MHz) and ^13^C (DMSO-*d*_6_, 100 MHz) NMR spectra for **17a**; Figure S3. ^1^H (DMSO-*d*_6_, 400 MHz) and ^13^C (DMSO-*d*_6_, 100 MHz) NMR spectra for **17b**; Figure S4. ^1^H (DMSO-*d*_6_, 400 MHz) and ^13^C (DMSO-*d*_6_, 100 MHz) NMR spectra for **17d**; Figure S5. ^1^H (DMSO-*d*_6_, 400 MHz) and ^13^C (DMSO-*d*_6_, 100 MHz) NMR spectra for **18a**; Figure S6. ^1^H (DMSO-*d*_6_, 400 MHz) and ^13^C (DMSO-*d*_6_, 100 MHz) NMR spectra for **18b**; Figure S7. ^1^H (DMSO-*d*_6_, 400 MHz) and ^13^C (DMSO-*d*_6_, 100 MHz) NMR spectra for **18c**; Figure S8. ^1^H (DMSO-*d*_6_, 400 MHz) and ^13^C (DMSO-*d*_6_, 100 MHz) NMR spectra for **18d**; Figure S9. ^1^H (DMSO-*d*_6_, 400 MHz) and ^13^C (DMSO-*d*_6_, 100 MHz) NMR spectra for **18e**; Figure S10. ^1^H (DMSO-*d*_6_, 500 MHz) and ^13^C (DMSO-*d*_6_, 125 MHz) NMR spectra for **19a**; Figure S11. ^1^H (DMSO-*d*_6_, 400 MHz) and ^13^C (DMSO-*d*_6_, 100 MHz) NMR spectra for **19b**; Figure S12. ^1^H (DMSO-*d*_6_, 400 MHz) and ^13^C (DMSO-*d*_6_, 100 MHz) NMR spectra for **19d**; Figure S13. ^1^H (DMSO-*d*_6_, 400 MHz) and ^13^C (DMSO-*d*_6_, 100 MHz) NMR spectra for **20a**; Figure S14. ^1^H (DMSO-*d*_6_, 400 MHz) and ^13^C (DMSO-*d*_6_, 100 MHz) NMR spectra for **20b**; Figure S15. ^1^H (DMSO-*d*_6_, 400 MHz) and ^13^C (DMSO-*d*_6_, 100 MHz) NMR spectra for **20c**; Figure S16. ^1^H (DMSO-*d*_6_, 400 MHz) and ^13^C (DMSO-*d*_6_, 100 MHz) NMR spectra for **20d**; Figure S17. ^1^H (DMSO-*d*_6_, 400 MHz) and ^13^C (DMSO-*d*_6_, 100 MHz) NMR spectra for **20e**; Figure S18. ^1^H (DMSO-*d*_6_, 500 MHz) and ^13^C (DMSO-*d*_6_, 125 MHz) NMR spectra for **21a**; Figure S19. ^1^H (DMSO-*d*_6_, 400 MHz) and ^13^C (DMSO-*d*_6_, 100 MHz) NMR spectra for **21b**; Figure S20. ^1^H (DMSO-*d*_6_, 400 MHz) and ^13^C (DMSO-*d*_6_, 100 MHz) NMR spectra for **21c**; Figure S21. ^1^H (DMSO-*d*_6_, 400 MHz) and ^13^C (DMSO-*d*_6_, 100 MHz) NMR spectra for **21d**; Figure S22. ^1^H (DMSO-*d*_6_, 400 MHz) and ^13^C (DMSO-*d*_6_, 100 MHz) NMR spectra for **21e**; Figure S23. ^1^H (DMSO-*d*_6_, 400 MHz) and ^13^C (DMSO-*d*_6_, 100 MHz) NMR spectra for **22a**; Figure S24. ^1^H (DMSO-*d*_6_, 400 MHz) and ^13^C (DMSO-*d*_6_, 100 MHz) NMR spectra for **22b**; Figure S25. ^1^H (DMSO-*d*_6_, 400 MHz) and ^13^C (DMSO-*d*_6_, 100 MHz) NMR spectra for **22d**; Figure S26. ^1^H (DMSO-*d*_6_, 400 MHz) and ^13^C (DMSO-*d*_6_, 100 MHz) NMR spectra for **23a**; Figure S27. ^1^H (DMSO-*d*_6_, 400 MHz) and ^13^C (DMSO-*d*_6_, 100 MHz) NMR spectra for **23b**; Figure S28. ^1^H (DMSO-*d*_6_, 400 MHz) and ^13^C (DMSO-*d*_6_, 100 MHz) NMR spectra for **23c**; Figure S29. ^1^H (DMSO-*d*_6_, 400 MHz) and ^13^C (DMSO-*d*_6_, 100 MHz) NMR spectra for **23d**; Figure S30. ^1^H (DMSO-*d*_6_, 400 MHz) and ^13^C (DMSO-*d*_6_, 100 MHz) NMR spectra for **23e**.

## References

[1] Singh S.B. (2014). Confronting the Challenges of Discovery of Novel Antibacterial Agents. Bioorg. Med. Chem. Lett..

[2] Payne D.J., Gwynn M.N., Holmes D.J., Pompliano D.L. (2007). Drugs for Bad Bugs: Confronting the Challenges of Antibacterial Discovery. Nat. Rev. Drug Discov..

[3] Silver L.L. (2011). Challenges of Antibacterial Discovery. Clin. Microbiol. Rev..

[4] Bernal P., Molina-Santiago C., Daddaoua A., Llamas M.A. (2013). Antibiotic Adjuvants: Identification and Clinical Use: Antibiotic Adjuvants. Microb. Biotechnol..

[5] Farha M.A., Brown E.D. (2013). Discovery of Antibiotic Adjuvants. Nat. Biotechnol..

[6] González-Bello C. (2017). Antibiotic Adjuvants—A Strategy to Unlock Bacterial Resistance to Antibiotics. Bioorg. Med. Chem. Lett..

[7] Melander R.J., Melander C. (2017). The Challenge of Overcoming Antibiotic Resistance: An Adjuvant Approach?. ACS Infect. Dis..

[8] Douafer H., Andrieu V., Phanstiel O., Brunel J.M. (2019). Antibiotic Adjuvants: Make Antibiotics Great Again!. J. Med. Chem..

[9] Liu M., El-Hossary E.M., Oelschlaeger T.A., Donia M.S., Quinn R.J., Abdelmohsen U.R. (2019). Potential of Marine Natural Products against Drug-Resistant Bacterial Infections. Lancet Infect. Dis..

[10] Barbosa F., Pinto E., Kijjoa A., Pinto M., Sousa E. (2020). Targeting Antimicrobial Drug Resistance with Marine Natural Products. Int. J. Antimicrob. Agents.

[11] Valdes-Pena M.A., Massaro N.P., Lin Y.-C., Pierce J.G. (2021). Leveraging Marine Natural Products as a Platform to Tackle Bacterial Resistance and Persistence. Acc. Chem. Res..

[12] Salmi C., Loncle C., Vidal N., Letourneux Y., Fantini J., Maresca M., Taïeb N., Pagès J.-M., Brunel J.M. (2008). Squalamine: An Appropriate Strategy against the Emergence of Multidrug Resistant Gram-Negative Bacteria?. PLoS ONE.

[13] Lavigne J.-P., Brunel J.-M., Chevalier J., Pages J.-M. (2010). Squalamine, an Original Chemosensitizer to Combat Antibiotic-Resistant Gram-Negative Bacteria. J. Antimicrob. Chemother..

[14] Pieri C., Borselli D., Di Giorgio C., De Méo M., Bolla J.-M., Vidal N., Combes S., Brunel J.M. (2014). New Ianthelliformisamine Derivatives as Antibiotic Enhancers against Resistant Gram-Negative Bacteria. J. Med. Chem..

[15] Li S.A., Cadelis M.M., Sue K., Blanchet M., Vidal N., Brunel J.M., Bourguet-Kondracki M.-L., Copp B.R. (2019). 6-Bromoindolglyoxylamido Derivatives as Antimicrobial Agents and Antibiotic Enhancers. Bioorg. Med. Chem..

[16] Cadelis M.M., Pike E.I.W., Kang W., Wu Z., Bourguet-Kondracki M.-L., Blanchet M., Vidal N., Brunel J.M., Copp B.R. (2019). Exploration of the Antibiotic Potentiating Activity of Indolglyoxylpolyamines. Eur. J. Med. Chem..

[17] Chen D., Cadelis M.M., Rouvier F., Troia T., Edmeades L.R., Fraser K., Gill E.S., Bourguet-Kondracki M.-L., Brunel J.M., Copp B.R. (2023). α,ω-Diacyl-Substituted Analogues of Natural and Unnatural Polyamines: Identification of Potent Bactericides That Selectively Target Bacterial Membranes. Int. J. Mol. Sci..

[18] Klenke B., Gilbert I.H. (2001). Nitrile Reduction in the Presence of Boc-Protected Amino Groups by Catalytic Hydrogenation over Palladium-Activated Raney-Nickel. J. Org. Chem..

[19] Klenke B., Stewart M., Barrett M.P., Brun R., Gilbert I.H. (2001). Synthesis and Biological Evaluation of *s* -Triazine Substituted Polyamines as Potential New Anti-Trypanosomal Drugs. J. Med. Chem..

[20] Israel M., Rosenfield J.S., Modest E.J. (1964). Analogs of Spermine and Spermidine. I. Synthesis of Polymethylenepolyamines by Reduction of Cyanoethylated α,ι-Alkylenediamines1,2. J. Med. Chem..

[21] Pearce A.N., Kaiser M., Copp B.R. (2017). Synthesis and Antimalarial Evaluation of Artesunate-Polyamine and Trioxolane-Polyamine Conjugates. Eur. J. Med. Chem..

[22] Aubry C., Wilson A.J., Emmerson D., Murphy E., Chan Y.Y., Dickens M.P., García M.D., Jenkins P.R., Mahale S., Chaudhuri B. (2009). Fascaplysin-Inspired Diindolyls as Selective Inhibitors of CDK4/Cyclin D1. Bioorg. Med. Chem..

[23] Liew L.P.P., Fleming J.M., Longeon A., Mouray E., Florent I., Bourguet-Kondracki M.-L., Copp B.R. (2014). Synthesis of 1-Indolyl Substituted β-Carboline Natural Products and Discovery of Antimalarial and Cytotoxic Activities. Tetrahedron.

[24] Petit S., Duroc Y., Larue V., Giglione C., Léon C., Soulama C., Denis A., Dardel F., Meinnel T., Artaud I. (2009). Structure-Activity Relationship Analysis of the Peptide Deformylase Inhibitor 5-Bromo-1 *H*-Indole-3-Acetohydroxamic Acid. ChemMedChem.

[25] Wang J., Kaiser M., Copp B. (2014). Investigation of Indolglyoxamide and Indolacetamide Analogues of Polyamines as Antimalarial and Antitrypanosomal Agents. Mar. Drugs.

[26] Blaskovich M.A.T., Zuegg J., Elliott A.G., Cooper M.A. (2015). Helping Chemists Discover New Antibiotics. ACS Infect. Dis..

