# Supplementary material for: Structure–Activity Relationship Studies of Indolglyoxyl-Polyamine Conjugates as Antimicrobials and Antibiotic Potentiators
Source: Pharmaceuticals (Basel). 2023 May 31;16(6):823. doi: 10.3390/ph16060823 (PMC10302047; doi:10.3390/ph16060823)
Supplement: Supplementary file 1 [file pharmaceuticals-16-00823-s001.zip › pharmaceuticals-2397578-supplementary.pdf]

Supporting Information

Structure–Activity Relationship Studies of Indolglyoxyl-Polyamine Conjugates as Antimicrobials and Antibiotic Potentiators

Melissa M. Cadelis [1,2], Tim Liu [1], Kenneth Sue [1], Florent Rouvier [3], Marie-Lise Bourguet-Kondracki [4], Jean Michel Brunel [3] and Brent R. Copp [1,*]

1 School of Chemical Sciences, The University of Auckland, Private Bag 92019, Auckland 1142, New Zealand
2 School of Medical Sciences, The University of Auckland, Private Bag 92019, Auckland 1142, New Zealand
3 UMR MD1 "Membranes et Cibles Thérapeutiques", U1261 INSERM, Faculté de Pharmacie, Aix-Marseille Universite, 27 bd Jean Moulin, 13385 Marseille, France
4 Laboratoire Molécules de Communication et Adaptation des Micro-organismes, UMR 7245 CNRS, Muséum National d'Histoire Naturelle, 57 Rue Cuvier (C.P. 54), 75005 Paris, France
* Correspondence: b.copp@auckland.ac.nz

Contents

Figure S1 ^{1}H (DMSO-d_6, 400 MHz) and ^{13}C (DMSO-d_6, 100 MHz) NMR spectra for **16** S3
Figure S2 ^{1}H (DMSO-d_6, 400 MHz) and ^{13}C (DMSO-d_6, 100 MHz) NMR spectra for **17a** S4
Figure S3 ^{1}H (DMSO-d_6, 400 MHz) and ^{13}C (DMSO-d_6, 100 MHz) NMR spectra for **17b** S5
Figure S4 ^{1}H (DMSO-d_6, 400 MHz) and ^{13}C (DMSO-d_6, 100 MHz) NMR spectra for **17d** S6
Figure S5 ^{1}H (DMSO-d_6, 400 MHz) and ^{13}C (DMSO-d_6, 100 MHz) NMR spectra for **18a** S7
Figure S6 ^{1}H (DMSO-d_6, 400 MHz) and ^{13}C (DMSO-d_6, 100 MHz) NMR spectra for **18b** S8
Figure S7 ^{1}H (DMSO-d_6, 400 MHz) and ^{13}C (DMSO-d_6, 100 MHz) NMR spectra for **18c** S9
Figure S8 ^{1}H (DMSO-d_6, 400 MHz) and ^{13}C (DMSO-d_6, 100 MHz) NMR spectra for **18d** S10
Figure S9 ^{1}H (DMSO-d_6, 400 MHz) and ^{13}C (DMSO-d_6, 100 MHz) NMR spectra for **18e** S11
Figure S10 ^{1}H (DMSO-d_6, 500 MHz) and ^{13}C (DMSO-d_6, 125 MHz) NMR spectra for **19a** S12
Figure S11 ^{1}H (DMSO-d_6, 400 MHz) and ^{13}C (DMSO-d_6, 100 MHz) NMR spectra for **19b** S13
Figure S12 ^{1}H (DMSO-d_6, 400 MHz) and ^{13}C (DMSO-d_6, 100 MHz) NMR spectra for **19d** S14
Figure S13 ^{1}H (DMSO-d_6, 400 MHz) and ^{13}C (DMSO-d_6, 100 MHz) NMR spectra for **20a** S15
Figure S14 ^{1}H (DMSO-d_6, 400 MHz) and ^{13}C (DMSO-d_6, 100 MHz) NMR spectra for **20b** S16
Figure S15 ^{1}H (DMSO-d_6, 400 MHz) and ^{13}C (DMSO-d_6, 100 MHz) NMR spectra for **20c** S17
Figure S16 ^{1}H (DMSO-d_6, 400 MHz) and ^{13}C (DMSO-d_6, 100 MHz) NMR spectra for **20d** S18
Figure S17 ^{1}H (DMSO-d_6, 400 MHz) and ^{13}C (DMSO-d_6, 100 MHz) NMR spectra for **20e** S19
Figure S18 ^{1}H (DMSO-d_6, 500 MHz) and ^{13}C (DMSO-d_6, 125 MHz) NMR spectra for **21a** S20
Figure S19 ^{1}H (DMSO-d_6, 400 MHz) and ^{13}C (DMSO-d_6, 100 MHz) NMR spectra for **21b** S21
Figure S20 ^{1}H (DMSO-d_6, 400 MHz) and ^{13}C (DMSO-d_6, 100 MHz) NMR spectra for **21c** S22

Figure S21 ^{1}H (DMSO-d_6, 400 MHz) and ^{13}C (DMSO-d_6, 100 MHz) NMR spectra for **21d** S23
Figure S22 ^{1}H (DMSO-d_6, 400 MHz) and ^{13}C (DMSO-d_6, 100 MHz) NMR spectra for **21e** S24
Figure S23 ^{1}H (DMSO-d_6, 400 MHz) and ^{13}C (DMSO-d_6, 100 MHz) NMR spectra for **22a** S25
Figure S24 ^{1}H (DMSO-d_6, 400 MHz) and ^{13}C (DMSO-d_6, 100 MHz) NMR spectra for **22b** S26
Figure S25 ^{1}H (DMSO-d_6, 400 MHz) and ^{13}C (DMSO-d_6, 100 MHz) NMR spectra for **22d** S27
Figure S26 ^{1}H (DMSO-d_6, 400 MHz) and ^{13}C (DMSO-d_6, 100 MHz) NMR spectra for **23a** S28
Figure S27 ^{1}H (DMSO-d_6, 400 MHz) and ^{13}C (DMSO-d_6, 100 MHz) NMR spectra for **23b** S29
Figure S28 ^{1}H (DMSO-d_6, 400 MHz) and ^{13}C (DMSO-d_6, 100 MHz) NMR spectra for **23c** S30
Figure S29 ^{1}H (DMSO-d_6, 400 MHz) and ^{13}C (DMSO-d_6, 100 MHz) NMR spectra for **23d** S31
Figure S30 ^{1}H (DMSO-d_6, 400 MHz) and ^{13}C (DMSO-d_6, 100 MHz) NMR spectra for **23e** S32

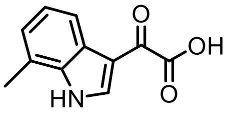

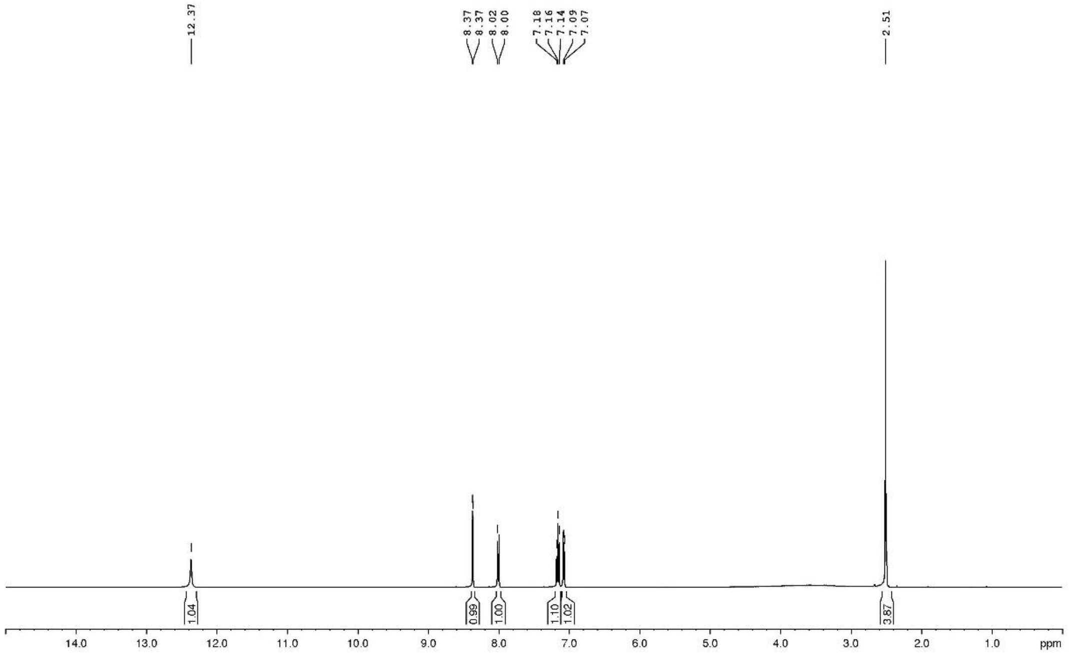

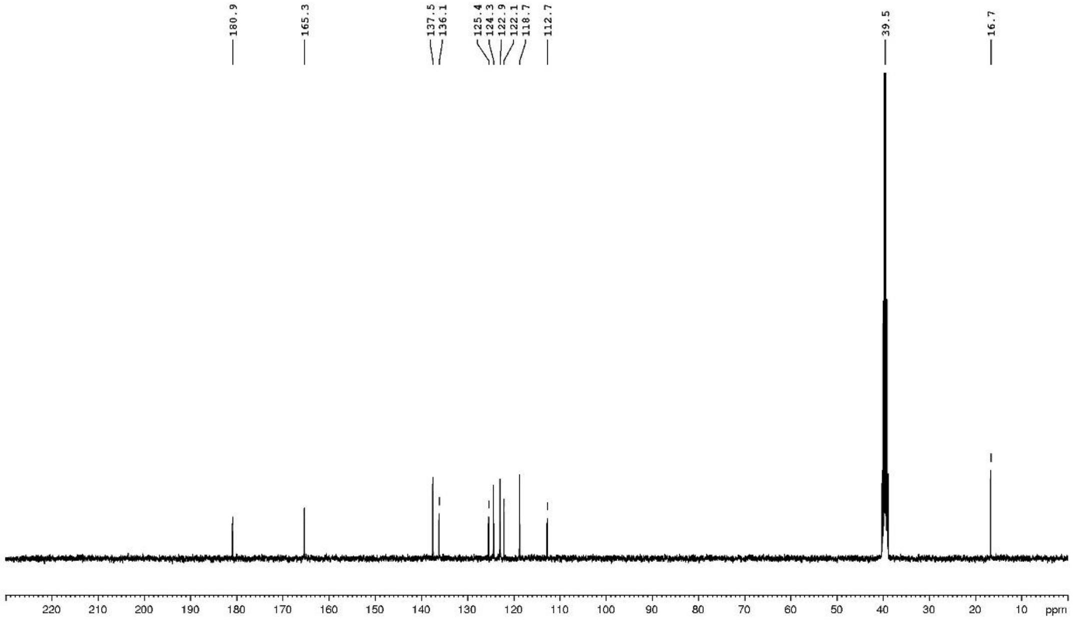

Figure S1 ^{1}H (DMSO-d_6, 400 MHz) and ^{13}C (DMSO-d_6, 100 MHz) NMR spectra for **16**

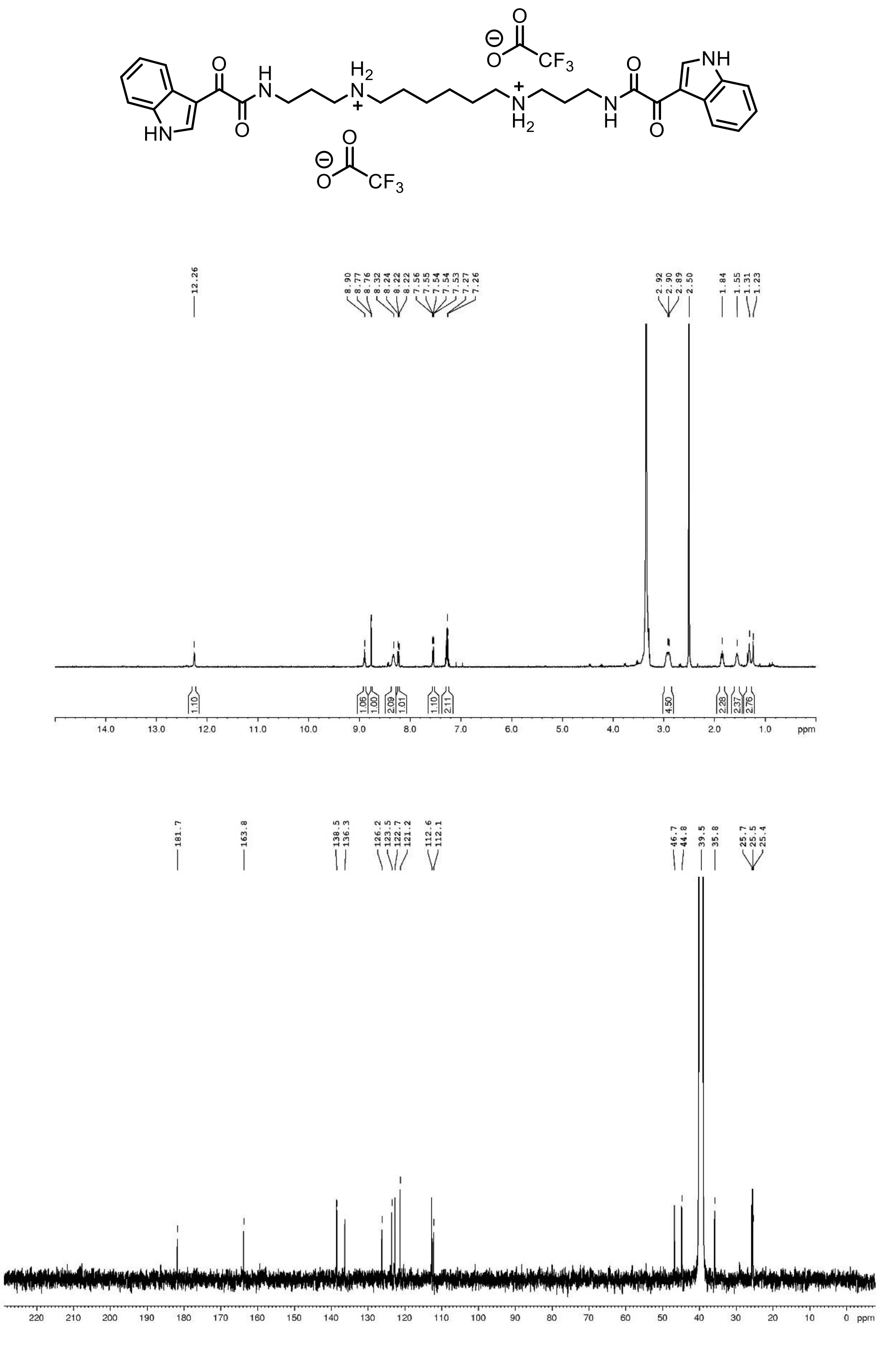

Figure S2 ^{1}H (DMSO-d_6, 400 MHz) and ^{13}C (DMSO-d_6, 100 MHz) NMR spectra for **17a**

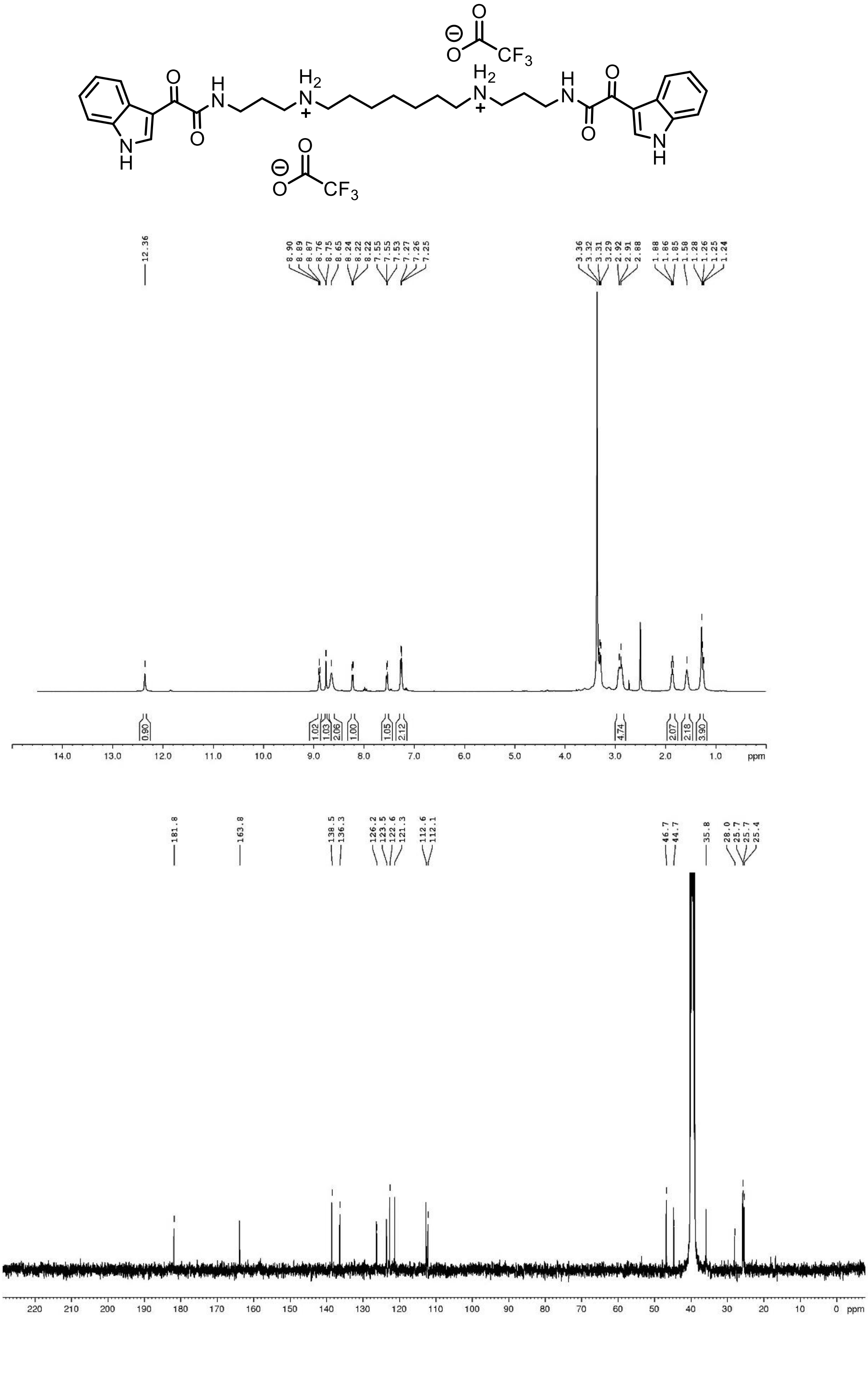

Figure S3 ^{1}H (DMSO-d_6, 400 MHz) and ^{13}C (DMSO-d_6, 100 MHz) NMR spectra for **17b**

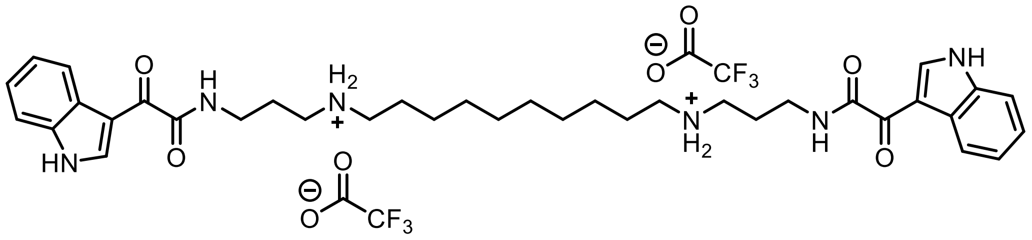

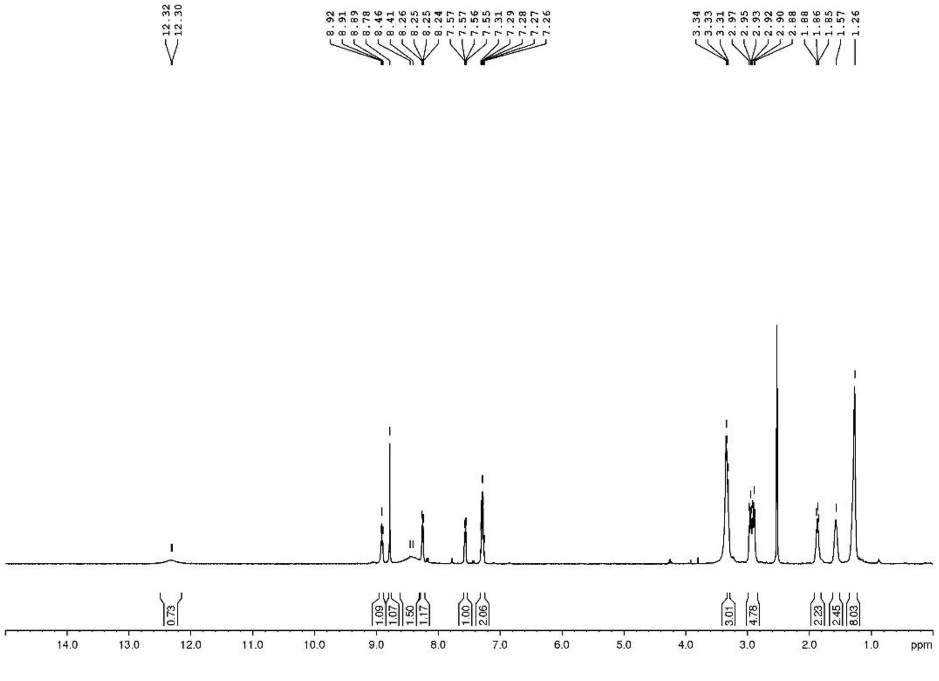

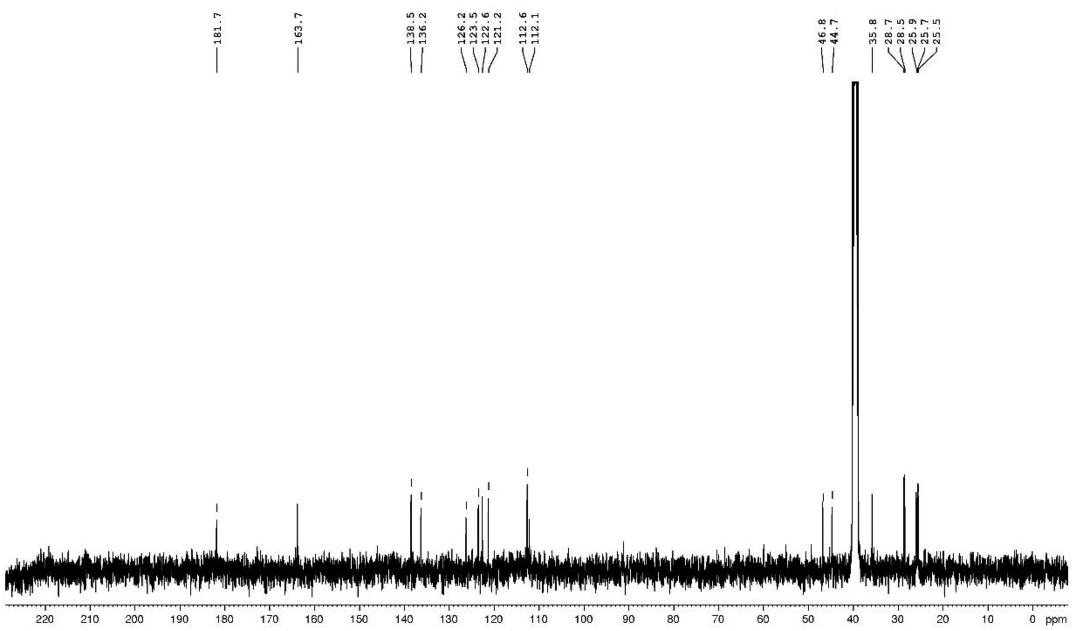

Figure S4 ^{1}H (DMSO-d_6, 400 MHz) and ^{13}C (DMSO-d_6, 100 MHz) NMR spectra for **17d**

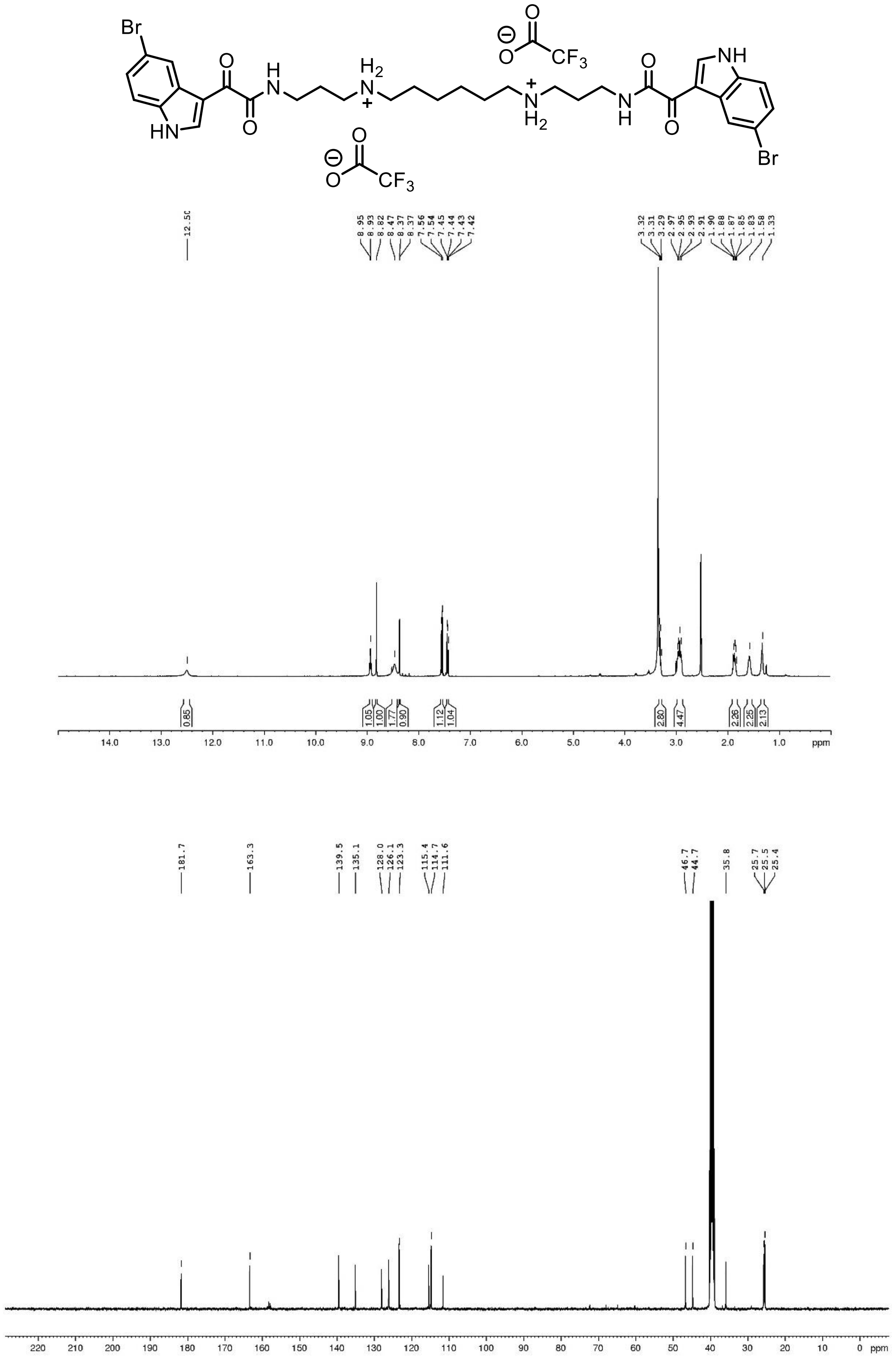

Figure S5 ^{1}H (DMSO-d_6, 400 MHz) and ^{13}C (DMSO-d_6, 100 MHz) NMR spectra for **18a**

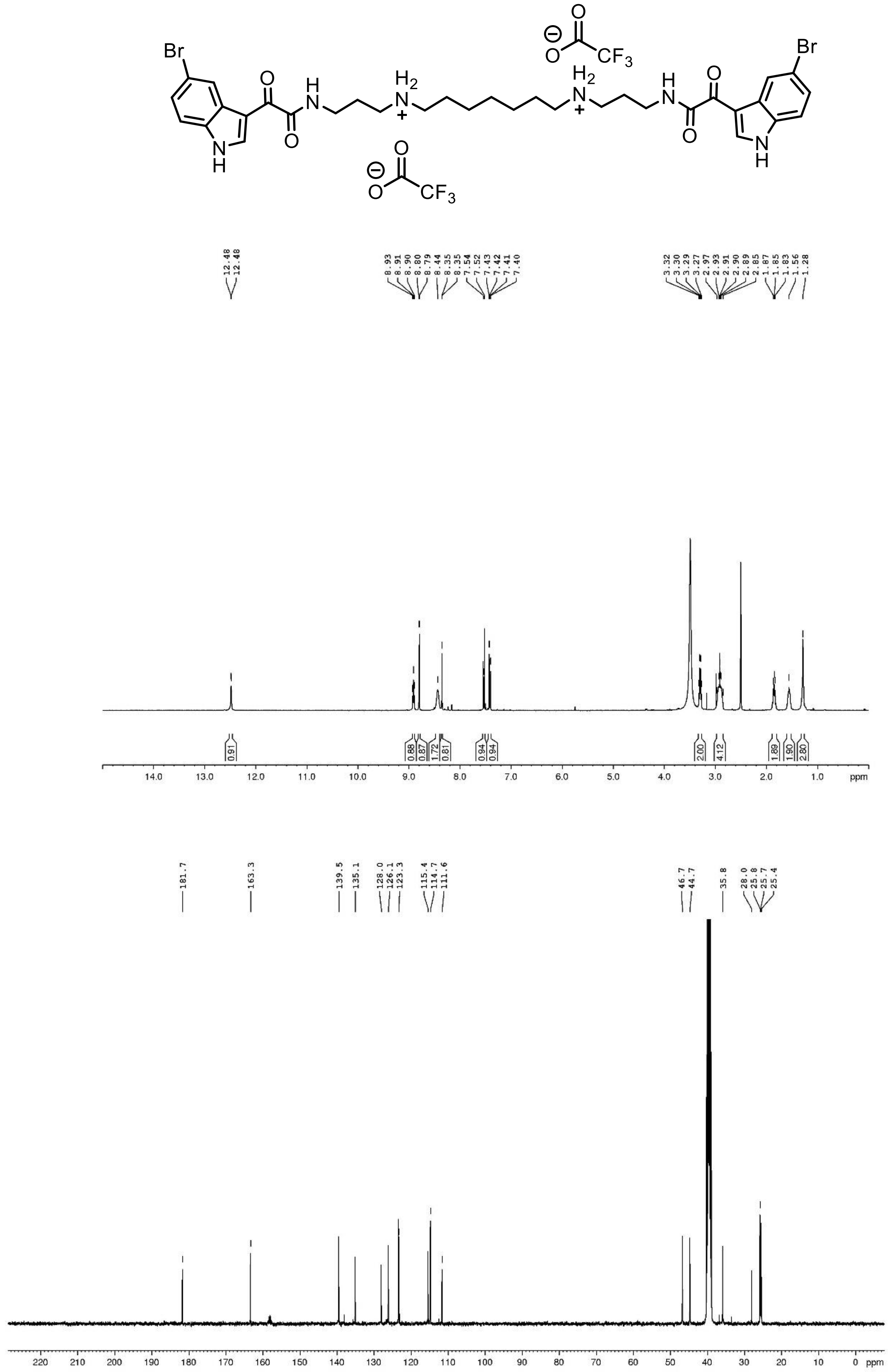

Figure S6 ^{1}H (DMSO-d_6, 400 MHz) and ^{13}C (DMSO-d_6, 100 MHz) NMR spectra for **18b**

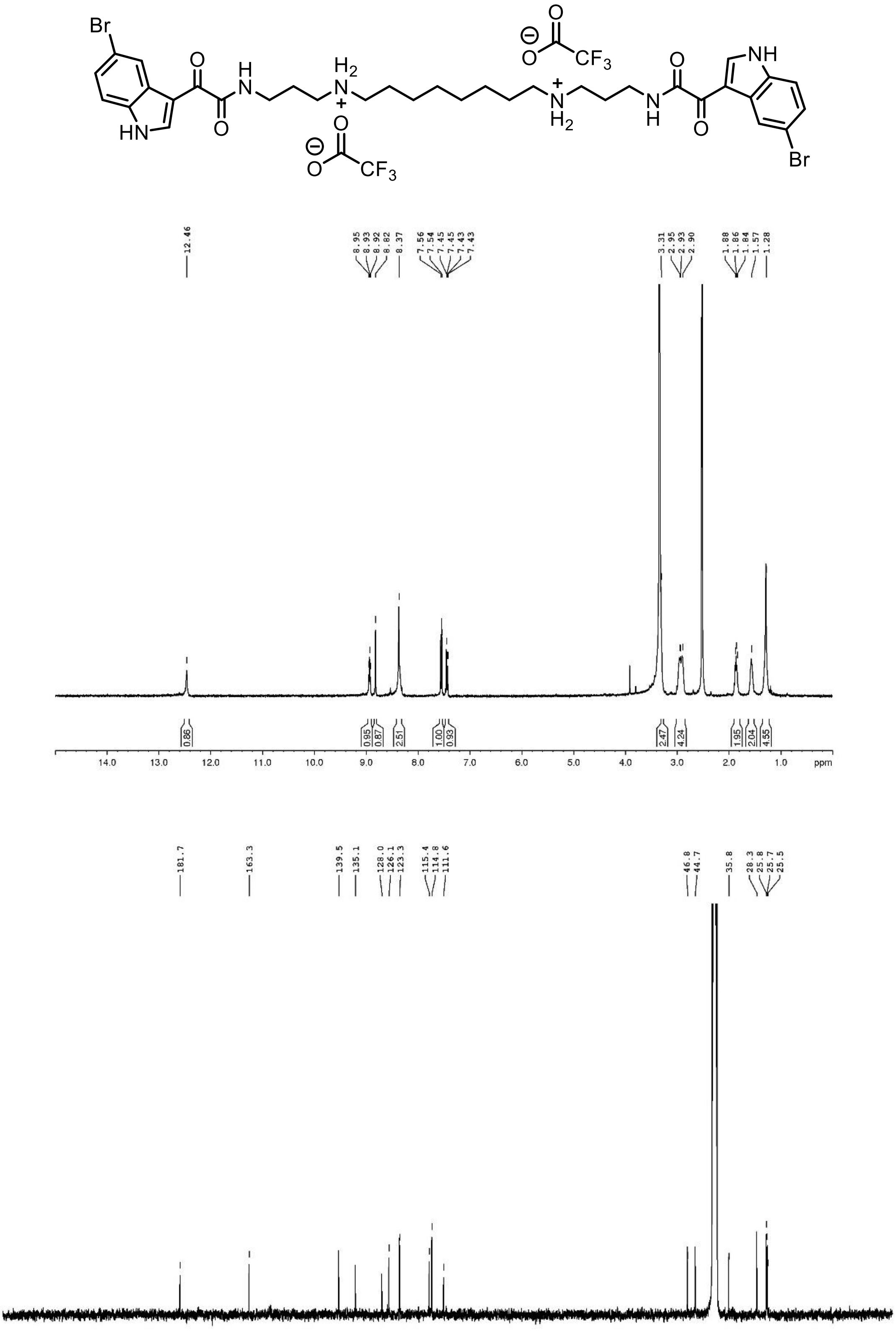

Figure S7 ^{1}H (DMSO-d_6, 400 MHz) and ^{13}C (DMSO-d_6, 100 MHz) NMR spectra for **18c**

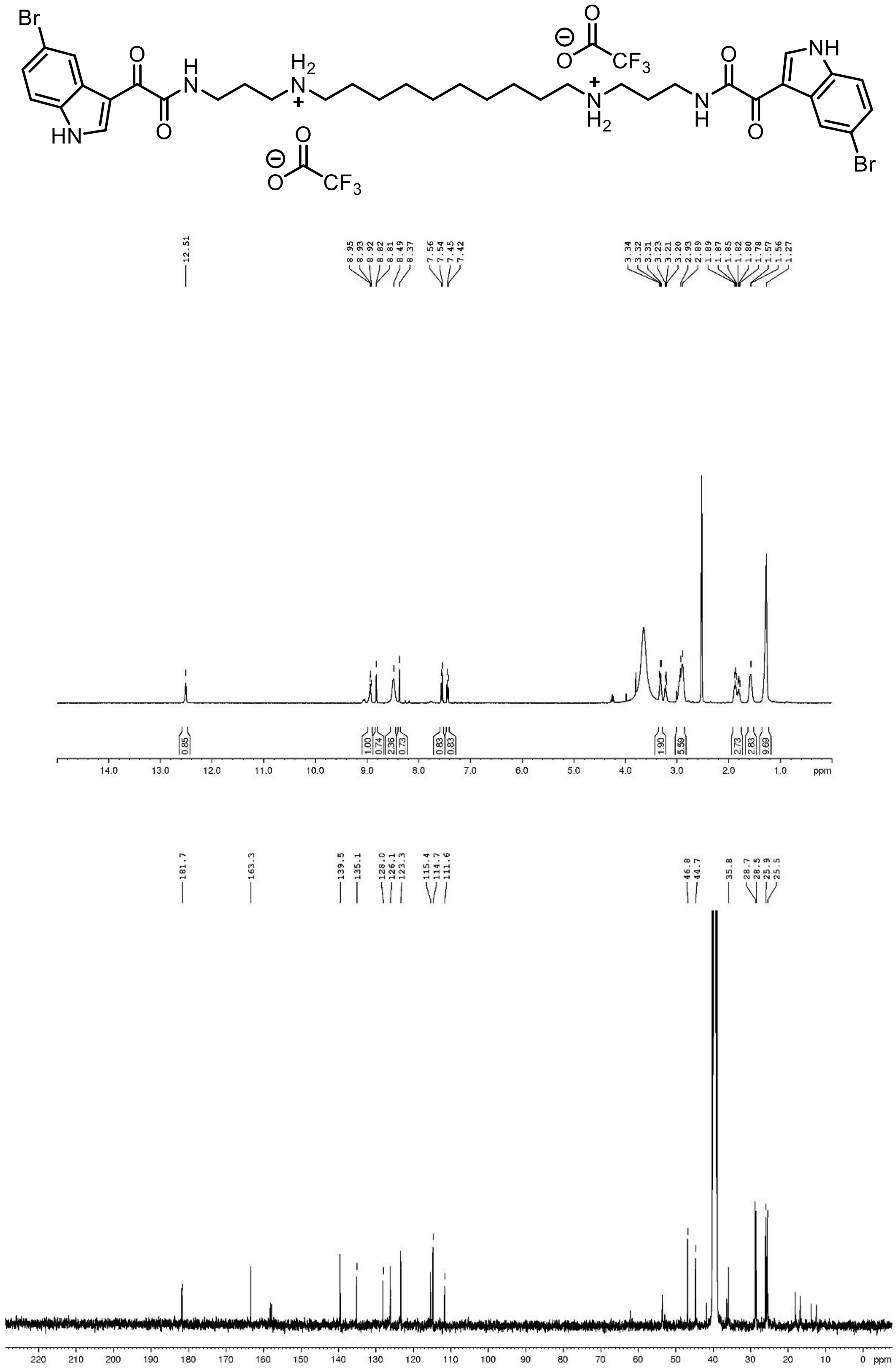

Figure S8 ^{1}H (DMSO-d_6, 400 MHz) and ^{13}C (DMSO-d_6, 100 MHz) NMR spectra for **18d**

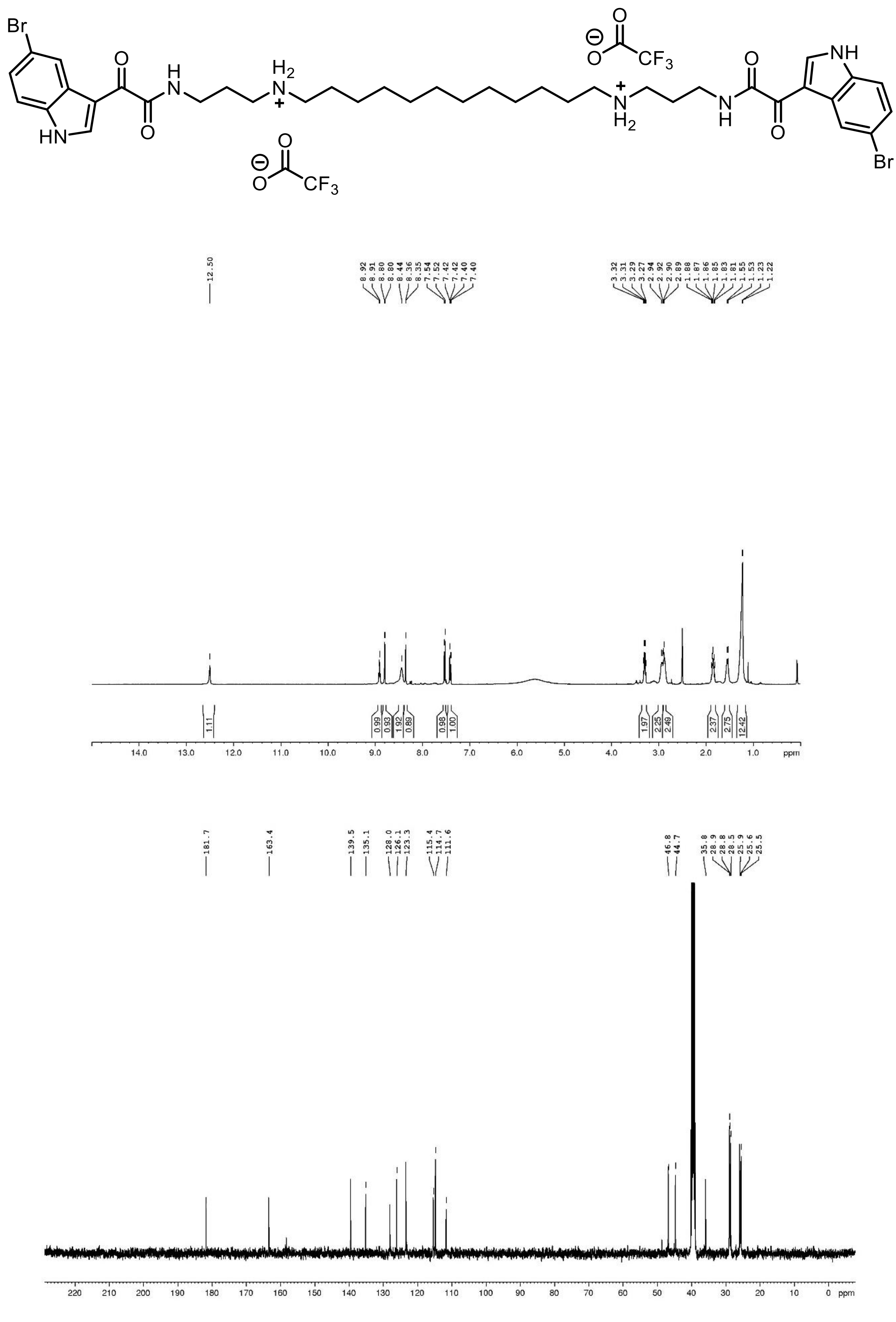

Figure S9 ^{1}H (DMSO-d_6, 400 MHz) and ^{13}C (DMSO-d_6, 100 MHz) NMR spectra for **18e**

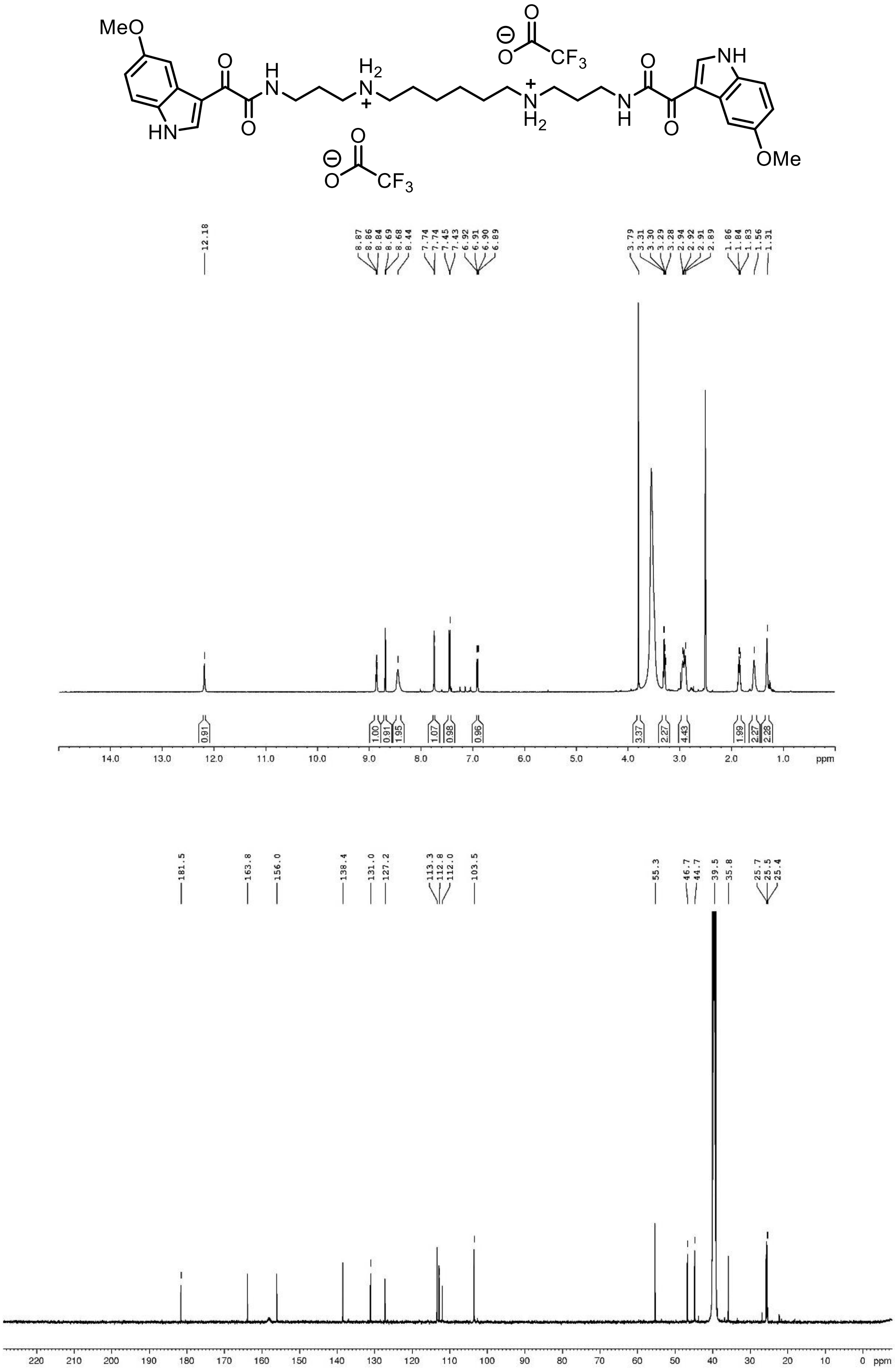

Figure S10 ^{1}H (DMSO-d_6, 500 MHz) and ^{13}C (DMSO-d_6, 125 MHz) NMR spectra for **19a**

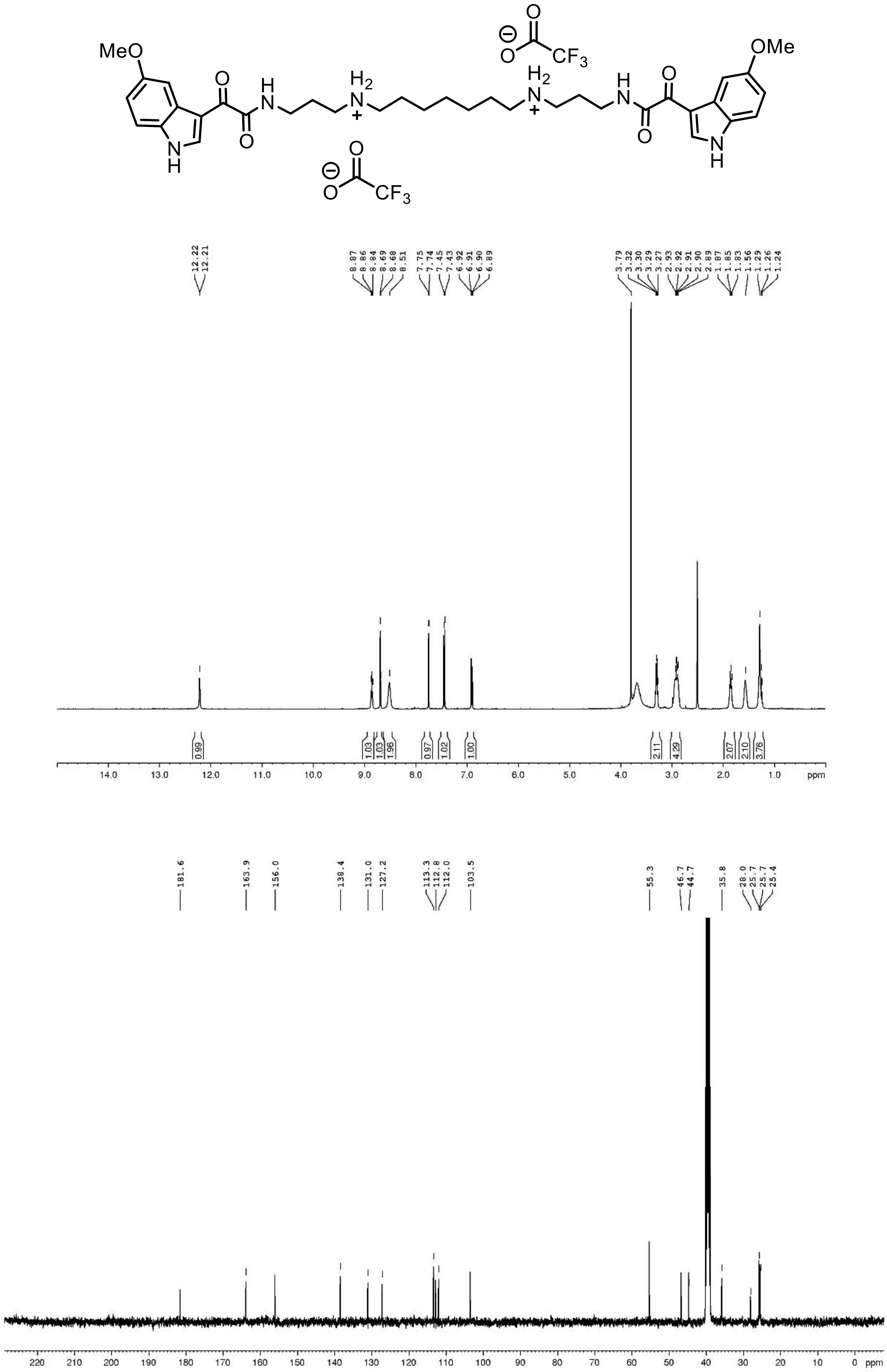

Figure S11 ^{1}H (DMSO-d_6, 400 MHz) and ^{13}C (DMSO-d_6, 100 MHz) NMR spectra for **19b**

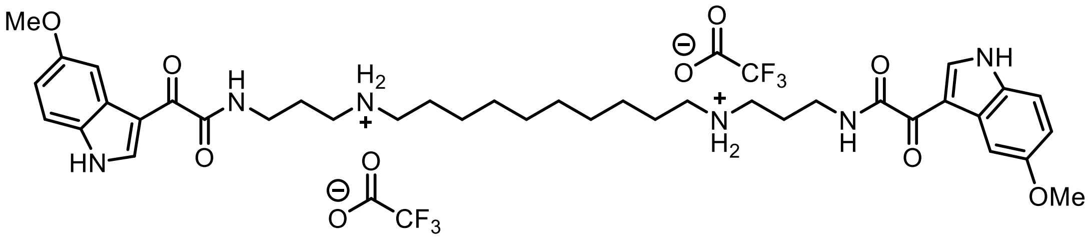

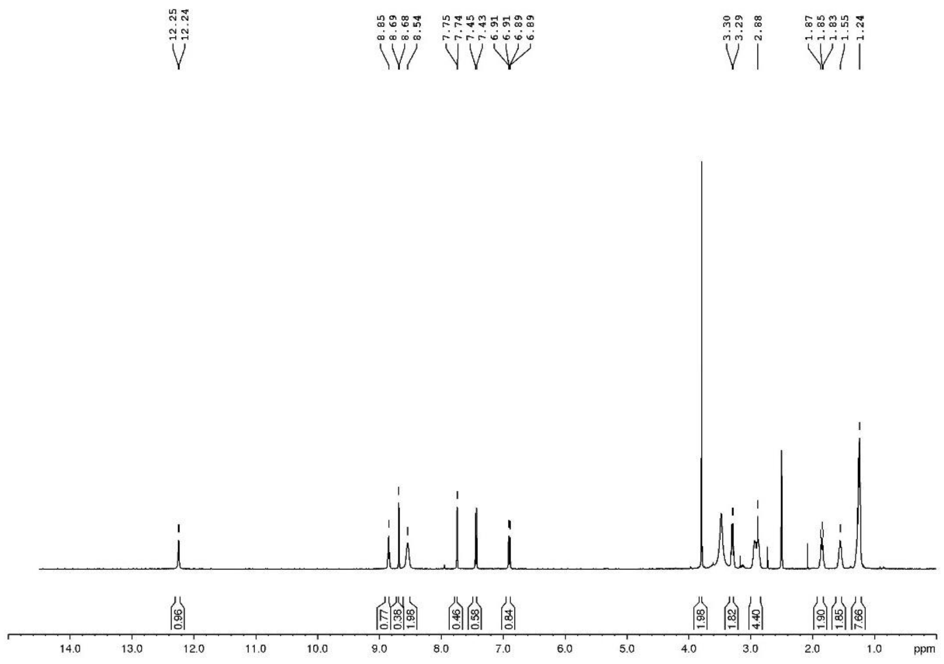

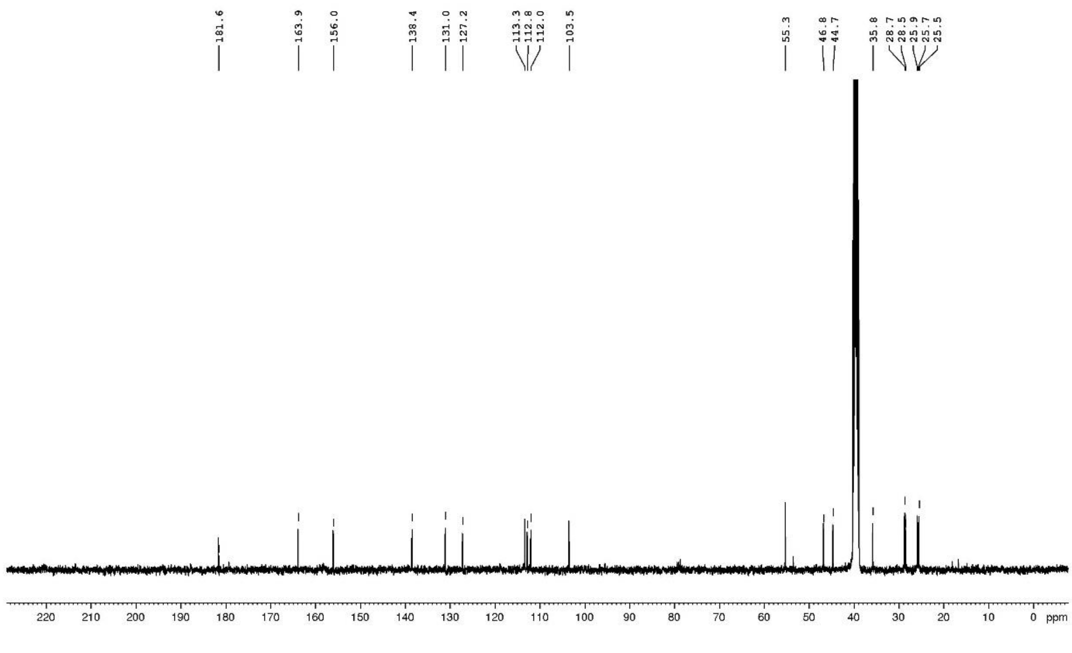

Figure S12 ^{1}H (DMSO-d_6, 400 MHz) and ^{13}C (DMSO-d_6, 100 MHz) NMR spectra for **19d**

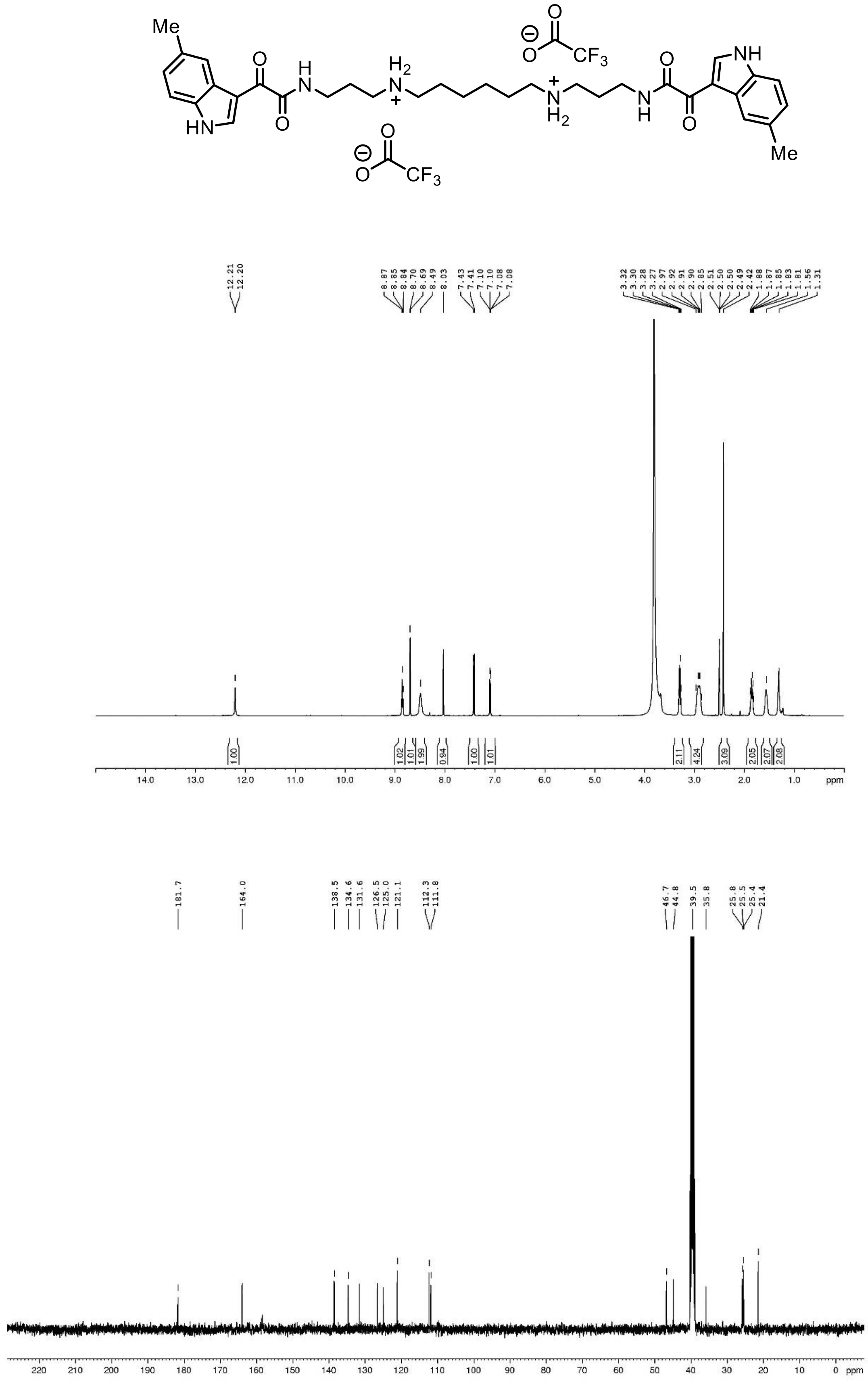

Figure S13 ^{1}H (DMSO-d_6, 400 MHz) and ^{13}C (DMSO-d_6, 100 MHz) NMR spectra for **20a**

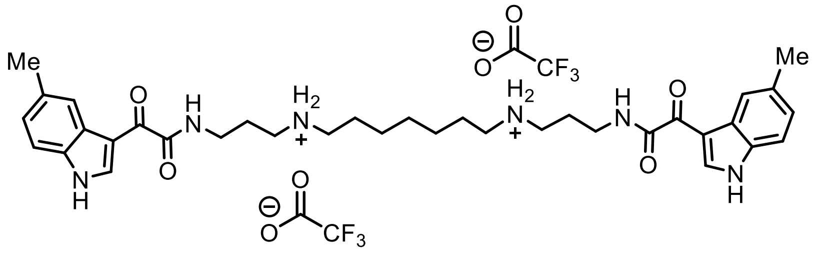

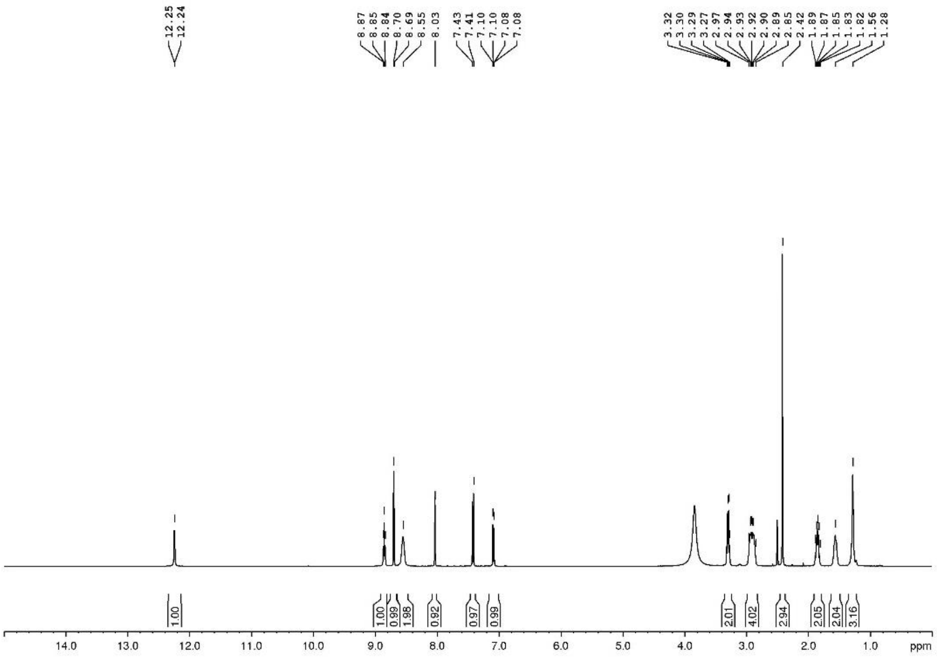

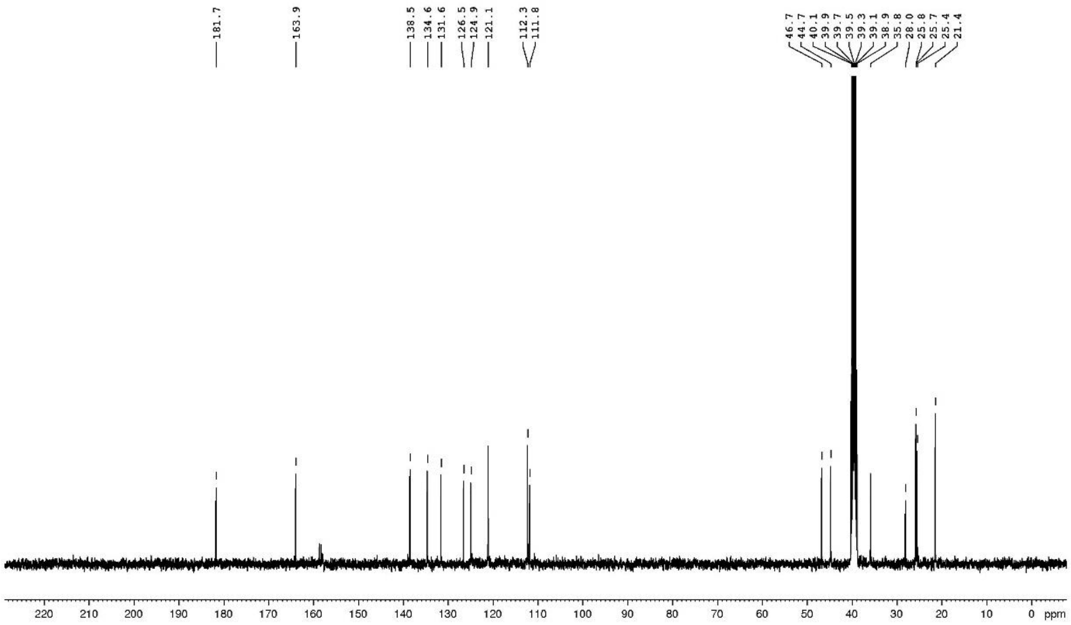

Figure S14 ^{1}H (DMSO-d_6, 400 MHz) and ^{13}C (DMSO-d_6, 100 MHz) NMR spectra for **20b**

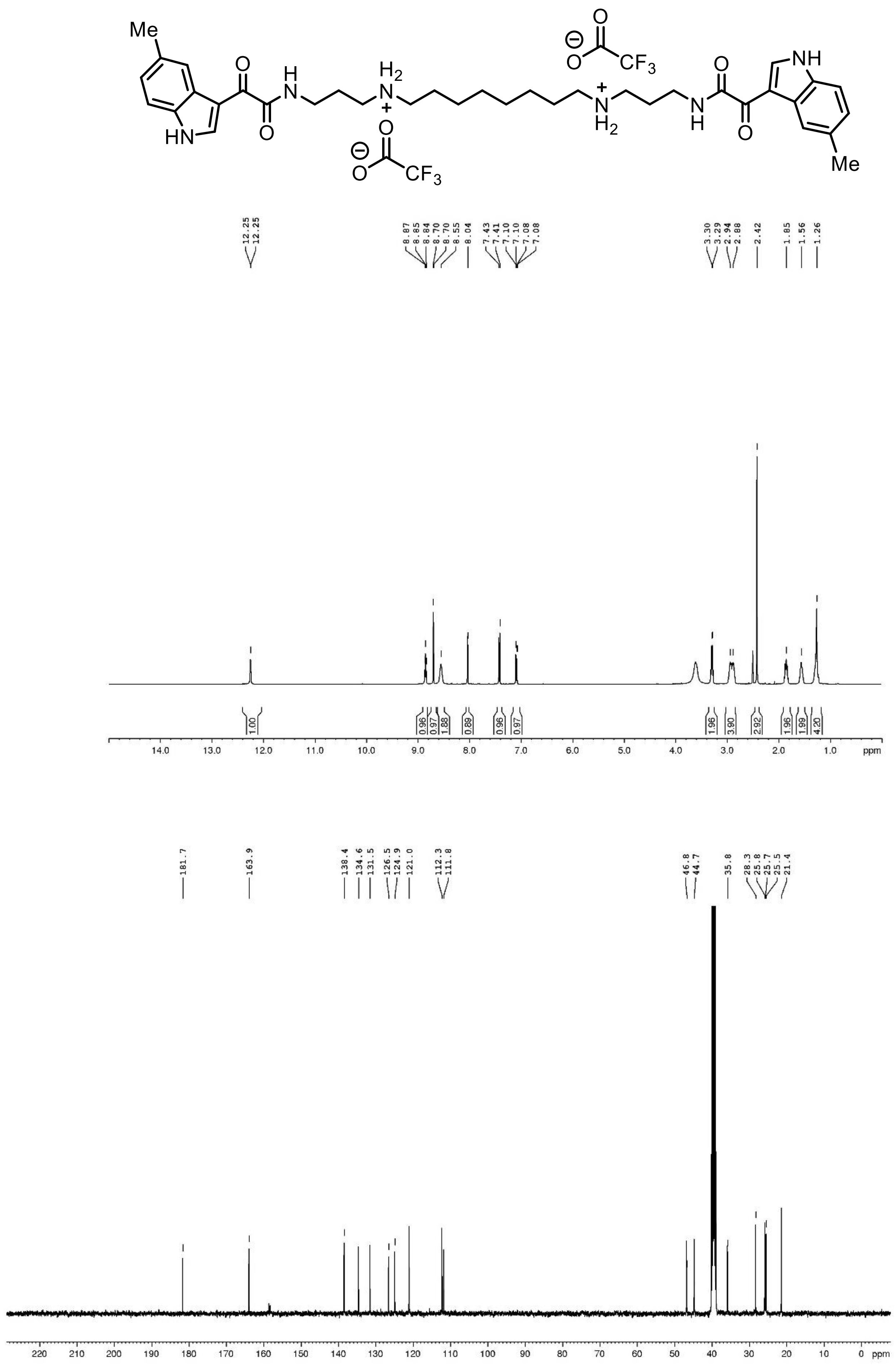

Figure S15 ^{1}H (DMSO-d_6, 400 MHz) and ^{13}C (DMSO-d_6, 100 MHz) NMR spectra for **20c**

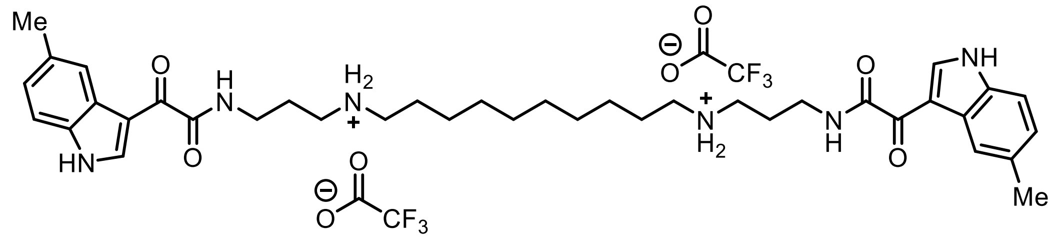

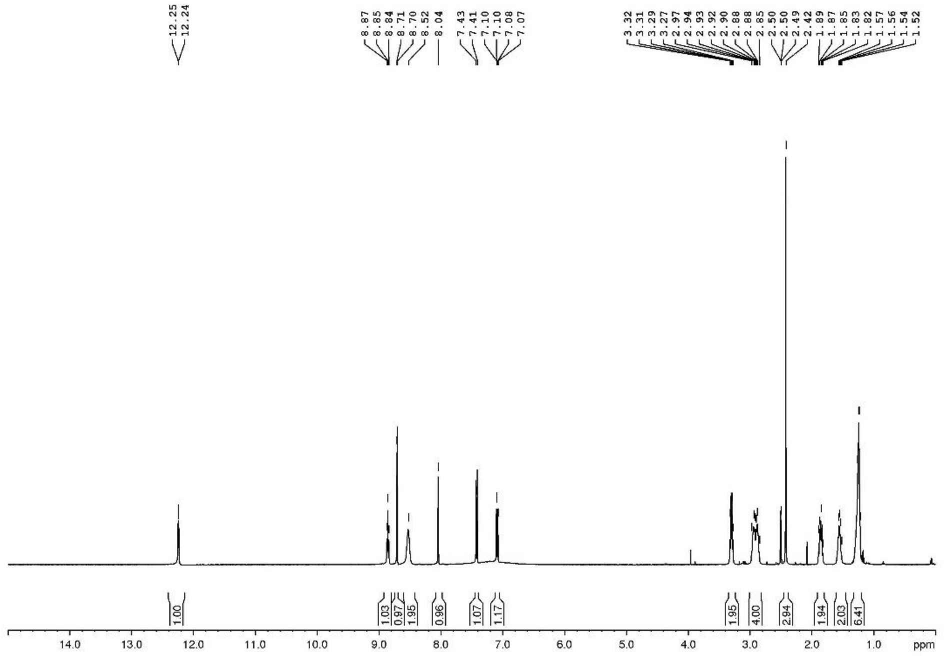

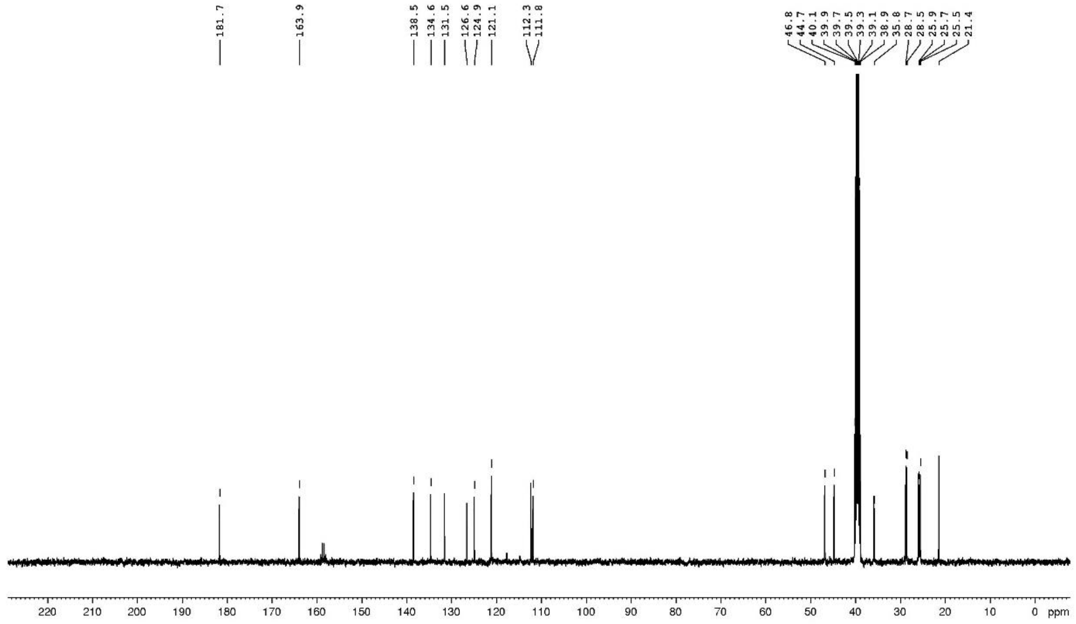

Figure S16 ^{1}H (DMSO-d_6, 400 MHz) and ^{13}C (DMSO-d_6, 100 MHz) NMR spectra for **20d**

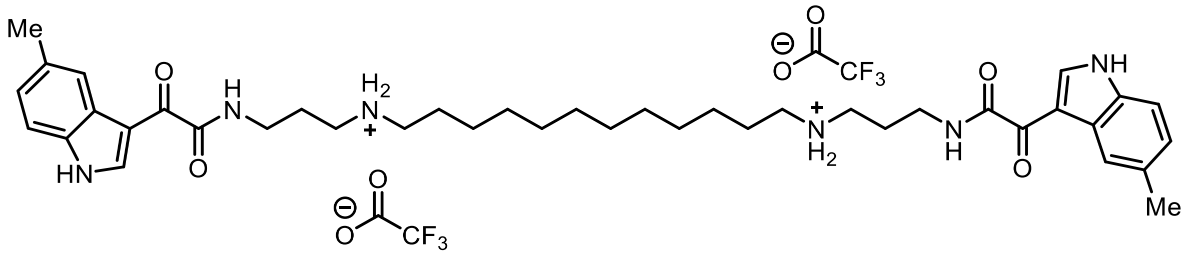

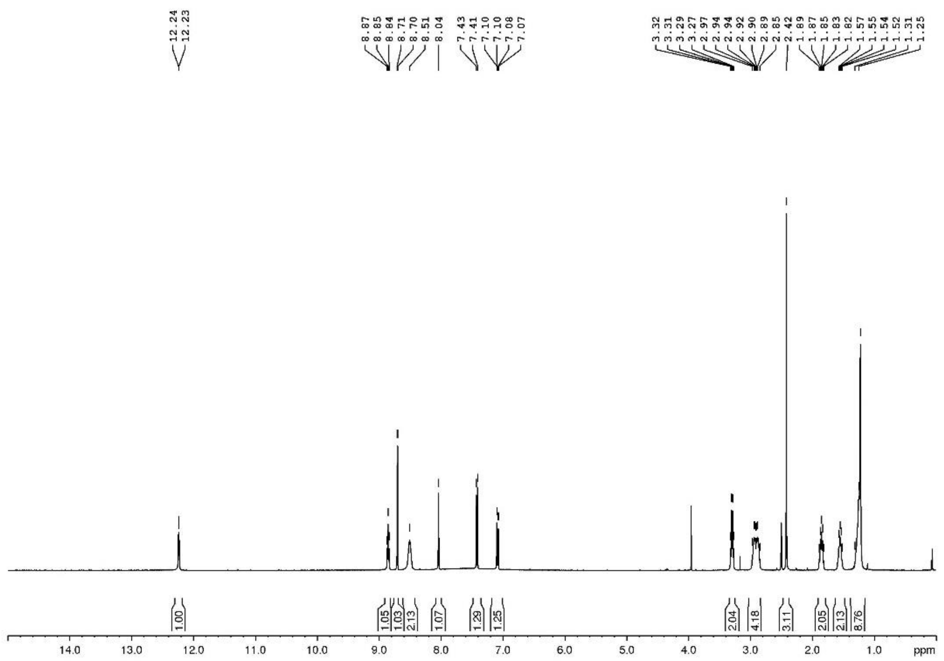

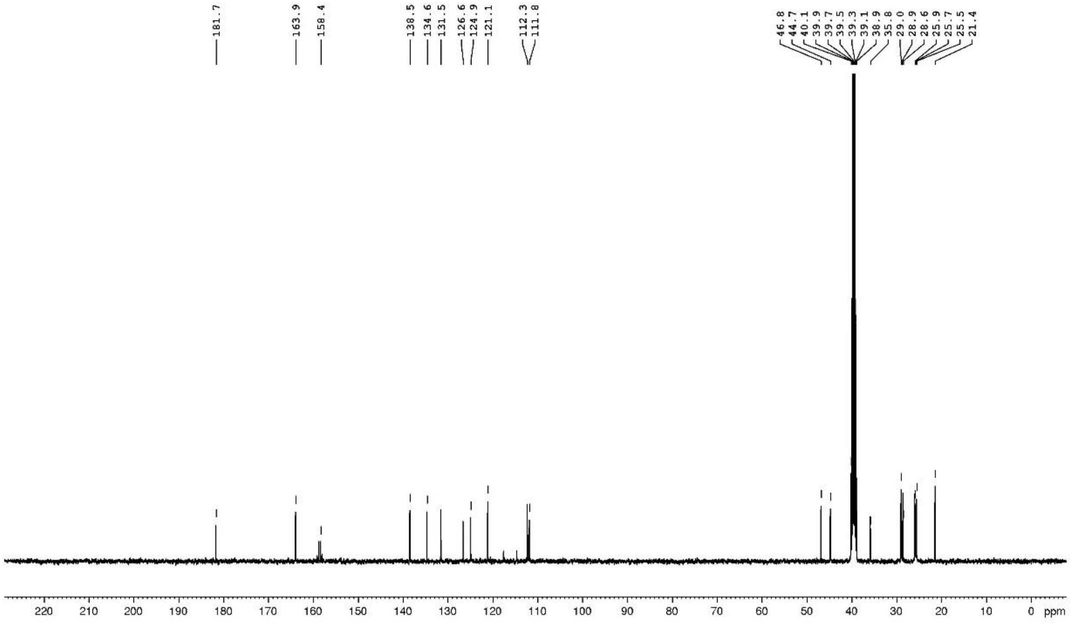

Figure S17 ^{1}H (DMSO-d_6, 400 MHz) and ^{13}C (DMSO-d_6, 100 MHz) NMR spectra for **20e**

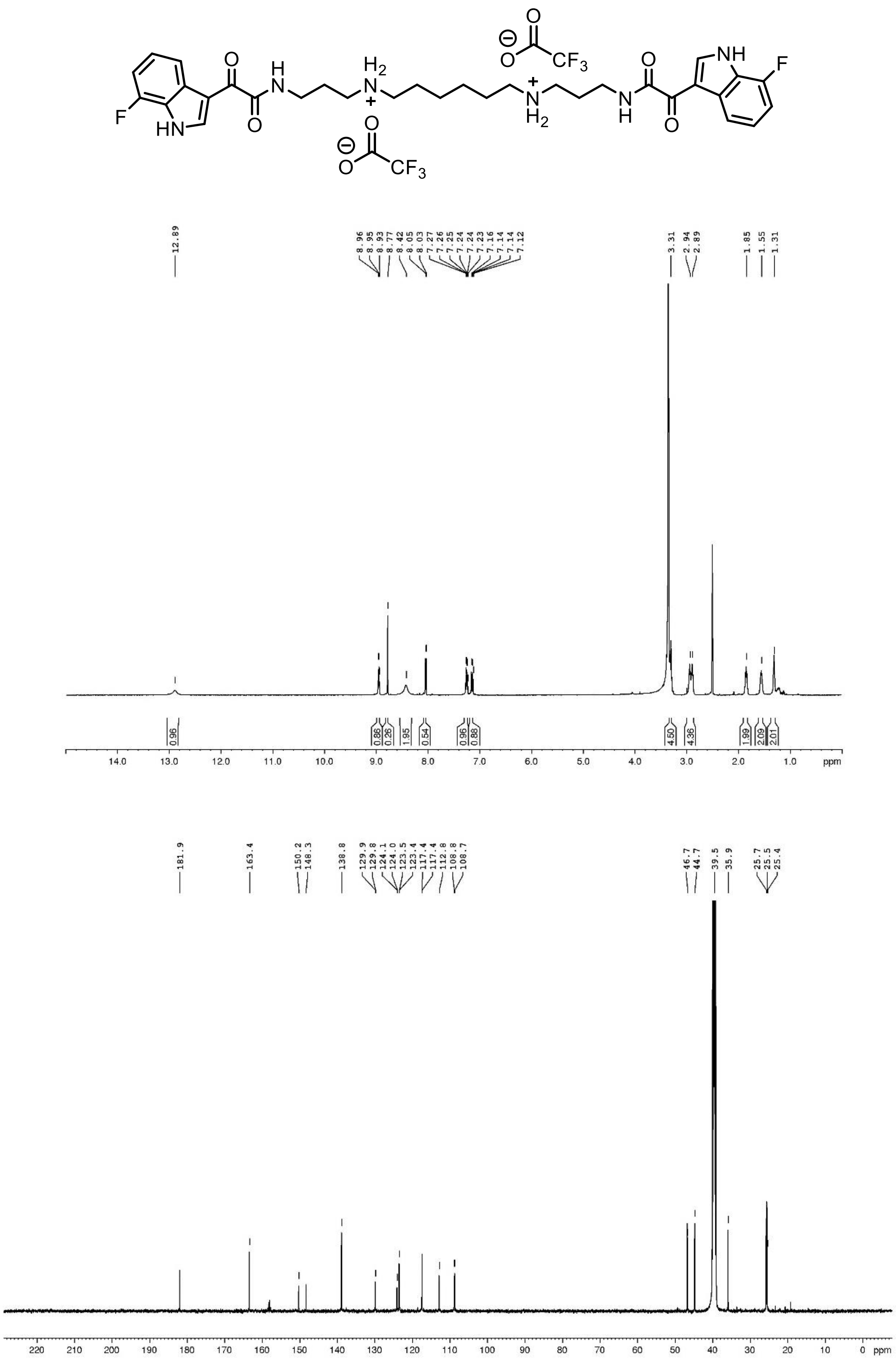

Figure S18 ^{1}H (DMSO-d_6, 500 MHz) and ^{13}C (DMSO-d_6, 125 MHz) NMR spectra for **21a**

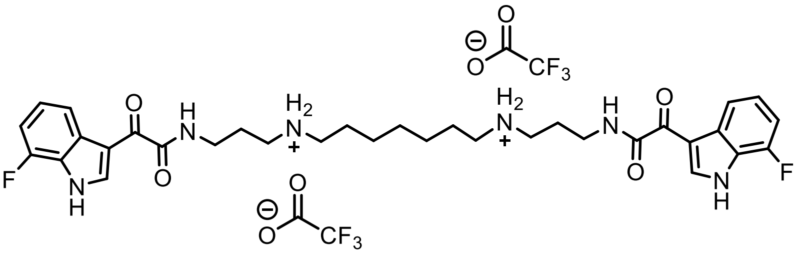

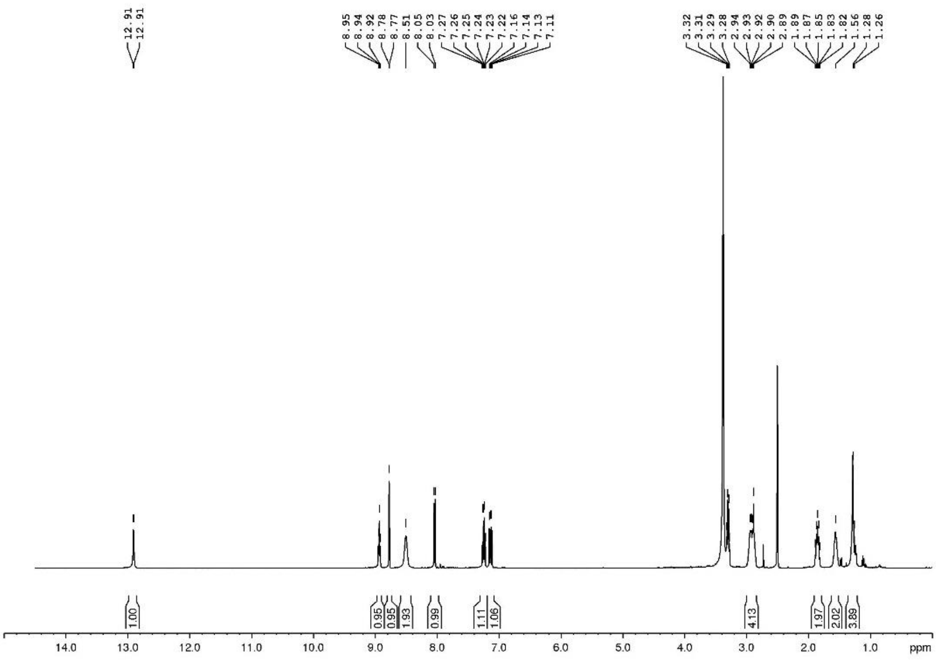

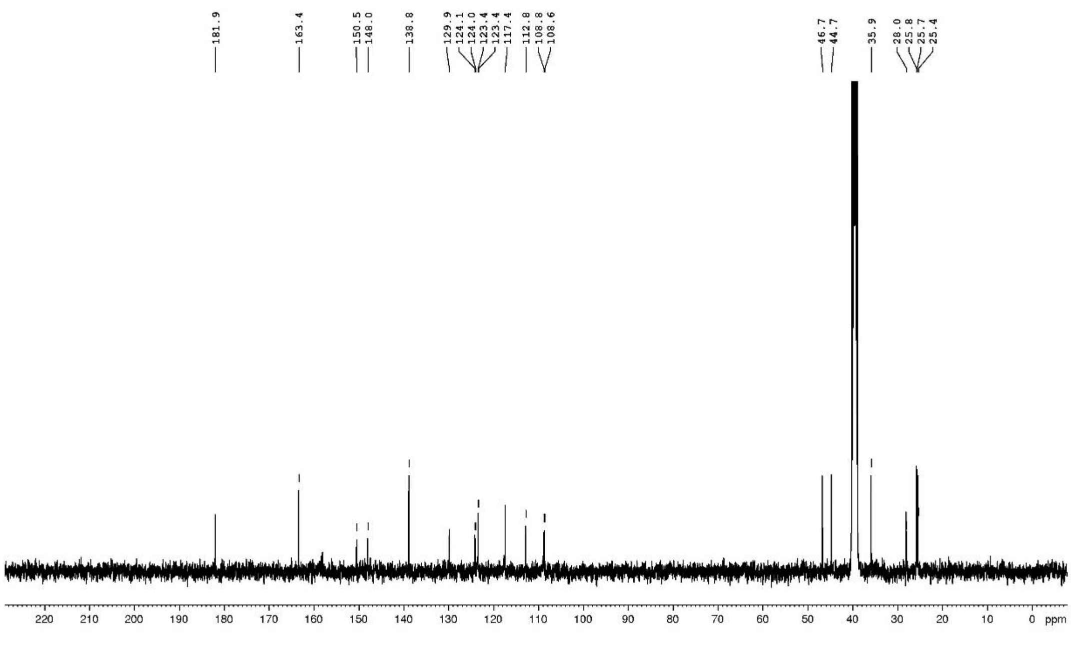

Figure S19 ^{1}H (DMSO-d_6, 400 MHz) and ^{13}C (DMSO-d_6, 100 MHz) NMR spectra for **21b**

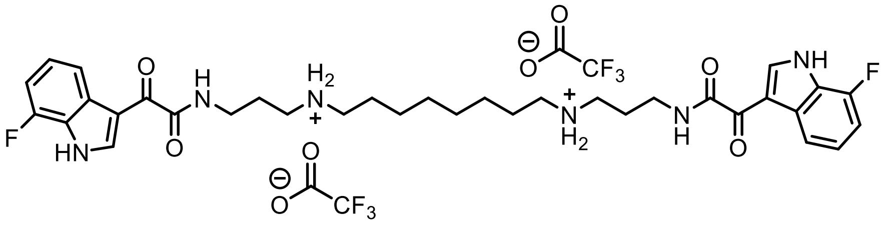

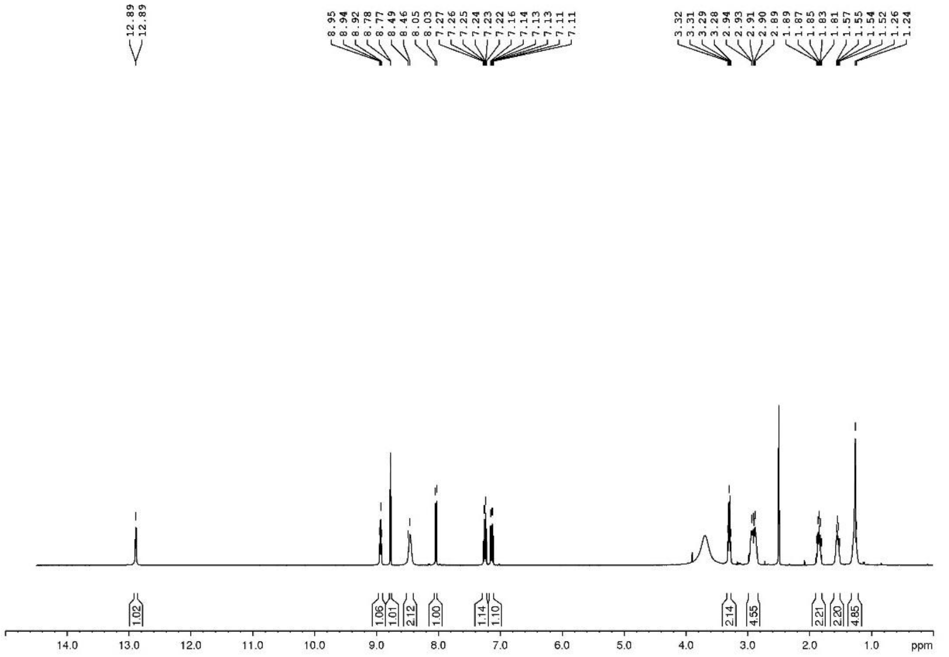

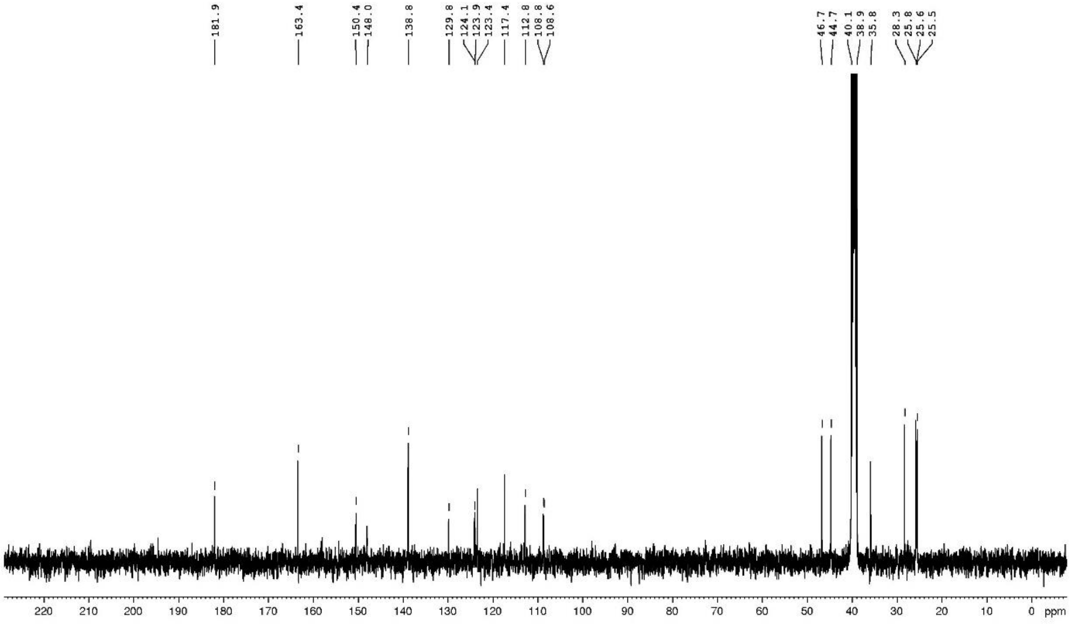

Figure S20 ^{1}H (DMSO-d_6, 400 MHz) and ^{13}C (DMSO-d_6, 100 MHz) NMR spectra for **21c**

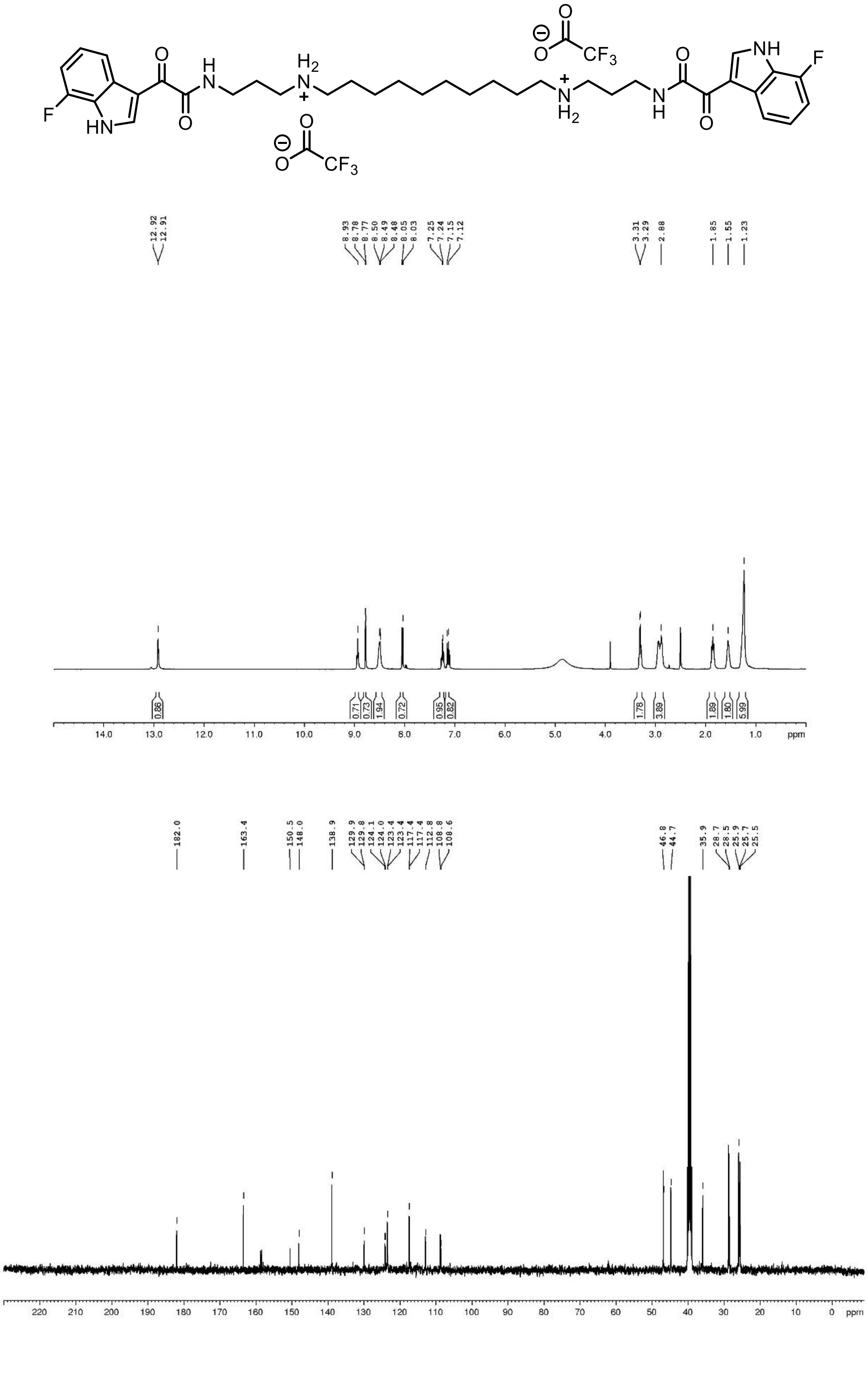

Figure S21 ^{1}H (DMSO-d_6, 400 MHz) and ^{13}C (DMSO-d_6, 100 MHz) NMR spectra for **21d**

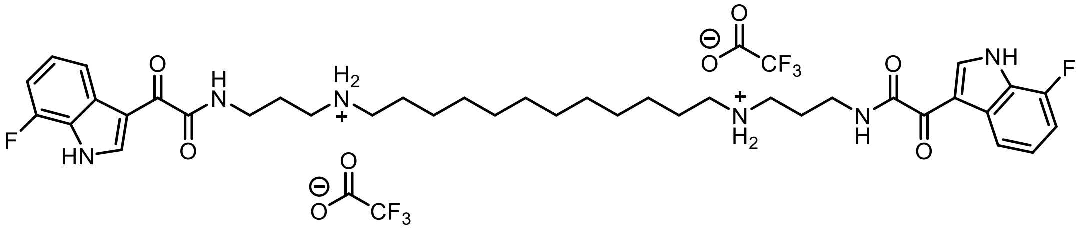

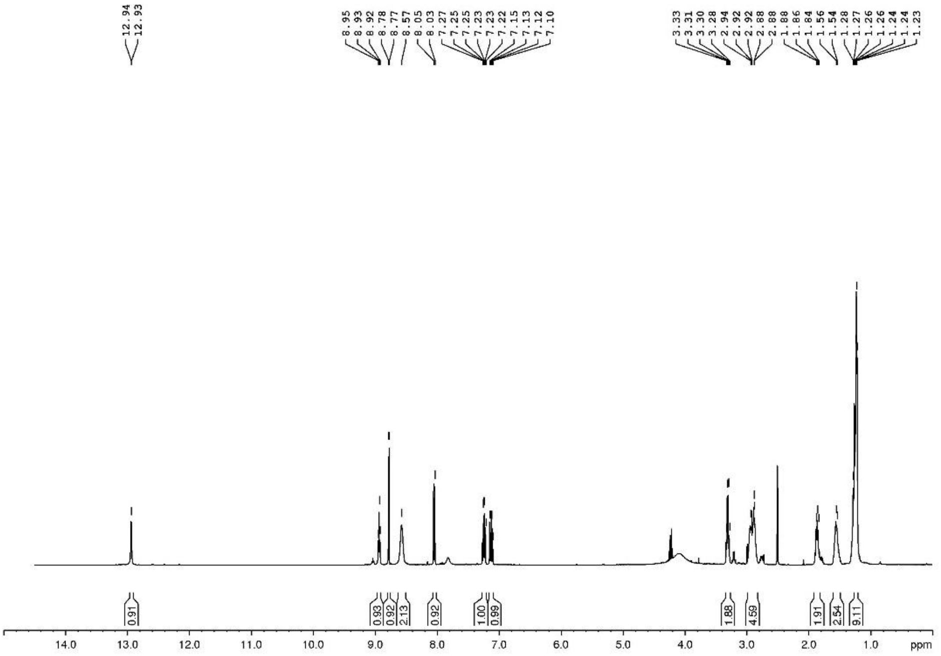

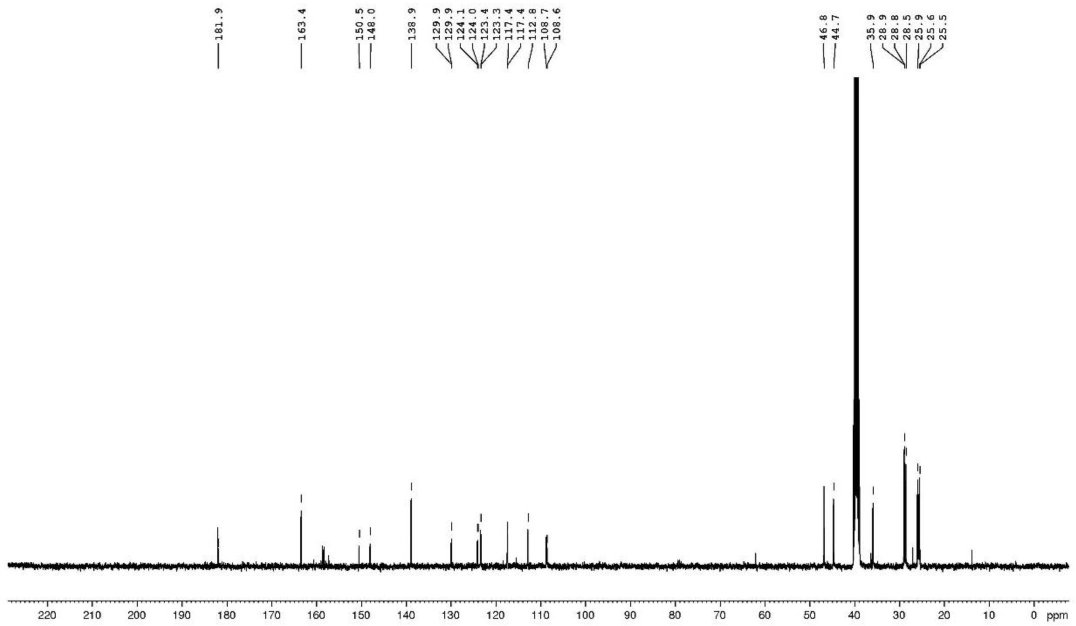

Figure S22 ^{1}H (DMSO-d_6, 400 MHz) and ^{13}C (DMSO-d_6, 100 MHz) NMR spectra for **21e**

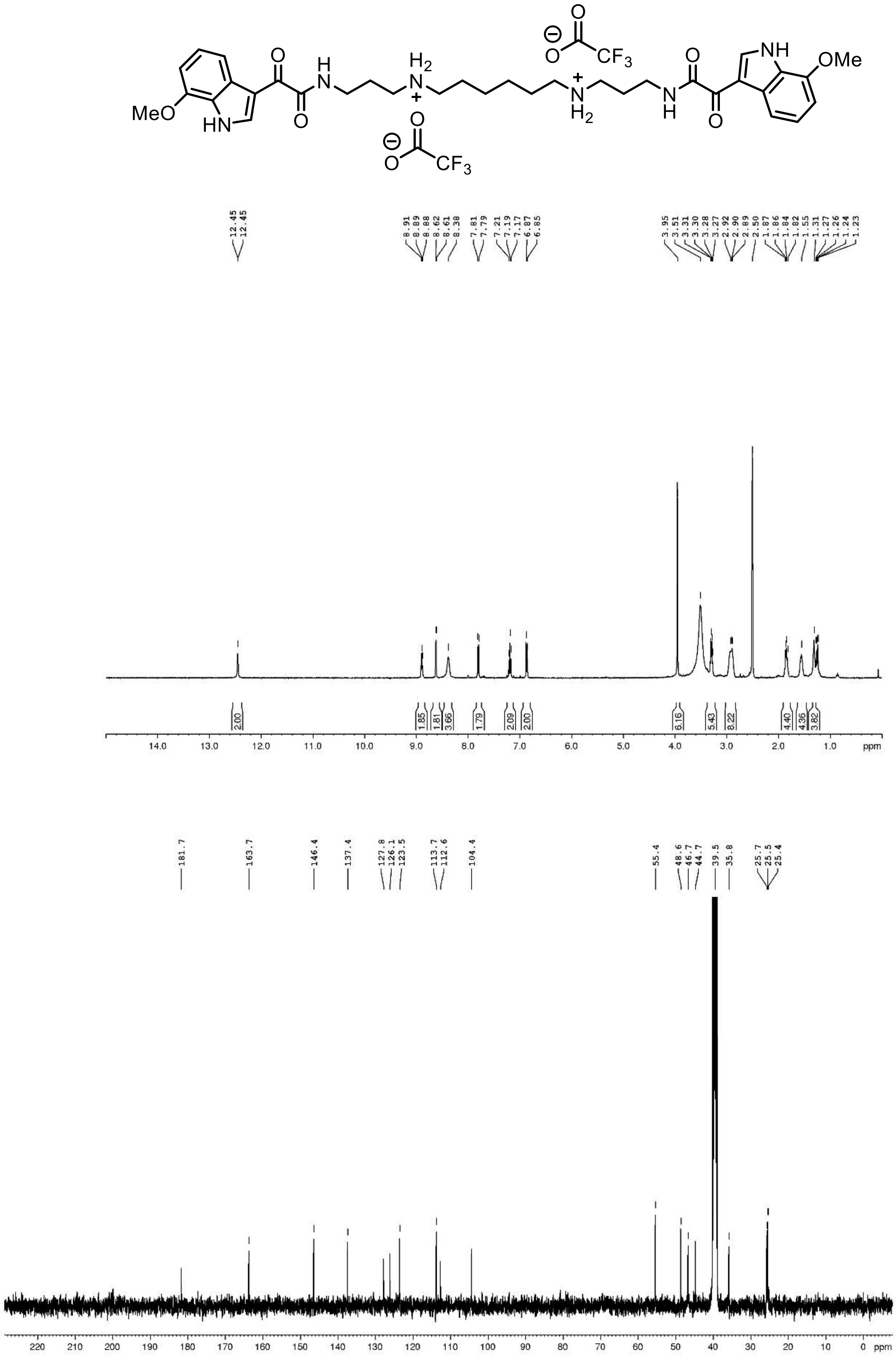

Figure S23 ^{1}H (DMSO-d_6, 400 MHz) and ^{13}C (DMSO-d_6, 100 MHz) NMR spectra for **22a**

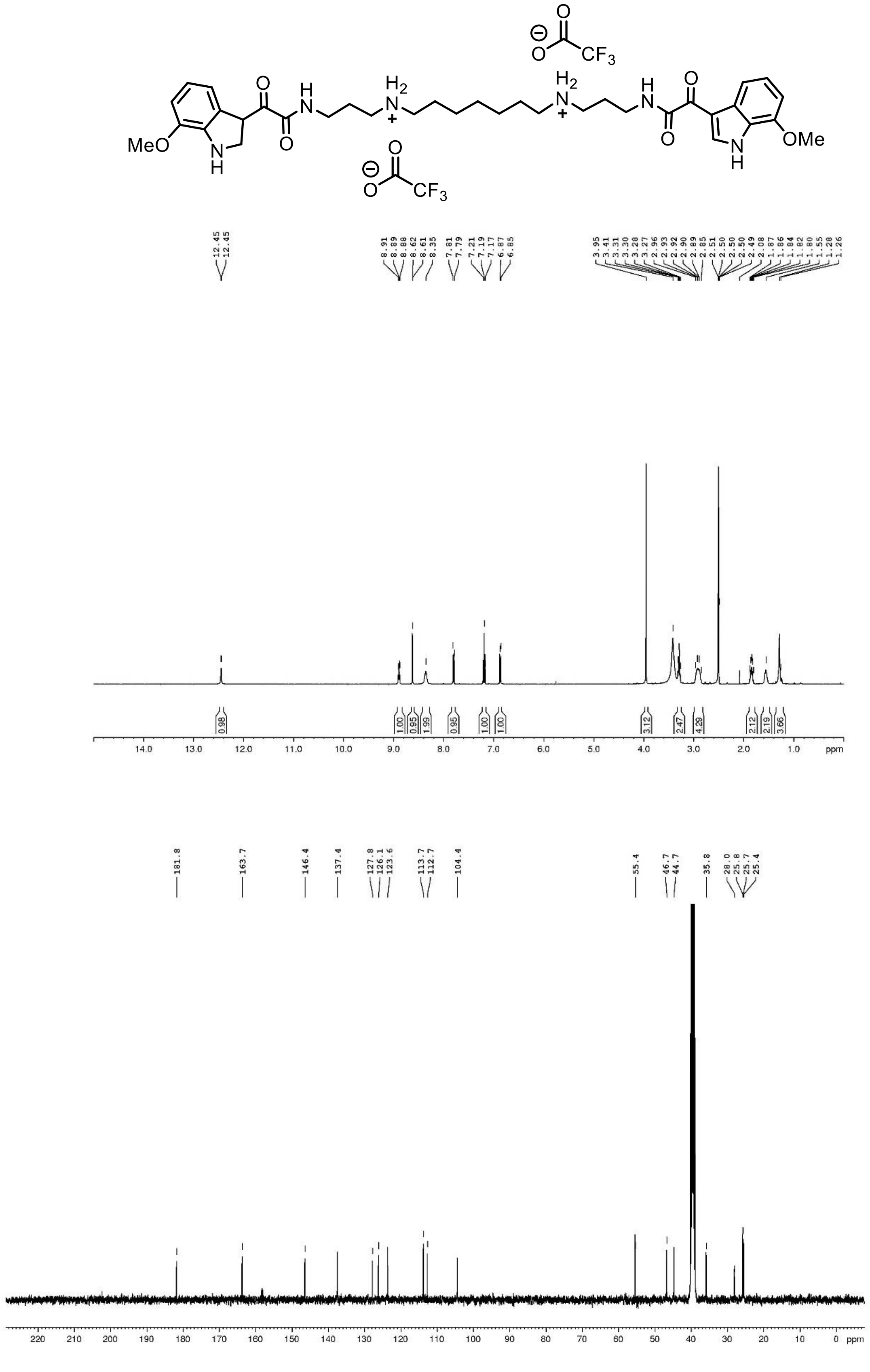

Figure S24 ^{1}H (DMSO-d_6, 400 MHz) and ^{13}C (DMSO-d_6, 100 MHz) NMR spectra for **22b**

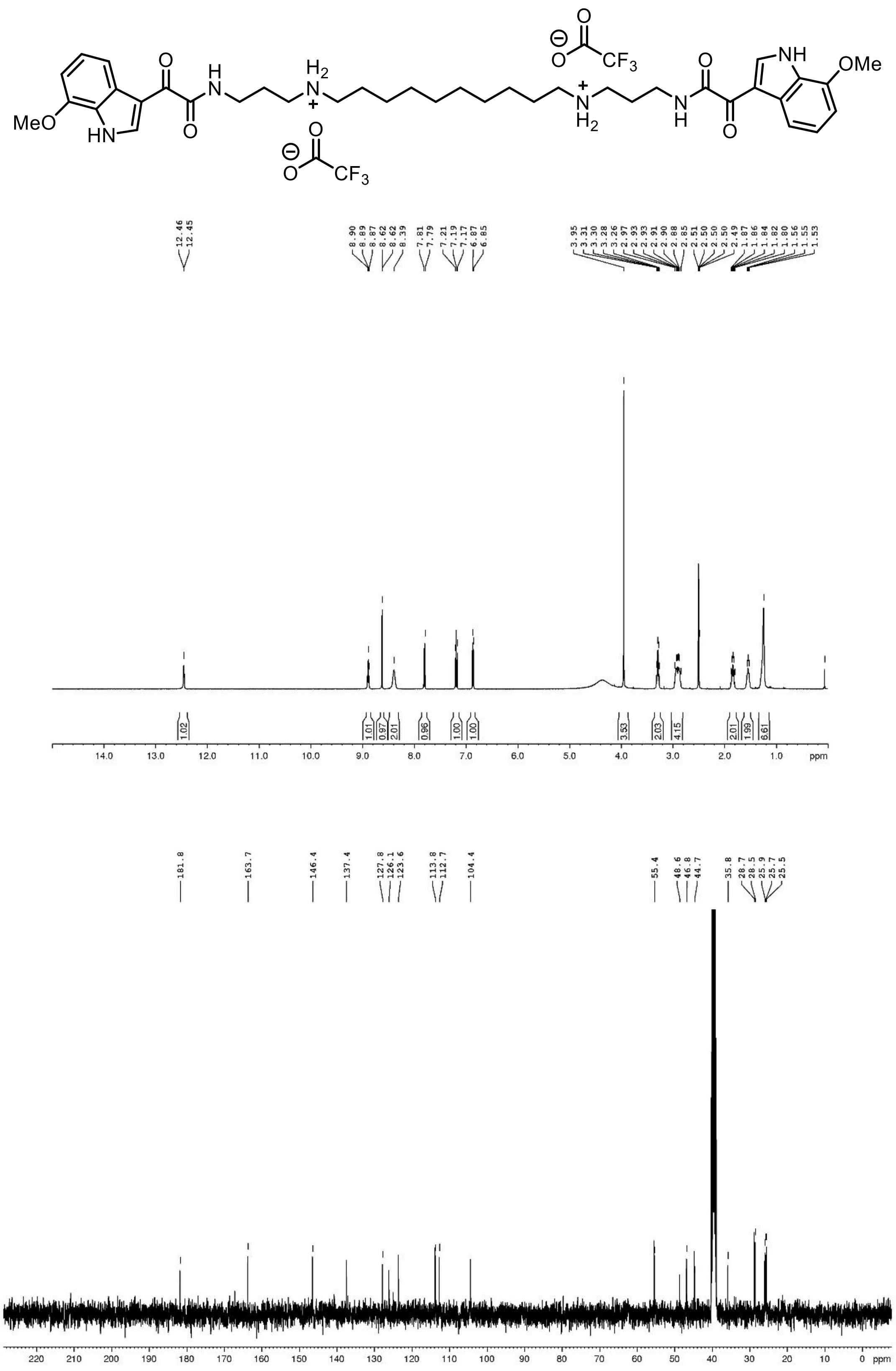

Figure S25 ^{1}H (DMSO-d_6, 400 MHz) and ^{13}C (DMSO-d_6, 100 MHz) NMR spectra for **22d**

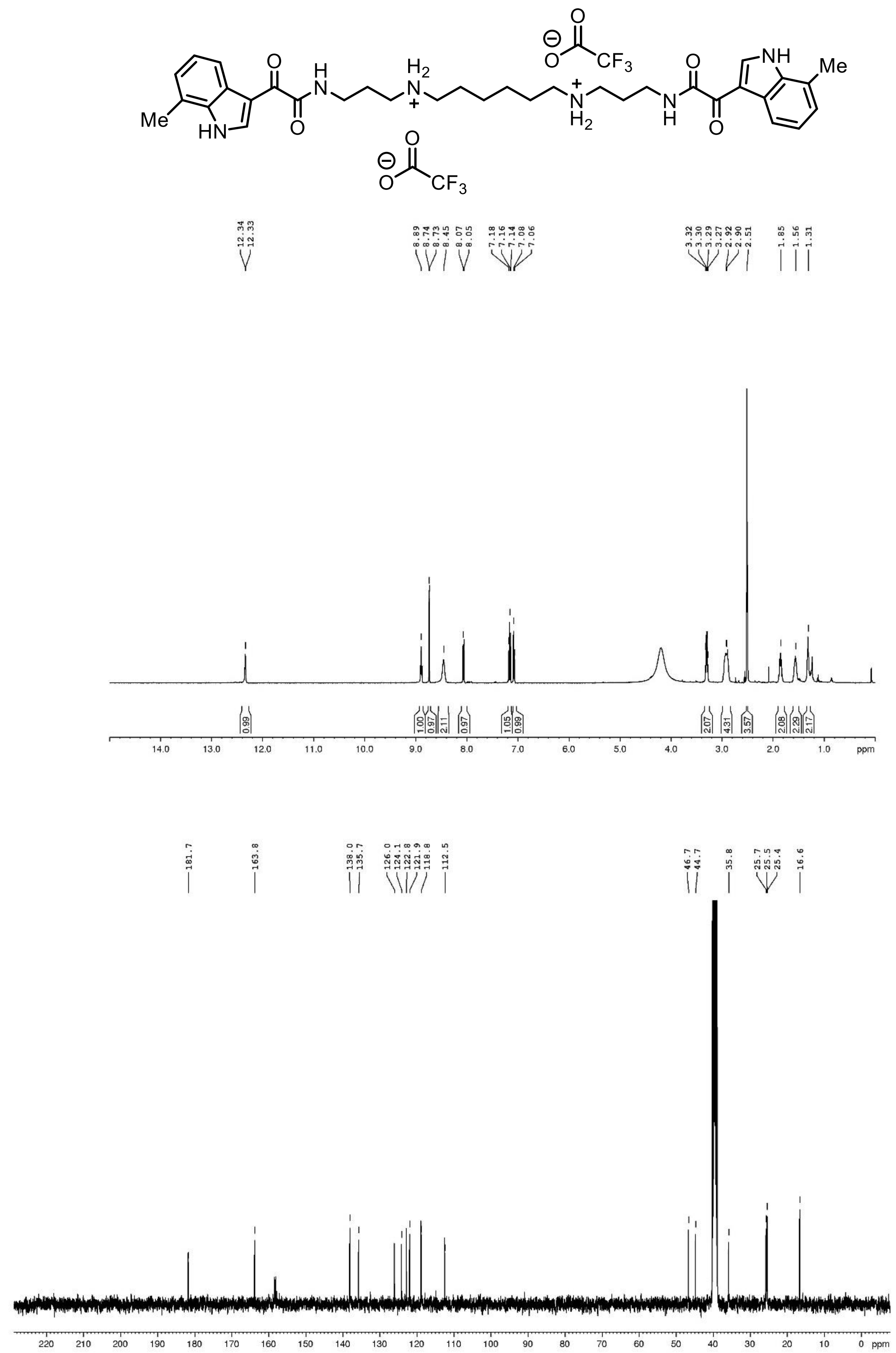

Figure S26 ^{1}H (DMSO-d_6, 400 MHz) and ^{13}C (DMSO-d_6, 100 MHz) NMR spectra for **23a**

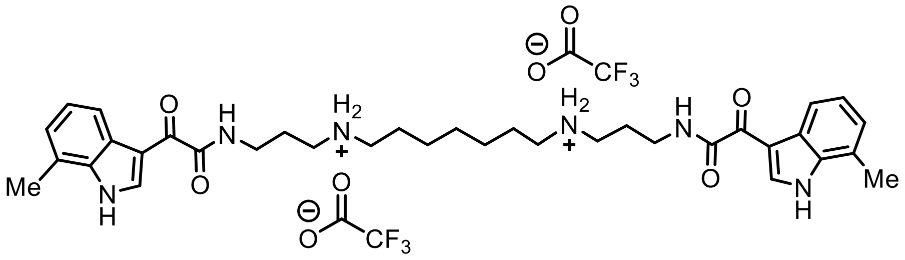

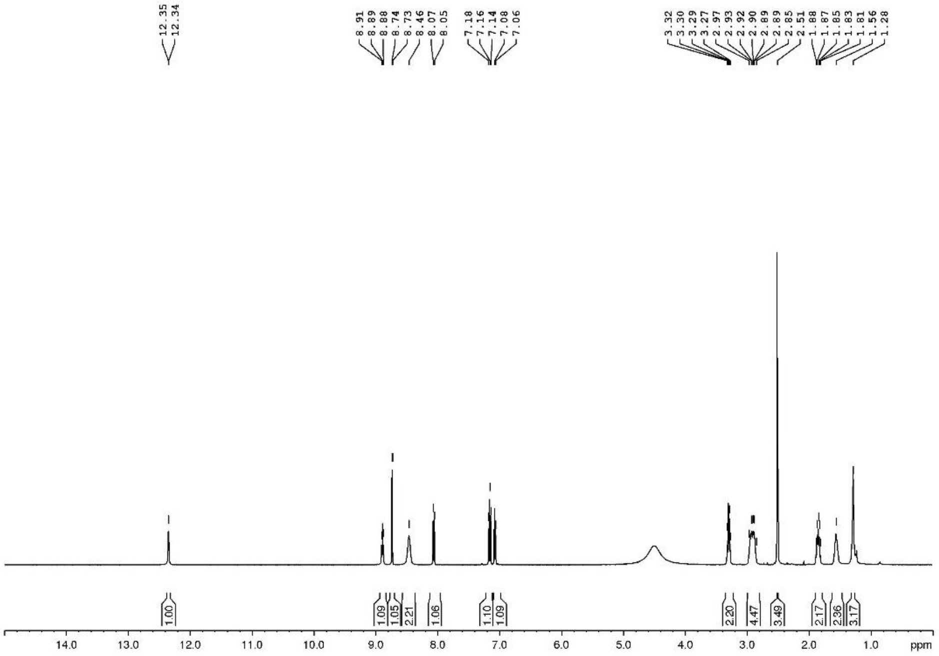

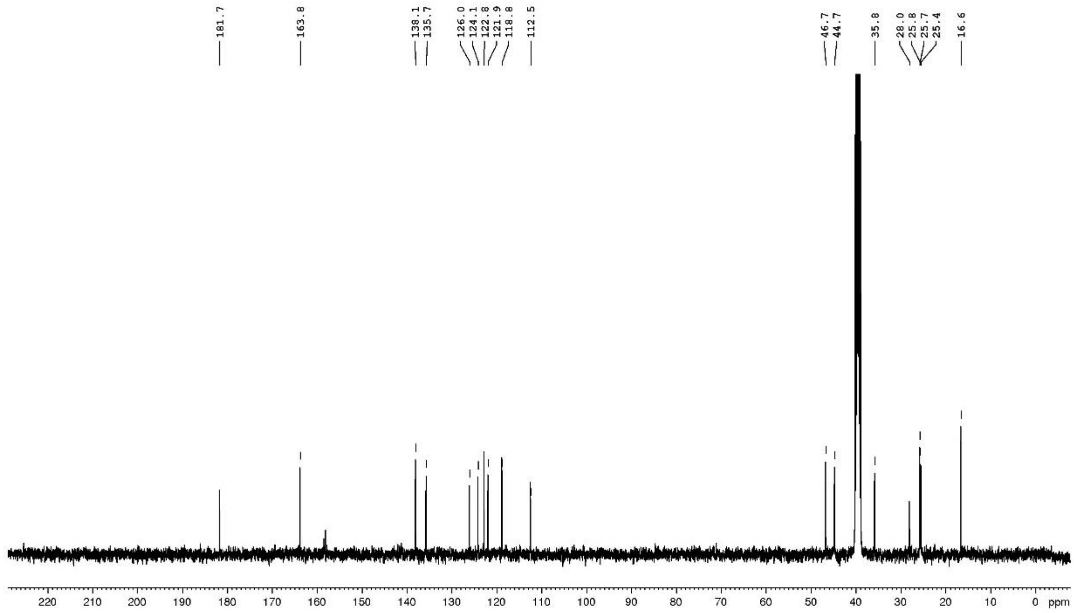

Figure S27 ^{1}H (DMSO-d_6, 400 MHz) and ^{13}C (DMSO-d_6, 100 MHz) NMR spectra for **23b**

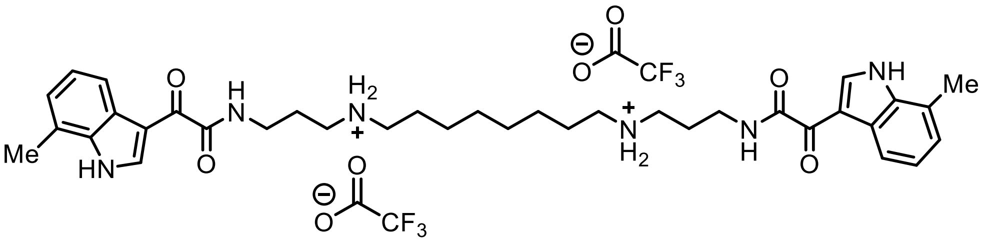

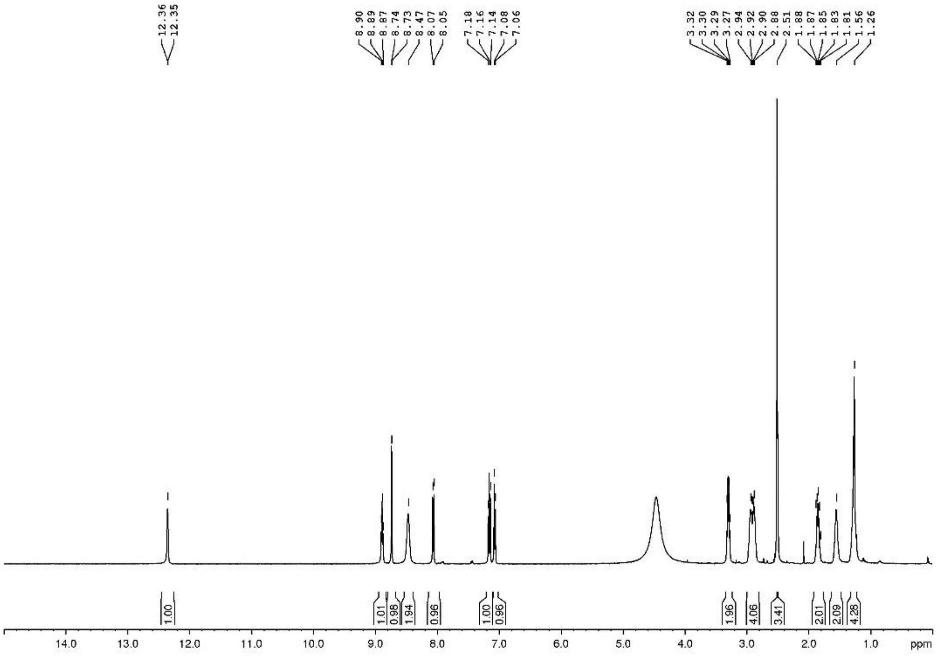

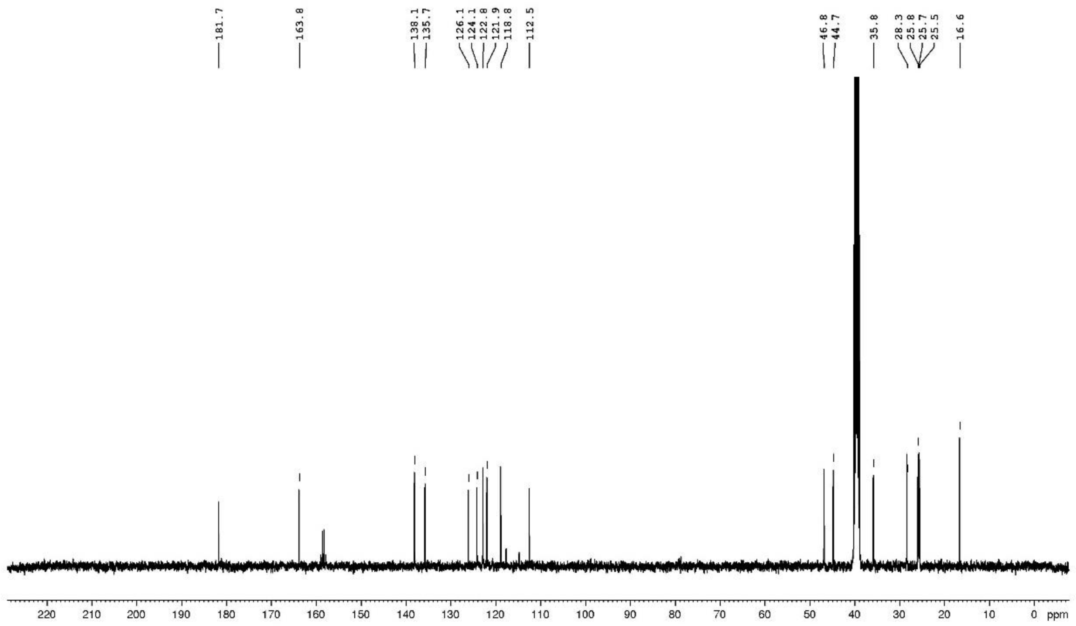

Figure S28 ^{1}H (DMSO-d_6, 400 MHz) and ^{13}C (DMSO-d_6, 100 MHz) NMR spectra for **23c**

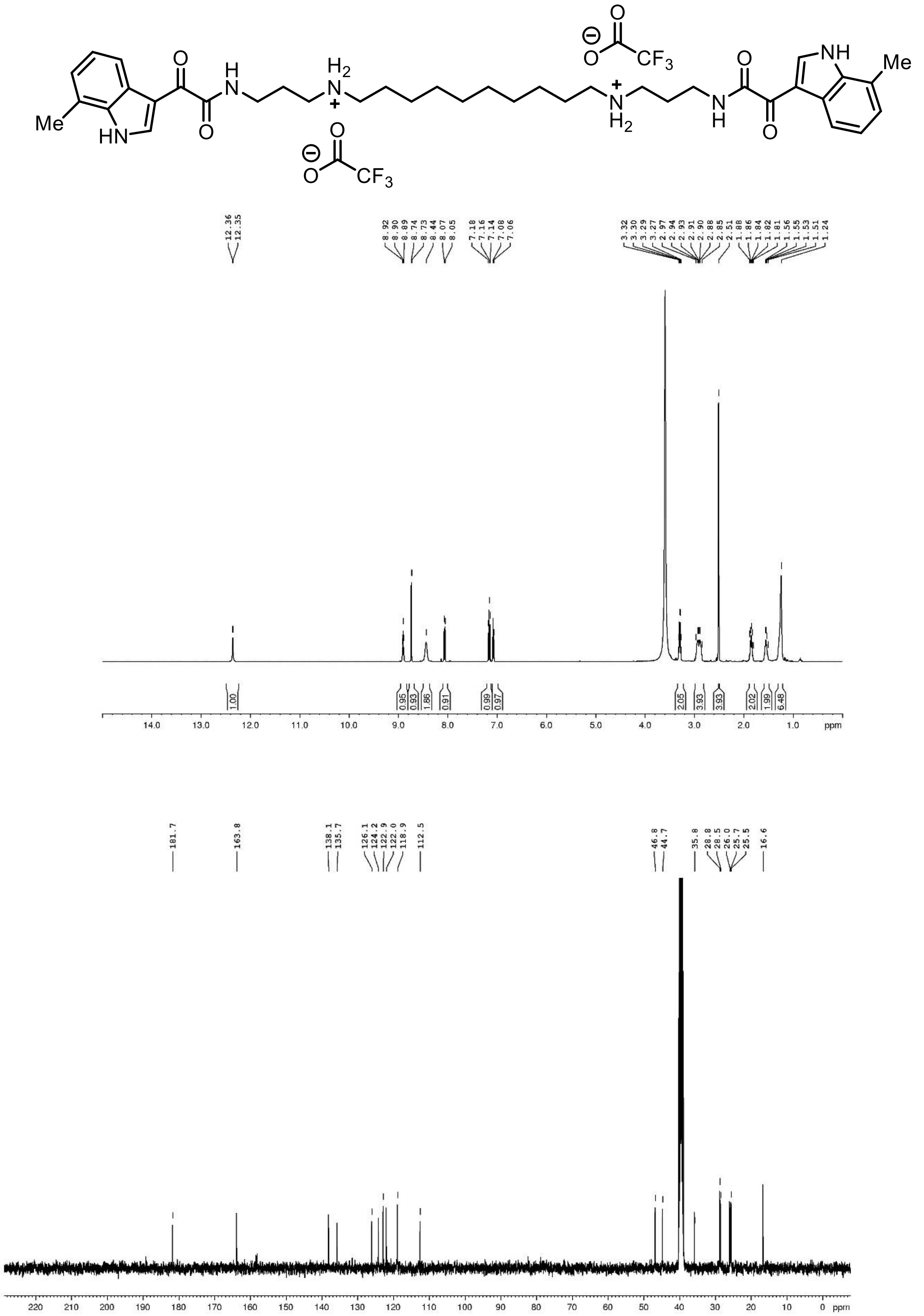

Figure S29 ^{1}H (DMSO-d_6, 400 MHz) and ^{13}C (DMSO-d_6, 100 MHz) NMR spectra for **23d**

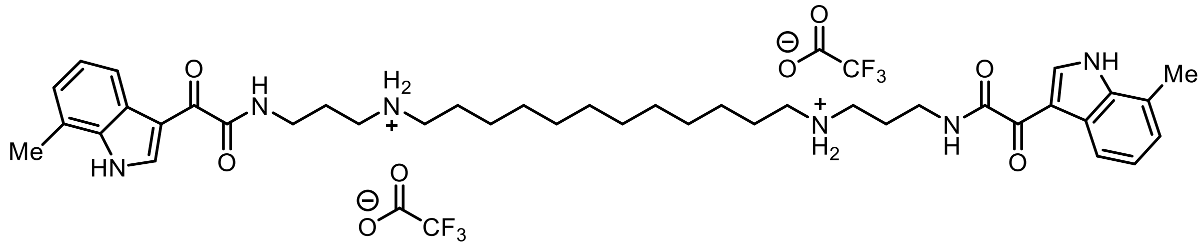

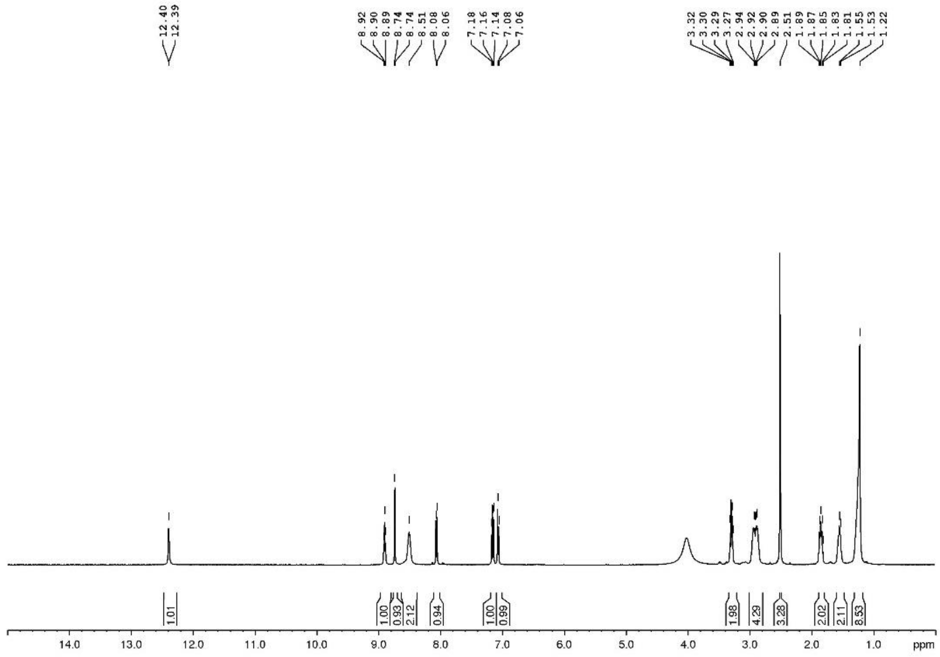

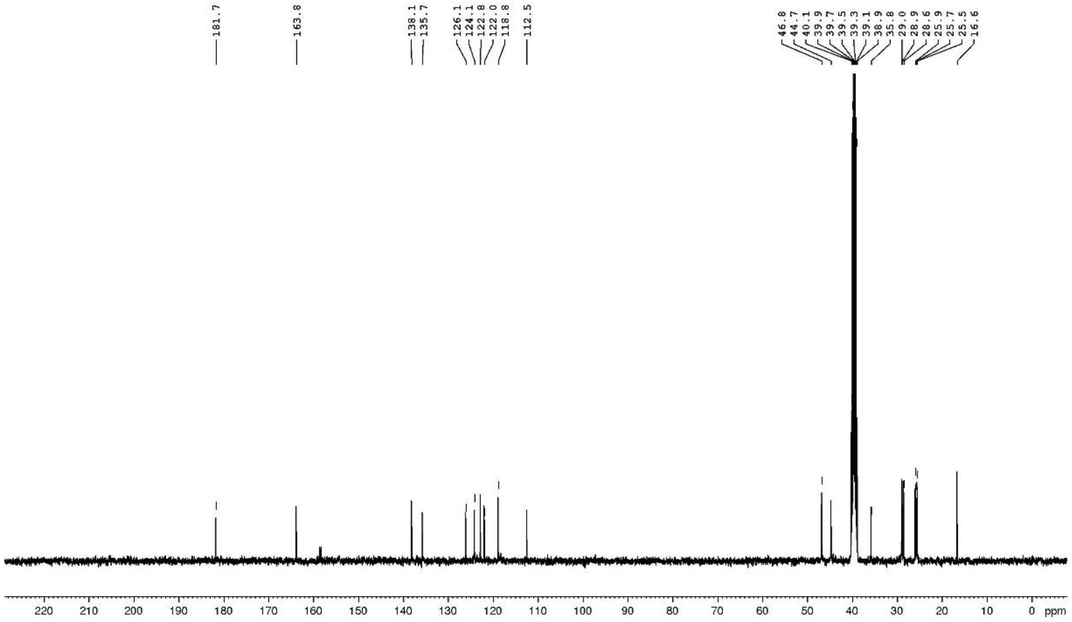

Figure S30 ^{1}H (DMSO-d_6, 400 MHz) and ^{13}C (DMSO-d_6, 100 MHz) NMR spectra for **23e**
